# Associations of the triglyceride-glucose index and its obesity-related derivatives with cardiac structure, function, and incident atrial fibrillation: a prospective cohort study using cardiac magnetic resonance

**DOI:** 10.1186/s12933-026-03143-x

**Published:** 2026-03-31

**Authors:** Zhixing Fan, Tonghuan Shi, Chaojun Yang, Tao Zheng, Marcel Sieme, Benjamin Sasko, Jan Wintrich, Muchtiar Khan, Xinyi Liu, Assem Aweimer, Andreas Mügge, Pasquale Maffia, Pierpaolo Pellicori, Ibrahim Akin, Loek van Heerebeek, Ibrahim El-Battrawy, Ulrich Schotten, Giuseppe Danilo Norata, Francesco Paneni, Jian Yang, Nazha Hamdani

**Affiliations:** 1 Department of Cardiology, The First College of Clinical Medical Sciences, China Three Gorges University, 183 Yiling Avenue, Yichang, 443003 China; 2 Hubei Key Laboratory of Ischemic Cardiovascular Disease, Yichang, 443003 China; 3 Hubei Provincial Clinical Research Center for Ischemic Cardiovascular Disease, Yichang, 443003 China; 4 Ruhr-University Bochum, Medical Faculty, Institute of Physiology, Department of Cellular and Translational Physiology, Bochum, Germany; 5 Department of Radiology, The First College of Clinical Medical Science, China Three Gorges University, Yichang, 443000 China; 6 Medical Department II, Marien Hospital Herne, Ruhr University Bochum, Bochum, Germany; 7 Department of Cardiology, OLVG, Amsterdam, The Netherlands; 8 Department of Cardiology and Angiology, University Hospital Bergmannsheil, Ruhr University Bochum, Bochum, Germany; 9 School of Infection and Immunity, College of Medical, Veterinary and Life Sciences, University of Glasgow, 120 University Place, Glasgow, G12 8TA UK; 10 Department of Pharmacy, School of Medicine and Surgery, University of Naples Federico II, 80131 Naples, Italy; 11 School of Cardiovascular and Metabolic Health, College of Medical, Veterinary and Life Sciences, University of Glasgow, Glasgow, UK; 12 First Department of Medicine, University Medical Centre Mannheim (UMM), Mannheim, Germany; 13 Medizinische Klinik II, Klinikum der Ruhr-Universität Bochum, Kardiologie, Bochum, Germany; 14 Department of Physiology, Cardiovascular Research Institute Maastricht, Maastricht University, Maastricht, The Netherlands; 15 Department of Pharmacological and Biomolecular Sciences, University of Milan, Via Balzaretti 9, Milan, Italy; 16 Center for the Study of Atherosclerosis, E. Bassini Hospital, Via Massimo Gorki 50 , Cinisello Balsamo, Italy; 17 Center for Translational and Experimental Cardiology (CTEC), Zurich University Hospital, University of Zurich, Zurich, Switzerland; 18 University Heart Center, University Hospital Zurich, University of Zurich, Zurich, Switzerland

**Keywords:** Triglyceride-glucose index, Atrial fibrillation, Cardiac magnetic resonance, Body mass index, Waist circumference, Waist-to-height ratio, Cardiac structure, Cardiac function

## Abstract

**Background:**

The triglyceride-glucose (TyG) index and its obesity-related derivatives have emerged as surrogate markers of insulin resistance associated with cardiovascular outcomes. However, whether these indicators influence atrial fibrillation (AF) risk through cardiac structural and functional remodeling remains unclear.

**Methods:**

This is a post-hoc analysis of a prospective cohort study of 32,500 UK Biobank participants free of AF who underwent baseline cardiac magnetic resonance (CMR) imaging. The TyG index and its obesity-related derivatives (TyG-body mass index (BMI), TyG-waist circumference (WC), and TyG-waist-to-height ratio (WHtR)) were calculated at baseline. Multivariable Cox regression models were used to assess associations with incident AF, and mediation analyses quantified the contribution of CMR-derived cardiac parameters to these associations.

**Results:**

Over a median follow-up of 13.61 years, 1,288 incident AF cases occurred. The TyG index alone showed no independent association with AF risk. In contrast, all TyG obesity-related derivatives were significantly associated with incident AF, with TyG-WC demonstrating the strongest association (HR = 1.245, 95% CI 1.169–1.325), followed by TyG-BMI (HR = 1.223, 95% CI 1.158–1.293) and TyG-WHtR (HR = 1.190, 95% CI 1.122–1.262). Mediation analyses identified left atrial maximum volume (LAVmax) as the predominant mediator, accounting for 70.63%, 47.83%, and 40.79% of the associations for TyG-BMI, TyG-WHtR, and TyG-WC, respectively.

**Conclusions:**

TyG obesity-related derivatives, particularly TyG-WC, were independently associated with incident AF. Cardiac structural remodeling, especially LA enlargement, appeared to be a key mediating pathway. These findings support the importance of early metabolic intervention to prevent adverse atrial remodeling and reduce AF susceptibility.

**Graphical Abstract:**

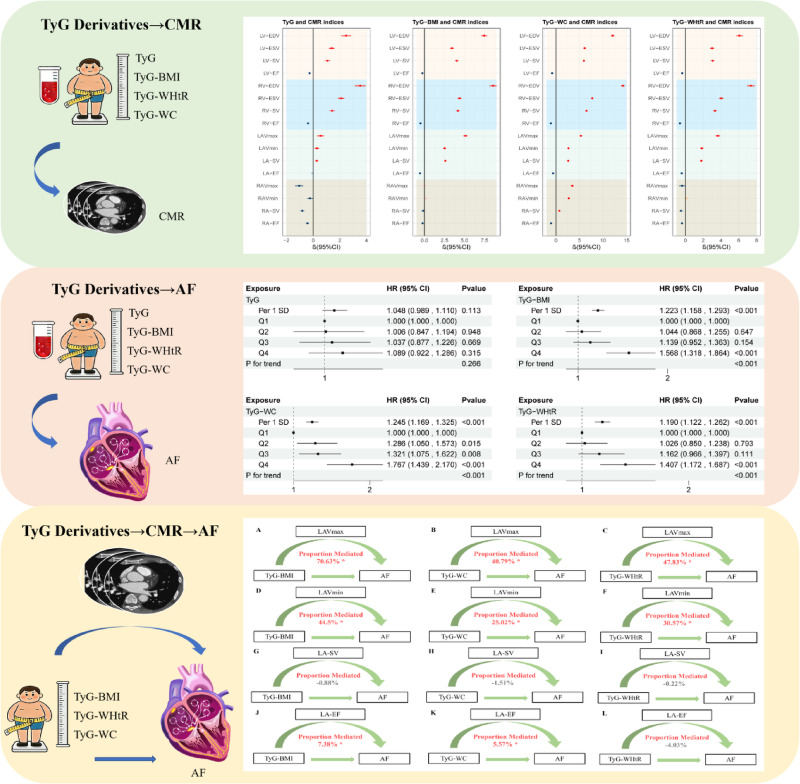

**Supplementary Information:**

The online version contains supplementary material available at 10.1186/s12933-026-03143-x.

## Research Insights

**What is currently known about this topic?**

- TyG index is a surrogate marker for insulin resistance.
- An elevated TyG index is associated with increased AF risk.
- Insulin resistance adversely affects cardiac structure and function.

**What is the key research question?**

- Do TyG obesity-related indicators influence AF risk through cardiac structural remodeling?

**What is new?**

- TyG obesity-related derivatives outperform TyG alone in predicting AF.
- TyG-WC shows the strongest association with incident AF.
- Left atrial enlargement mediates up to 70% of the association between TyG derivatives and AF.

**How might this study influence clinical practice?**

- TyG-WC may serve as a low-cost and readily accessible tool for AF risk stratification.

## Introduction

Atrial fibrillation (AF) is the most prevalent cardiac arrhythmia worldwide and is associated with substantial disability, morbidity, and mortality [1, 2]. The prevalence of AF is increasing, mostly due to population aging and the high prevalence of established risk factors, for instance obesity, diabetes and hypertension, and alcohol consumption [3]. Left atrial (LA) enlargement and dysfunction are hallmark features of AF, with increased LA volume and reduced emptying fraction independently predicting incident AF in the general population and across a broad range of cardiovascular conditions, including heart failure [4–6]. Structural and functional biventricular impairment, and right atrial (RA) dilatation are also associated with LA dilatation and dysfunction, and might contribute to incident AF, underscoring the importance of comprehensive cardiac assessment in understanding AF pathophysiology [7, 8].

Insulin resistance (IR) is thought to have detrimental effects on cardiac structure and function [9]. Evidence from cardiac magnetic resonance (CMR) imaging studies suggests that IR is associated with increased left ventricular mass, concentric remodeling, and impaired diastolic function [10, 11]. The triglyceride-glucose (TyG) index, calculated from triglycerides and glucose, has emerged as a simple, reliable, and low-cost surrogate marker of insulin resistance (IR) [12]. Some preliminary reports suggest that a higher TyG index is associated with an increased risk of various cardiovascular outcomes, including heart failure and AF [12–15]. TyG obesity-related derivatives that incorporate anthropometric measures, such as TyG-body mass index (TyG-BMI), TyG-waist circumference (TyG-WC), and TyG-waist-to-height ratio (TyG-WHtR), have been developed and validated as more robust risk markers for improving risk stratification [16–19]. Whether the TyG index and its derivatives influence AF risk through cardiac structural and functional remodeling remains unknown. Therefore, we conducted a post hoc analysis of the UK Biobank prospective cohort to investigate the associations of the TyG index and its derivatives with cardiac structure and function assessed by CMR, and incident AF, and to explore the potential mediating role of cardiac remodeling in the pathway from TyG obesity-related derivatives to AF.

## Method

### Study population

The UK Biobank is a population-based prospective cohort that enrolled approximately 500,000 individuals between 40 and 69 years of age from 22 recruitment centers throughout England, Wales, and Scotland between 2006 and 2010. Baseline data collection encompassed standardized touchscreen questionnaires, anthropometric measurements, and the collection of biological specimens. A subset of participants underwent CMR imaging as part of the UK Biobank imaging sub-study, which commenced in 2014. Comprehensive descriptions of the UK Biobank protocol and methodology have been published elsewhere [20]. This study received ethical approval from the North West Multi-Centre Research Ethics Committee (reference 16/NW/0274), with all participants providing written informed consent prior to enrollment. The current analysis was performed under UK Biobank application number 170,605.

For the present analysis, we initially identified 45,536 participants who underwent CMR examinations from the UK Biobank cohort of 502,132 individuals. Participants were excluded if they had missing cardiac structure and function data (n = 7042) or lacked information required to calculate TyG and its obesity-related derivatives (TyG-BMI, TyG-WC, and TyG-WHtR) (n = 5721). After these exclusions, 32,773 participants with complete CMR and metabolic data remained. We further excluded individuals with a history of AF at baseline (n = 273). Ultimately, 32,500 participants were included in the final analytical sample. The detailed participant selection process is illustrated in Figure S1.

### CMR

CMR scans were acquired according to a standardized imaging protocol utilizing 1.5 Tesla wide-bore scanners (MAGNETOM Aera, Syngo Platform VD13A, Siemens Healthcare, Erlangen, Germany) [21]. The acquisition protocol included standard long-axis views and a short-axis cine stack encompassing both ventricles from base to apex, obtained using balanced steady-state free precession sequences. Image analysis was performed with cvi42 post-processing software (Version 5.1.1, Circle Cardiovascular Imaging Inc., Calgary, Canada) with a fully automated pipeline and a quality-control verification [22]. The CMR-derived parameters included structural and functional measurements of all four cardiac chambers. For the left ventricle (LV) and right ventricle (RV), we assessed end-diastolic volume (EDV), end-systolic volume (ESV), stroke volume (SV), and ejection fraction (EF). For the LA and RA, maximum volume (Vmax), minimum volume (Vmin), SV, and EF were also measured.

### TyG and its obesity-related derivatives

Non-fasting blood samples were collected at the baseline assessment, and serum triglyceride and plasma glucose concentrations were measured using standardized laboratory procedures. Anthropometric measurements, including height, weight, and waist circumference, were obtained by trained staff following standardized protocols. The TyG index and its derivatives were calculated using the following formulas:

$$ {\text{TyG = ln}}\,\,[{\mathrm{triglycerides}}\,({\mathrm{mg/dL}}) \times {\text{glucose }}\left( {{\mathrm{mg}}/{\mathrm{dL}}} \right)/2]; $$

$$ {\text{TyG - BMI = TyG }} \times \,\,{\text{BMI }}\left( {{\mathrm{kg}}/{\mathrm{m}}} \right);{\text{ }} $$

$$ {\text{TyG - WC }} = {\text{ TyG }} \times {\text{ waist circumference }}\left( {{\mathrm{cm}}} \right); $$

$$ {\text{TyG - WHtR = TyG}} \times {\text{waist - to - height ratio}} $$

BMI was calculated as weight (kg) divided by height squared (m²), and the waist-to-height ratio was derived by dividing waist circumference (cm) by height (cm).

### Atrial fibrillation

Incident AF was identified through multiple data sources within the UK Biobank. These included self-reported non-cancer illness codes, hospital inpatient diagnoses (ICD-9 and ICD-10), primary and secondary causes of death records (ICD-10), and operative procedure codes (OPCS-4) related to AF ablation and cardioversion. Participants were followed from the date of CMR examination until the first diagnosis of AF, death, loss to follow-up, or the end of the follow-up period (December 31, 2022), whichever occurred first. Individuals with a documented history of AF prior to CMR assessment were excluded from the analysis. The detailed diagnostic criteria for AF are presented in Table S1.

### Covariates

Covariates were selected based on prior literature and potential confounding effects on the associations between TyG and its obesity-related derivatives, cardiac structure and function, and incident AF. Sociodemographic variables included age (years), sex (male or female), race (White or other), education (university degree or no university degree), and household income (<£18,000 or ≥£18,000 per year). Lifestyle factors included smoking status (never, former, or current), alcohol consumption (never, former, or current), and physical activity level (low, moderate, or high). Dietary pattern was evaluated using the Dietary Approaches to Stop Hypertension (DASH) score, a validated composite measure reflecting adherence to a diet rich in fruits, vegetables, whole grains, and low-fat dairy while limiting sodium and saturated fat intake, categorized as adherent or non-adherent. Medical history variables included diabetes mellitus, hypertension, other cardiovascular diseases (CVD, including ischemic heart disease, heart failure, and stroke), and cancer. Polygenic risk score (PRS) for AF, summarizing inherited susceptibility by weighting multiple single-nucleotide polymorphisms associated with AF risk, was included to account for genetic predisposition.

### Statistical analysis

Missing covariate data were addressed using multiple imputation by chained equations. Baseline characteristics were summarized as mean ± standard deviation (SD) for continuous variables and frequency (percentage) for categorical variables. Group comparisons were performed using Student’s t-test or one-way analysis of variance for continuous data and chi-square test for categorical data.

Spearman correlation coefficients were calculated to assess correlations between TyG derivatives and CMR-derived cardiac parameters. Multivariable linear regression models were fitted to examine the associations of TyG derivatives with cardiac structural and functional indices, reporting β with corresponding 95% confidence intervals (CIs). Cox proportional hazards models were used to evaluate the relationships of CMR parameters and TyG derivatives with incident AF, reporting hazard ratios (HRs) with corresponding 95% CIs. Covariates were adjusted using three hierarchical models: Model 1 included age, sex, ethnicity, educational attainment, and household income; Model 2 incorporated Model 1 variables plus physical activity, smoking status, alcohol consumption, DASH score, and PRS; Model 3 extended Model 2 by adding history of diabetes mellitus, hypertension, other CVD, and cancer. All TyG derivatives and CMR parameters were standardized per 1-SD increment (z-score transformation) in the regression models. Restricted cubic spline (RCS) analysis was applied to detect potential non-linear associations between exposures and AF risk.

Causal mediation analysis based on the counterfactual framework was performed to investigate whether cardiac structure and function served as mediators in the associations between TyG derivatives and incident AF. In this framework, the total effect of an exposure on an outcome is decomposed into a natural direct effect (the effect of the exposure on the outcome not operating through the mediator) and a natural indirect effect (the effect operating through the mediator), from which the proportion mediated is derived. The mediation analysis relies on the assumptions of temporal ordering and the absence of unmeasured confounding. This approach has been increasingly applied in cardiovascular epidemiology to quantify pathways linking metabolic exposures to cardiac outcomes [23]. This analysis was implemented using the CMAverse package in R.

Subgroup analyses were undertaken across the population with different sociodemographic characteristics, lifestyle factors, and medical history, and interaction effects were assessed by likelihood ratio tests. The robustness of findings was verified through three sensitivity analyses: (1) restricting to participants with complete data without imputation; (2) excluding individuals who developed AF within the initial two years of follow-up to mitigate potential reverse causation; and (3) removing participants with prevalent CVD (ischemic heart disease, heart failure, or stroke) at baseline.

All analyses were conducted using R software (version 4.3.1, R Foundation for Statistical Computing, Vienna, Austria). Statistical significance was defined as a two-sided *P* value < 0.05.

## Results

### Baseline characteristics

Among the 32,500 participants included in the final analysis, 1,288 (3.96%) developed incident AF during a median follow-up of 13.61 years. As shown in Table 1, participants who developed AF were older, more likely to be male, and had a higher BMI compared with non-AF participants. Hypertension (40.61% vs. 21.10%), diabetes mellitus (5.12% vs. 2.67%), and other CVD (10.09% vs. 3.32%) were also more common amongst those who developed AF. Furthermore, individuals with incident AF had a greater polygenic risk score for AF.

**Table 1:** Baseline characteristics of participants with atrial fibrillation and the control group

Characteristic	Level	Overall (n = 32500)	Control (n = 31212)	AF (n = 1288)	*P*
Age (year)		54.82 (7.46)	54.62 (7.43)	59.59 (6.28)	< 0.001
	< 65	29,310 (90.18)	28,321 (90.74)	989 (76.79)	< 0.001
	≥ 65	3190 (9.82)	2891(9.26)	299 (23.21)	
Sex (%)	Female	16,845 (51.83)	16,434 (52.65)	411 (31.91)	< 0.001
	Male	15,655 (48.17)	14,778 (47.35)	877 (68.09)	
Race (%)	Other	925 (2.85)	908 (2.91)	17 (1.32)	0.001
	White	31,575 (97.15)	30,304 (97.09)	1271 (98.68)	
Education (%)	No university degree	17,390 (53.51)	16,618 (53.24)	772 (59.94)	< 0.001
	University degree	15,110 (46.49)	14,594 (46.76)	516 (40.06)	
Income (%)	<£18,000	3845 (11.83)	3642 (11.67)	203 (15.76)	< 0.001
	>£18,000	28,655 (88.17)	27,570 (88.33)	1085 (84.24)	
BMI (kg/m^2^)		26.59 (4.23)	26.54 (4.20)	27.87 (4.65)	< 0.001
	< 25	12,727 (39.16)	12,374 (39.65)	353 (27.41)	< 0.001
	25 ~ 29	13,938 (42.89)	13,357 (42.79)	581 (45.11)	
	≥ 30	5835 (17.95)	5481 (17.56)	354 (27.48)	
Physical activity (%)	Low	6066 (18.66)	5814 (18.63)	252 (19.57)	0.189
	Moderate	13,438 (41.35)	12,937 (41.45)	501 (38.90)	
	High	12,996 (39.99)	12,461 (39.92)	535 (41.54)	
Smoker (%)	Never	19,709 (60.64)	19,051(61.04)	658 (51.09)	< 0.001
	Previous	10,738 (33.04)	10,185 (32.63)	553 (42.93)	
	Current	2053 (6.32)	1976 (6.33)	77 (5.98)	
Alcohol (%)	Never	819 (2.52)	797 (2.55)	22 (1.71)	0.114
	Previous	715 (2.20)	682 (2.19)	33(2.56)	
	Current	30,966 (95.28)	29,733 (95.26)	1233(95.73)	
DASH (%)	No	12,089 (38.58)	11,599 (38.54)	490 (39.68)	0.438
	Yes	19,243 (61.42)	18,498 (61.46)	745 (60.32)	
PRS		0.09 (0.91)	0.07 (0.90)	0.52 (0.96)	< 0.001
History of diabetesmellitus (%)	No	31,602 (97.24)	30,380 (97.33)	1222 (94.88)	< 0.001
	Yes	898 (2.76)	832 (2.67)	66 (5.12)	
History of hypertension (%)	No	25,390 (78.12)	24,625(78.90)	765(59.39)	< 0.001
	Yes	7110 (21.88)	6587 (21.10)	523 (40.61)	
History of other CVD (%)	No	31,333 (96.41)	30,175 (96.68)	1158 (89.91)	< 0.001
	Yes	1167 (3.59)	1037 (3.32)	130 (10.09)	
History of cancer (%)	No	30,355 (93.40)	29,168(93.45)	1187 (92.16)	0.076
	Yes	2145 (6.60)	2044 (6.55)	101 (7.84)	

*BMI *Body mass index, *DASH *Dietary approaches to stop hypertension, *PRS *Polygenic risk score, *CVD *Cardiovascular diseases

Participants who developed AF had significantly higher levels of all TyG, and their obesity-related derivatives, and larger left ventricular and atrial volumes compared with non-AF participants (Table S2). When stratified by TyG and its derivatives (Tables S3-S6), participants in higher quartiles had greater biatrial and biventricular volumes, and lower EFs than others. These trends were most pronounced for TyG-WC and TyG-BMI. Participants in the highest quartiles were also more likely to have hypertension, diabetes, and obesity.

### Associations with cardiac structure and function

**Fig. 1:**
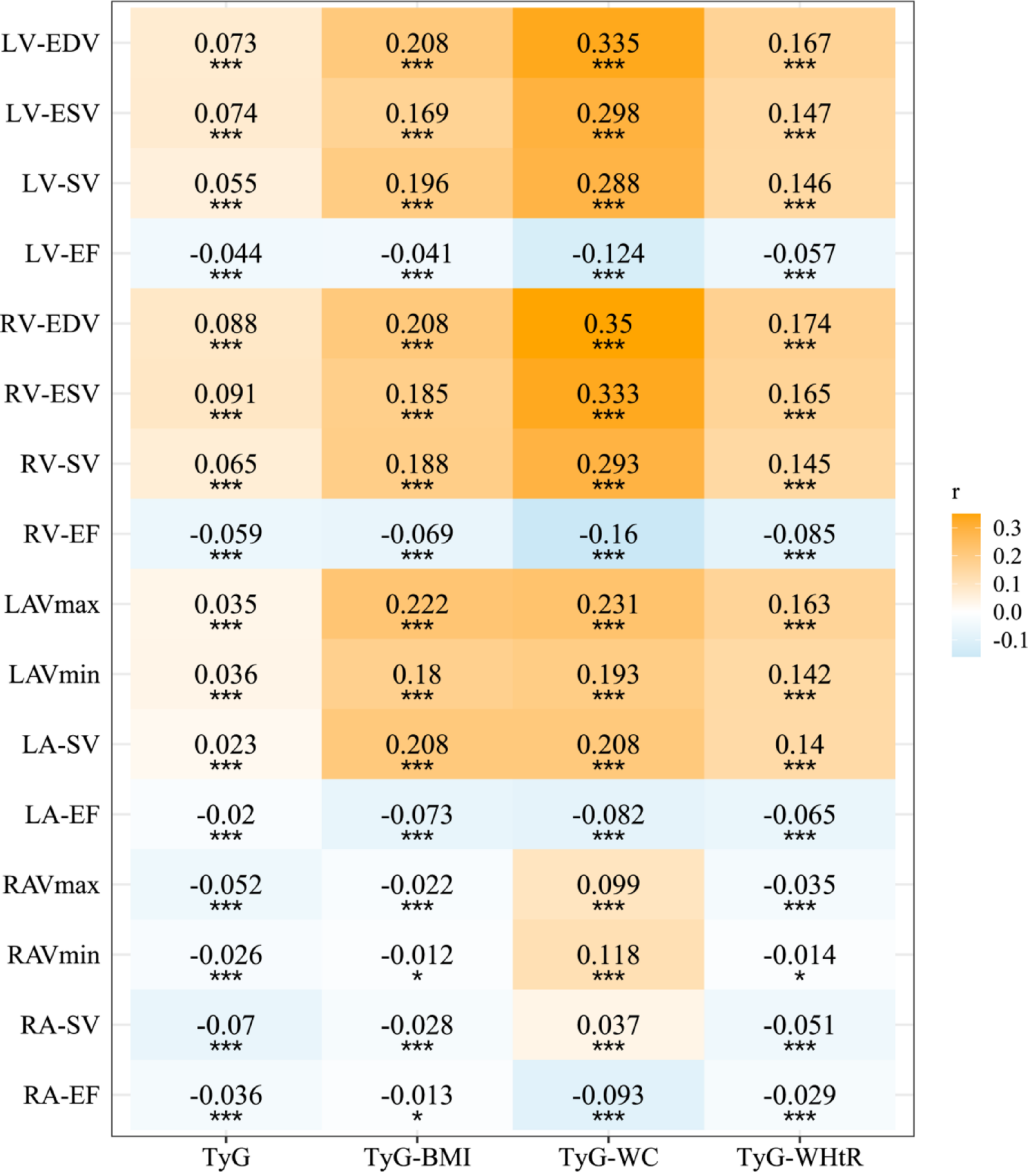
Correlation between TyG obesity-related derivatives and CMR indices. *CMR* Cardiac magnetic resonance imaging, *LV-EDV* Left ventricular end diastolic volume, *LV-ESV* Left ventricular end systolic volume, *LV-SV* Left ventricular stroke volume, *LV-EF* Left ventricular ejection fraction, *RV-EDV* Right ventricular end diastolic volume, *RV-ESV* Right ventricular end systolic volume, *RV-SV* Right ventricular stroke volume, *RV-EF* Right ventricular ejection fraction, *LAVmax* Left atrial maximum volume, *LAVmin* Left atrial minimum volume, *LA-SV* Left atrial stroke volume, *LA-EF* Left atrial ejection fraction, *RAVmax* Right atrial maximum volume, *RAVmin* Right atrial minimum volume, *RA-SV* Right atrial stroke volume, *RA-EF* Right atrial ejection fraction, *TyG* Triglyceride-glucose index, *TyG-BMI* TyG-body mass index, *TyG-WC* TyG-waist circumference, *TyG-WHtR* TyG-waist-to-height ratio

**Fig. 2:**
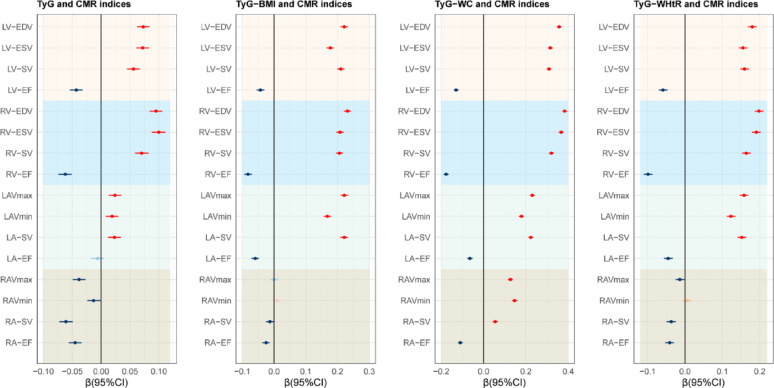
Associations between TyG obesity-related derivatives and CMR indices. Models were adjusted by age, sex, race, education, income, physical activity, smoke, alcohol, DASH, PRS, and history of diabetes mellitus, hypertension, other CVD, and cancer. *DASH* Dietary approaches to stop hypertension, *PRS* Polygenic risk score, *CVD* Cardiovascular diseases, *CMR* Cardiac magnetic resonance imaging, *LV-EDV* Left ventricular end diastolic volume, *LV-ESV* Left ventricular end systolic volume, *LV-SV* Left ventricular stroke volume, *LV-EF* Left ventricular ejection fraction, *RV-EDV* Right ventricular end diastolic volume, *RV-ESV* Right ventricular end systolic volume, *RV-SV* Right ventricular stroke volume, *RV-EF* Right ventricular ejection fraction, *LAVmax* Left atrial maximum volume, *LAVmin* Left atrial minimum volume, *LA-SV* Left atrial stroke volume, *LA-EF* Left atrial ejection fraction, *RAVmax* Rsight atrial maximum volume, *RAVmin* Right atrial minimum volume, *RA-SV* Right atrial stroke volume, *RA-EF* Right atrial ejection fraction, *TyG* Triglyceride-glucose index, *TyG-BMI* TyG-body mass index, *TyG-WC* TyG-waist circumference, *TyG-WHtR* TyG-waist-to-height ratio

Spearman correlation analysis revealed that TyG and its derivatives were positively correlated with ventricular and LA volumes, and negatively correlated with EFs across cardiac chambers (Fig. 1). Multivariable linear regression confirmed these associations after comprehensive covariate adjustment (Fig. 2, Tables S7-S10). In fully adjusted models, each 1-SD increase in TyG index was associated with increased LV-EDV (β = 0.073, 95% CI 0.062–0.084), RV-EDV (β = 0.095, 95% CI 0.084–0.106), and LAVmax (β = 0.024, 95% CI 0.013–0.035), and lower ventricular EFs (all *P* < 0.001). TyG obesity-related derivatives demonstrated substantially larger effect sizes. TyG-WC showed the strongest associations, with an increase in LV-EDV (β = 0.357, 95% CI 0.346–0.367), RV-EDV (β = 0.383, 95% CI 0.372–0.394), LAVmax(β = 0.23, 95% CI 0.218–0.241), and RAVmax (β = 0.127, 95% CI 0.115–0.138), and reduced in LV-EF (β = −0.130), RV-EF (β = −0.177), LA-EF (β = −0.065), and RA-EF (β = −0.11) (all *P* < 0.001).

### Cardiac parameters and AF

Increasing chamber volumes and decreasing EFs were significantly associated with incident AF (Table 2). In fully adjusted models, LAVmax (HR = 1.714, 95% CI 1.653–1.778) and LAVmin (HR = 1.551, 95% CI 1.520–1.582) were strongly associated with higher AF risk, whereas higher LA-EF was protective (HR = 0.629, 95% CI 0.592–0.669). Ventricular and RA volumes similarly demonstrated positive associations with AF risk. RCS analyses revealed significant non-linear associations for LAVmax, LAVmin, and LA-EF (*P*-nonlinear < 0.05; Figures S2-S5), with AF risk increasing exponentially at higher volumes and lower EFs.

**Table 2:** Associations between CMR indices and atrial fibrillation

CMR (per 1SD)	Model 1		Model 2		Model 3	
	HR (95%CI)	*P*	HR (95%CI)	*P*	HR (95%CI)	*P*
LV-EDV	1.326 (1.259, 1.398)	< 0.001	1.307 (1.235, 1.384)	< 0.001	1.296 (1.225, 1.371)	< 0.001
LV-ESV	1.289 (1.249, 1.329)	< 0.001	1.299 (1.255, 1.345)	< 0.001	1.279 (1.235, 1.325)	< 0.001
LV-SV	1.060 (0.999, 1.125)	0.054	1.035 (0.973, 1.100)	0.274	1.042 (0.980, 1.108)	0.187
LV-EF	0.725 (0.692,0.760)	< 0.001	0.730 (0.695, 0.766)	< 0.001	0.745 (0.709, 0.782)	< 0.001
RV-EDV	1.169 (1.094, 1.250)	< 0.001	1.136 (1.061, 1.217)	< 0.001	1.151 (1.075, 1.233)	< 0.001
RV-ESV	1.369 (1.288, 1.454)	< 0.001	1.339 (1.257, 1.426)	< 0.001	1.345 (1.263, 1.433)	< 0.001
RV-SV	0.938 (0.881, 0.997)	0.041	0.919 (0.863, 0.979)	0.009	0.932 (0.876, 0.993)	0.030
RV-EF	0.732 (0.698, 0.768)	< 0.001	0.741 (0.706, 0.777)	< 0.001	0.750 (0.715, 0.786)	< 0.001
LAVmax	1.755 (1.697, 1.816)	< 0.001	1.725 (1.665, 1.788)	< 0.001	1.714 (1.653, 1.778)	< 0.001
LAVmin	1.584 (1.554, 1.615)	< 0.001	1.554 (1.523, 1.585)	< 0.001	1.551 (1.520, 1.582)	< 0.001
LA-SV	0.990 (0.937, 1.045)	0.708	0.961 (0.908, 1.016)	0.163	0.958 (0.906, 1.014)	0.139
LA-EF	0.437 (0.421, 0.454)	< 0.001	0.454 (0.437, 0.473)	< 0.001	0.458 (0.440, 0.476)	< 0.001
RAVmax	1.517 (1.450, 1.586)	< 0.001	1.492 (1.425, 1.562)	< 0.001	1.508 (1.440, 1.578)	< 0.001
RAVmin	1.646 (1.585, 1.708)	< 0.001	1.614 (1.553, 1.677)	< 0.001	1.615 (1.555, 1.678)	< 0.001
RA-SV	1.037 (0.983, 1.094)	0.180	1.028 (0.974, 1.085)	0.321	1.042 (0.987, 1.100)	0.134
RA-EF	0.605 (0.569, 0.643)	< 0.001	0.624 (0.587, 0.663)	< 0.001	0.629 (0.592, 0.669)	< 0.001

Model 1 was adjusted by age, sex, race, education, and income

Model 2 was adjusted by Model 1 + physical activity, smoke, alcohol, DASH, and PRS

Model 3 was adjusted by Model 2 + history of diabetes mellitus, hypertension, other CVD, and cancer

*DASH* Dietary approaches to stop hypertension, *PRS* Polygenic risk score, *CVD* Cardiovascular diseases, *CMR* Cardiac magnetic resonance imaging, *LV-EDV* Left ventricular end diastolic volume, *LV-ESV* Left ventricular end systolic volume, *LV-SV* Left ventricular stroke volume, *LV-EF* Left ventricular ejection fraction, *RV-EDV* Right ventricular end diastolic volume, *RV-ESV* Right ventricular end systolic volume, *RV-SV* Right ventricular stroke volume, *RV-EF* Right ventricular ejection fraction, *LAVmax* Left atrial maximum volume, *LAVmin* Left atrial minimum volume, *LA-SV* Left atrial stroke volume, *LA-EF* Left atrial ejection fraction, *RAVmax* Right atrial maximum volume, *RAVmin* Right atrial minimum volume, *RA-SV* Right atrial stroke volume, *RA-EF* Right atrial ejection fraction

### Associations between TyG obesity-related derivatives and atrial fibrillation

**Fig. 3:**
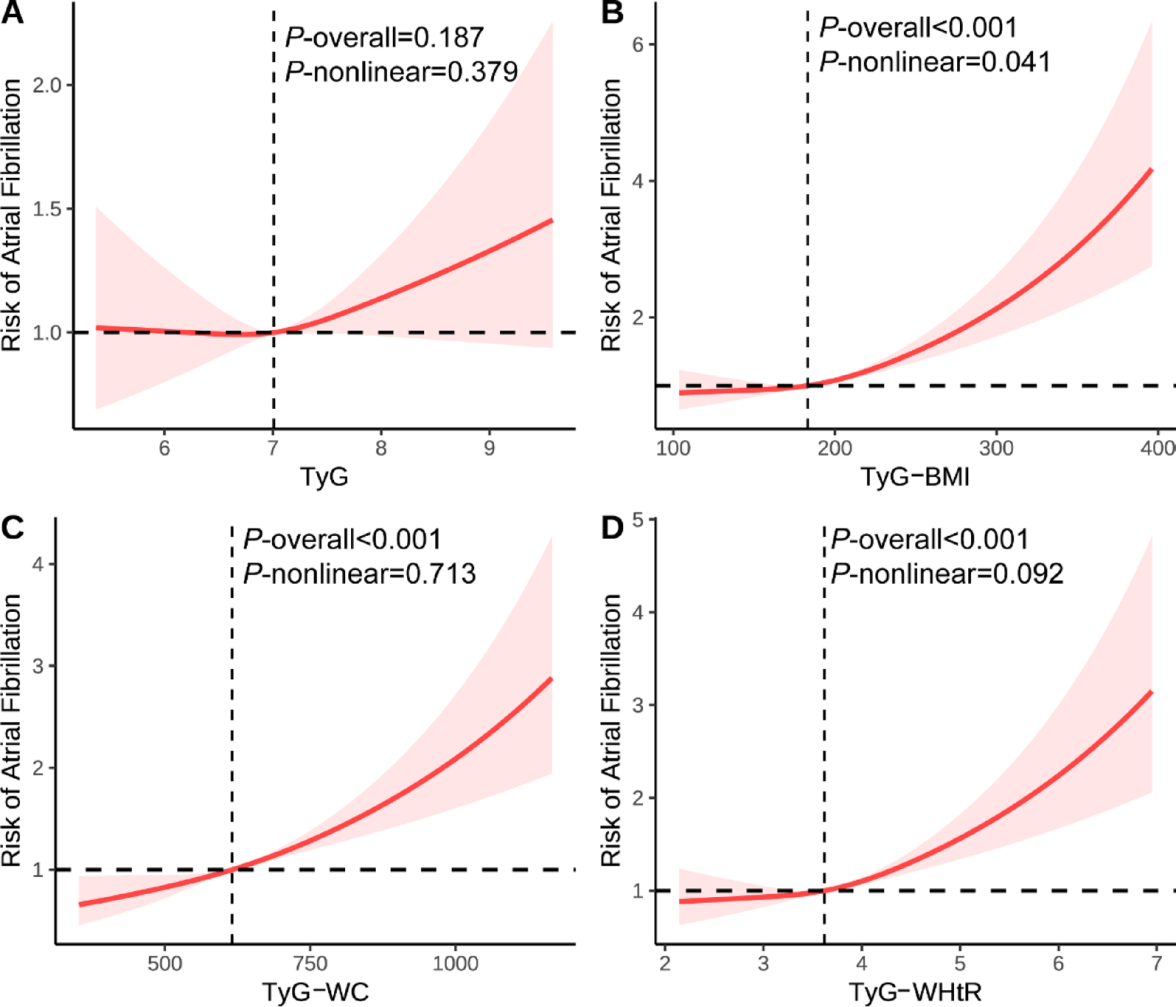
RCS analysis between TyG obesity-related derivatives and atrial fibrillation. Models were adjusted by age, sex, race, education, income, physical activity, smoke, alcohol, DASH, PRS, and history of diabetes mellitus, hypertension, other CVD, and cancer. *DASH* Dietary approaches to stop hypertension, *PRS* Polygenic risk score, *CVD* Cardiovascular diseases, *TyG* Triglyceride-glucose index, *TyG-BMI* TyG-body mass index, *TyG-WC* TyG-waist circumference, *TyG-WHtR* TyG-waist-to-height ratio

All TyG obesity-related derivatives (TyG-WC, TyG-BMI, and TyG-WHtR) demonstrated significant positive associations with AF risk, with TyG-WC exhibiting the most robust association (HR = 1.245, 95% CI 1.169–1.325), followed by TyG-BMI (HR = 1.223, 95% CI 1.158–1.293) and TyG-WHtR (HR = 1.190, 95% CI 1.122–1.262) (Table 3). In contrast, the TyG index alone was not independently associated with AF (HR = 1.048, 95% CI 0.989–1.110). Obesity indicators alone (BMI, WC, and WHtR) demonstrated smaller standardized effect sizes than TyG obesity-related derivatives (TyG-BMI, TyG-WC, and TyG-WHtR) (Table S11). RCS analyses revealed linear relationships between TyG-WC and TyG-WHtR with AF risk (*P*-nonlinear > 0.05), whereas TyG-BMI demonstrated a significant nonlinear pattern (*P*-nonlinear = 0.041; Fig. 3). Interaction analyses revealed significant interactions of age, sex, and race with TyG (all *P*-interaction < 0.05). Subgroup analyses further demonstrated significant associations between TyG and AF among participants aged < 65 years and female participants (all *P* < 0.05). Although no statistically significant interaction was observed for smoking status, a significant TyG-AF association was detected in previous smokers (*P* < 0.05). The associations between TyG, obesity-related derivatives, and incident AF were generally consistent across various demographic, lifestyle, and clinical subgroups (Figures S6-S9).

**Table 3:** Associations between TyG obesity-related derivatives and atrial fibrillation

TyG obesity-related derivatives	Model 1		Model 2		Model 3	
	HR (95%CI)	*P*	HR (95%CI)	*P*	HR (95%CI)	*P*
TyG (per 1SD)	1.062 (1.004, 1.122)	0.035	1.060 (1.001, 1.123)	0.045	1.048 (0.989, 1.110)	0.113
Q1	Ref		Ref		Ref	
Q2	1.020 (0.863, 1.207)	0.814	1.003 (0.845, 1.191)	0.970	1.006 (0.847, 1.194)	0.948
Q3	1.055 (0.895, 1.243)	0.523	1.047 (0.885, 1.237)	0.594	1.037 (0.877, 1.226)	0.669
Q4	1.113 (0.947, 1.309)	0.194	1.107 (0.938, 1.306)	0.229	1.089 (0.922, 1.286)	0.315
*P* for trend		0.161		0.175		0.266
TyG-BMI (per 1SD)	1.252 (1.189, 1.318)	< 0.001	1.242 (1.177, 1.311)	< 0.001	1.223 (1.158, 1.293)	< 0.001
Q1	Ref		Ref		Ref	
Q2	1.077 (0.899, 1.290)	0.419	1.056 (0.878, 1.269)	0.562	1.044 (0.868, 1.255)	0.647
Q3	1.192 (1.000, 1.421)	0.050	1.163 (0.972, 1.392)	0.099	1.139 (0.952, 1.363)	0.154
Q4	1.661 (1.405, 1.963)	< 0.001	1.628 (1.371, 1.933)	< 0.001	1.568 (1.318, 1.864)	< 0.001
*P* for trend		< 0.001		< 0.001		< 0.001
TyG-WC (per 1SD)	1.281 (1.209, 1.358)	< 0.001	1.266 (1.191, 1.346)	< 0.001	1.245 (1.169, 1.325)	< 0.001
Q1	Ref		Ref		Ref	
Q2	1.320 (1.083, 1.609)	0.006	1.307 (1.068, 1.599)	0.009	1.286 (1.050, 1.573)	0.015
Q3	1.365 (1.117, 1.669)	0.002	1.346 (1.096, 1.653)	0.005	1.321 (1.075, 1.622)	0.008
Q4	1.889 (1.550, 2.302)	< 0.001	1.844 (1.504, 2.262)	< 0.001	1.767 (1.439, 2.170)	< 0.001
*P* for trend		< 0.001		< 0.001		< 0.001
TyG-WHtR (per 1SD)	1.223 (1.158, 1.292)	< 0.001	1.213 (1.145, 1.285)	< 0.001	1.190 (1.122, 1.262)	< 0.001
Q1	Ref		Ref		ref	
Q2	1.056 (0.878, 1.271)	0.563	1.042 (0.864, 1.258)	0.666	1.026 (0.850, 1.238)	0.793
Q3	1.218 (1.017, 1.457)	0.032	1.188 (0.989, 1.429)	0.066	1.162 (0.966, 1.397)	0.111
Q4	1.508 (1.266, 1.795)	< 0.001	1.474 (1.231, 1.766)	< 0.001	1.407 (1.172, 1.687)	< 0.001
*P* for trend		< 0.001		< 0.001		< 0.001

Model 1 was adjusted by age, sex, race, education, and income

Model 2 was adjusted by Model 1 + physical activity, smoke, alcohol, DASH, and PRS

Model 3 was adjusted by Model 2 + history of diabetes mellitus, hypertension, other CVD, and cancer

*DASH* Dietary approaches to stop hypertension, *PRS* Polygenic risk score, *CVD* Cardiovascular diseases, *TyG* Triglyceride-glucose index, TyG-BMI TyG-body mass index, *TyG-WC* TyG-waist circumference, *TyG-WHtR* TyG-waist-to-height ratio

### Mediation of cardiac structure and function in the relationship between TyG obesity-related derivatives, and AF

**Fig. 4:**
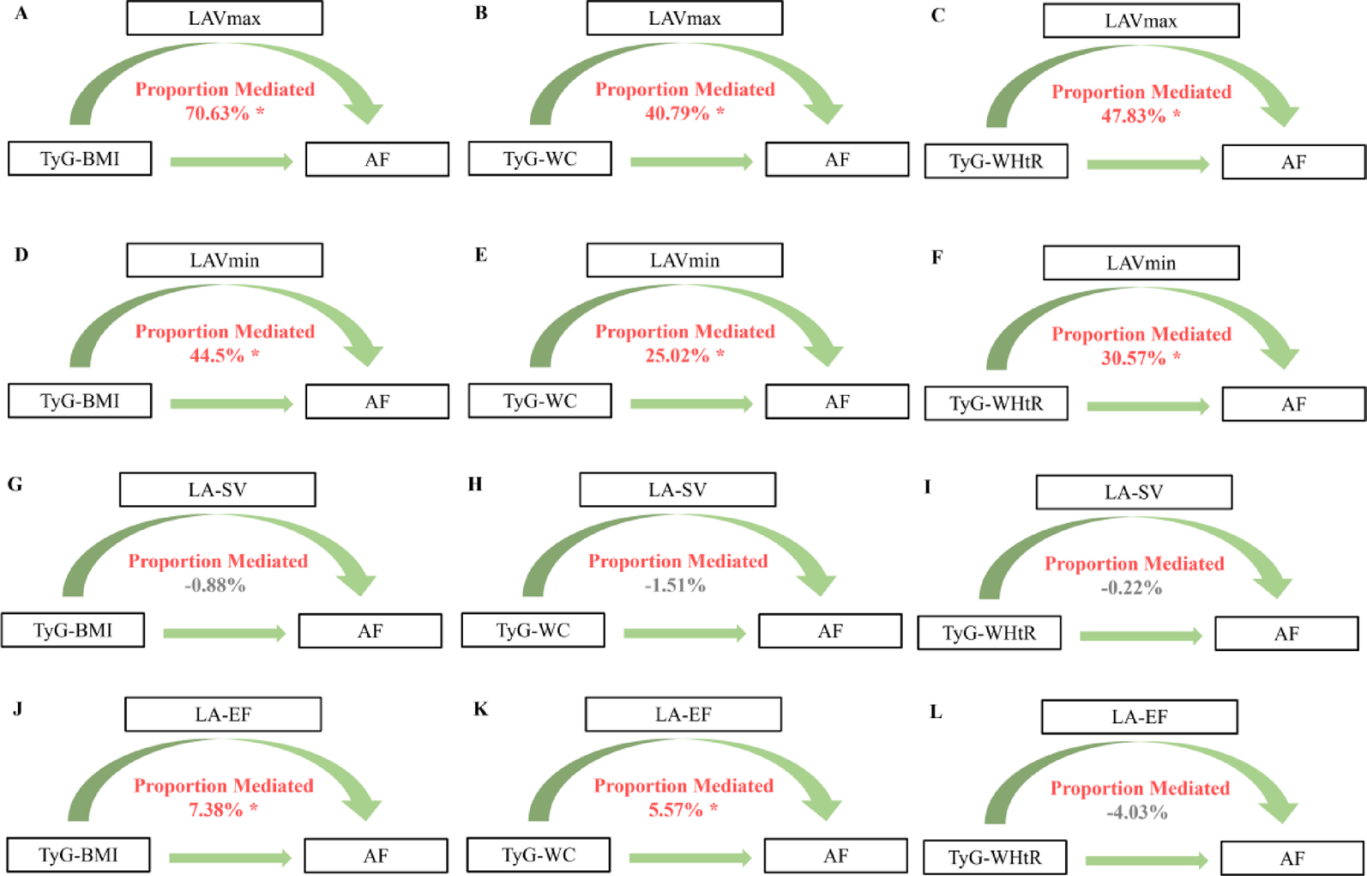
Mediation proportion of LA structure and function between TyG- obesity-related derivatives and atrial fibrillation. Models were adjusted by age, sex, race, education, income, physical activity, smoking, alcohol, DASH, PRS, and history of diabetes mellitus, hypertension, other CVD, and cancer. *DASH* Dietary approaches to stop hypertension, *PRS* Polygenic risk score, *CVD* Cardiovascular diseases, LAVmax Left atrial maximum volume, *LAVmin* Left atrial minimum volume, *LA-SV* Left atrial stroke volume, *LA-EF* Left atrial ejection fraction, *TyG* Triglyceride-glucose index, *TyG-BMI* TyG-body mass index, *TyG-WC* TyG-waist circumference, *TyG-WHtR* TyG-waist-to-height ratio, *AF* atrial fibrillation

Mediation analyses revealed that LA volumetric parameters, rather than functional parameters, primarily mediated the associations between TyG obesity-related derivatives and incident AF (Fig. 4 and Table S12). LAVmax emerged as the strongest mediator, accounting for 70.63%, 40.79%, and 47.83% of the associations for TyG-BMI, TyG-WC, and TyG-WHtR, respectively (all *P* < 0.001). LAVmin also demonstrated significant mediating effects ranging from 25.02% to 44.50%. While LA-EF only showed modest mediating effects for TyG-BMI (7.38%) and TyG-WC (5.57%).

Similarly, LV structural and functional parameters also demonstrated significant mediation effects (Figure S10 and Table S13). LV-EDV exhibited the strongest mediating role, accounting for 49.90%, 41.52%, and 41.30% of the associations for TyG-BMI, TyG-WC, and TyG-WHtR, respectively (all *P* < 0.001). LV-ESV demonstrated comparable mediating proportions ranging from 32.99% to 37.70%. Notably, unlike LA functional parameters, both LV-SV (15.87%-22.00%) and LV-EF (15.74%-19.20%) significantly mediated all TyG-AF associations (all *P* < 0.001).

RA parameters also exhibited mediation in the TyG-AF associations (Figure S11 and Table S14). RA-EF accounted for 13.62%, 23.94%, and 20.13% of the associations for TyG-BMI, TyG-WC, and TyG-WHtR, respectively (all *P* < 0.001). RAVmax and RAVmin demonstrated significant mediating effects primarily for TyG-WC (27.51% and 34.39%, respectively) and TyG-BMI (8.37% and 12.69%, respectively), with weaker effects for TyG-WHtR.

RV parameters also significantly mediated the TyG-AF associations (Figure S12 and Table S15). RV-ESV exhibited the strongest mediating role, accounting for 57.13%, 51.47%, and 49.80% of the associations for TyG-BMI, TyG-WHtR, and TyG-WC, respectively (all *P* < 0.001). RV-EDV demonstrated comparable mediating proportions (36.91%-45.70%), while RV-EF showed moderate effects (23.37–24.67%). RV-SV contributed the smallest mediating proportions (7.17–12.36%).

### Sensitivity analysis

Several sensitivity analyses were conducted to assess the robustness of our findings (Tables S16-S18). When excluding participants with missing covariate data, the associations between TyG obesity-related derivatives and incident AF remained significant: TyG-WC (HR = 1.246, 95% CI 1.160–1.340), TyG-BMI (HR = 1.223, 95% CI 1.147–1.304), and TyG-WHtR (HR = 1.202, 95% CI 1.123–1.287). After excluding participants who developed AF within the first two years of follow-up, results were consistent with the primary analysis, with TyG-WC showing the strongest association (HR = 1.245, 95% CI 1.167–1.327). Similarly, excluding participants with a history of CVD yielded comparable results, with all TyG obesity-related derivatives maintaining significant positive associations with AF risk (all *P* < 0.001).

The mediating effects of cardiac structure and function also remained robust across all sensitivity analyses (Tables S19-S21). Results remained consistent after excluding participants with missing data, early AF events, or prevalent CVD at baseline. The mediating role of LAVmax remained robust across all sensitivity analyses.

## Discussion

We found that the TyG derivatives were significantly associated with adverse cardiac remodeling and dysfunction, and the risk of AF, and that these associations were stronger when TyG was combined with measures of body size, particularly waist circumference. Furthermore, we demonstrated that cardiac structural remodeling, especially LA volumetric enlargement, substantially mediated the pathway from TyG to obesity-related derivatives to AF development. These findings highlight the key role of cardiac remodeling in linking metabolic disturbances to AF pathogenesis.

The relationship between the TyG index and AF has yielded inconsistent findings across previous studies. While Azarboo et al. [24] reported that the TyG index was higher in patients with AF than in non-AF counterparts through a meta-analysis. Similarly, Chen et al. [25] demonstrated that elevated TyG was an independent risk factor for AF only among non-diabetic hospitalized patients, whereas Shi et al. [14] identified a U-shaped rather than a linear relationship in the UK Biobank population. Our study found that TyG levels were not independently associated with AF, but this association was observed in subgroups including individuals younger than 65 years, females, and former smokers. Importantly, our study extends prior work by showing that TyG obesity-related derivatives, which integrate both insulin resistance and adiposity measures, exhibit superior and more consistent associations with AF risk than TyG alone. Regarding the association between TyG and cardiac remodeling, prior CMR studies have primarily focused on left ventricular parameters. Jiang et al. [16] linked a higher TyG index with impaired LV global function and adverse remodeling in patients with diabetes, while Velagaleti et al. [10] demonstrated associations between insulin resistance and concentric LV remodeling in the Framingham cohort. Our study expands upon these findings by providing a comprehensive four-chamber evaluation, and demonstrating that TyG obesity-related derivatives are associated with structural and functional alterations across both atrial and ventricular chambers, thereby offering a more integrated understanding of metabolic-cardiac interactions.

Significant interactions were observed for age and sex, with the association between TyG and AF evident specifically among individuals younger than 65 years and women. In older individuals, age-related structural remodeling, comorbidity burden, and competing traditional risk factors likely attenuate the detectable contribution of insulin resistance to AF risk [26]. In contrast, among individuals younger than 65 years, metabolic factors may represent a more dominant driver of atrial remodeling before other etiologies become dominant. Regarding sex-specific patterns, accumulating evidence suggests that metabolic dysregulation confers a relatively greater risk of AF in women than in men. A cohort demonstrated that the relative rates of incident AF attributable to diabetes were consistently higher in women than in men across all age groups [27]. Similarly, another study found that women under 55 years with type 2 diabetes had a more than twofold increased risk of AF compared with men, while the sex difference narrowed substantially with advancing age [28]. Women also exhibit greater degrees of atrial fibrosis than men with AF [29], suggesting a heightened susceptibility to insulin resistance–driven pro-fibrotic atrial remodeling that may amplify the TyG-AF association in this sex. Clinically, this finding suggests that elevated TyG levels in individuals younger than 65 years and women may warrant more intensive AF assessment and targeted prevention strategies.

The mechanistic pathways linking TyG derivatives to AF through cardiac remodeling are multifaceted and involve interrelated molecular cascades. At the molecular level, insulin resistance activates mitogen-activated protein kinase signaling, promoting oxidative stress, cardiac hypertrophy, fibrosis, and apoptosis [30]. Specifically, transforming growth factor-β1 is upregulated in insulin-resistant states, stimulating atrial fibroblast proliferation and extracellular matrix deposition, thereby creating a profibrotic substrate for AF [31]. Insulin resistance also disrupts intracellular calcium homeostasis through enhanced Ca²⁺/calmodulin-dependent protein kinase II activity, increasing sarcoplasmic reticulum calcium content and diastolic calcium leak, thereby enhancing triggered activity and AF susceptibility [32]. The obesity component captured by TyG derivatives contributes through distinct yet complementary mechanisms. Progressive weight gain induces LA enlargement, interstitial fibrosis, inflammatory infiltration, and lipidosis, resulting in decreased conduction velocity and increased conduction heterogeneity [33]. Furthermore, epicardial adipose tissue secretes proinflammatory cytokines and profibrotic adipokines, including interleukin-6, tumor necrosis factor-α, and activin A, that accelerate structural and electrical remodeling of the atria [34]. These pathological processes converge on the LA, which explains our finding that LA volumetric parameters predominantly mediated the TyG-AF association. Atrial stretch activates neurohormonal signaling cascades and promotes myocyte hypertrophy and interstitial fibrosis, collectively creating an electrophysiological substrate characterized by slow conduction, shortened refractoriness, and increased anisotropy, thereby favoring reentrant circuits and AF perpetuation [35, 36].

Beyond LA remodeling, the substantial mediating role of RV volumetric parameters warrants careful consideration. First, insulin resistance promotes pulmonary vascular remodeling through endothelial dysfunction and oxidative stress, elevating pulmonary arterial pressures and driving RV pressure overload [37, 38]. Second, obesity-related obstructive sleep apnea imposes recurrent hypoxia and intrathoracic pressure swings that further compound pulmonary vascular resistance and right heart burden [39]. Third, insulin resistance-induced left ventricular diastolic dysfunction elevates pulmonary venous pressures and propagates hemodynamic stress retrogradely to the right heart, suggesting that RV dilation may partly reflect downstream left-sided metabolic cardiomyopathy [36]. Finally, epicardial adipose tissue paracrine signaling driven by lipotoxicity and inflammation may directly impair RV function and promote right atrial remodeling, creating an additional arrhythmogenic substrate [40]. Collectively, these findings support the concept that the metabolic-AF pathway operates through integrated pan-cardiac remodeling, rather than isolated left atrial changes.

The present study carries important clinical implications. TyG derivatives are inexpensive and readily obtainable from routine laboratory and anthropometric measurements, making them practical tools for AF risk stratification because they do not require specialized equipment or invasive procedures [41]. The mechanistic insights provided by our mediation analysis suggest that interventions targeting metabolic dysfunction, particularly those aimed at reducing insulin resistance and preventing adverse cardiac remodeling, may attenuate AF susceptibility. Mounting evidence demonstrates that aggressive weight management and comprehensive risk factor control can reverse the natural progression of AF from paroxysmal to persistent forms, with weight loss exceeding 10% associated with regression to less severe AF phenotypes [41, 42]. Our identification of LA structural parameters as key mediators reinforces the rationale for early metabolic intervention before irreversible atrial remodeling occurs [43].

Several limitations should be acknowledged. First, the observational design precludes definitive causal inference, and residual confounding cannot be entirely excluded. Second, the mediation analysis relies on the assumptions of temporal ordering and the absence of unmeasured confounding; therefore, the mediation estimates should be interpreted as a statistical decomposition of association rather than proof of causal mechanisms. Third, although the TyG index serves as a practical and accessible surrogate marker for insulin resistance, it does not directly measure circulating insulin levels and may not fully capture metabolic heterogeneity. Fourth, TyG derivatives were assessed at a single time point, precluding evaluation of longitudinal trajectories. Fifth, AF ascertainment was based on administrative codes, and asymptomatic or paroxysmal AF cases may have been underdetected, potentially leading to non-differential outcome misclassification. Sixth, the UK Biobank cohort predominantly comprises White British participants, which may limit generalizability to more diverse populations. Finally, although TyG obesity-related derivatives demonstrated stronger associations than obesity measures alone, formal evaluation of incremental predictive performance was not performed and warrants further investigation.

## Conclusion

In conclusion, elevated TyG obesity-related derivatives were associated with adverse cardiac remodeling and AF risk, with TyG-WC demonstrating the strongest association with AF risk. CMR-derived markers of left atrial structure and function partially mediated these associations, linking metabolic dysfunction and adiposity to atrial arrhythmogenesis. These findings underscore the importance of metabolic assessment in AF risk stratification and support strategies targeting insulin resistance and visceral adiposity to mitigate cardiac remodeling and reduce AF susceptibility.

## Supplementary Information

Below is the link to the electronic supplementary material.

Supplementary Material 1.

## Data Availability

Data set: Available from the UK Biobank on request ( [www.ukbiobank.ac.uk](http:/www.ukbiobank.ac.uk) ).

## Abbreviations

TyG: Triglyceride-glucose
TyG-BMI: Triglyceride-glucose-body mass index
TyG-WC: Triglyceride-glucose-waist circumference
TyG-WHtR: Triglyceride-glucose-waist-to-height ratio
AF: Atrial fibrillation
CMR: Cardiac magnetic resonance
LA: Left atrium
LAVmax: Left atrial maximum volume
LAVmin: Left atrial minimum volume
LA-EF: Left atrial ejection fraction
LV: Left ventricle
LV-EDV: Left ventricular end-diastolic volume
LV-ESV: Left ventricular end-systolic volume
LV-SV: Left ventricular stroke volume
LV-EF: Left ventricular ejection fraction
RA: Right atrium
RAVmax: Right atrial maximum volume
RAVmin: Right atrial minimum volume
RA-EF: Right atrial ejection fraction
RV: Right ventricle
RV-EDV: Right ventricular end-diastolic volume
RV-ESV: Right ventricular end-systolic volume
RV-SV: Right ventricular stroke volume
RV-EF: Right ventricular ejection fraction
EF: Ejection fraction
HR: Hazard ratio
CI: Confidence interval
SD: Standard deviation
RCS: Restricted cubic spline
