# Supplementary material for: Associations of the triglyceride-glucose index and its obesity-related derivatives with cardiac structure, function, and incident atrial fibrillation: a prospective cohort study using cardiac magnetic resonance
Source: Cardiovasc Diabetol. 2026 Mar 31;25:151. doi: 10.1186/s12933-026-03143-x (PMC13162421; doi:10.1186/s12933-026-03143-x)
Supplement: Supplementary file 1 — Supplementary Material 1. [file 12933_2026_3143_MOESM1_ESM.doc]

**Associations of the Triglyceride-Glucose Index and Its Obesity-Related Derivatives with Cardiac Structure, Function, and Incident Atrial Fibrillation: A Prospective Cohort Study Using Cardiac Magnetic Resonance**

**Supplement**

[Figure S1. Flowchart of participant selection. 2](#__RefHeading___Toc11150)

[Figure S2. RCS analysis between LA structure, function and atrial fibrillation. 3](#__RefHeading___Toc15796)

[Figure S3. RCS analysis of the associations between LV structure, function and atrial fibrillation. 4](#__RefHeading___Toc28003)

[Figure S4. RCS analysis of the associations between RA structure, function and atrial fibrillation 5](#__RefHeading___Toc13283)

[Figure S5. RCS analysis of the associations between RV structure, function and atrial fibrillation. 6](#__RefHeading___Toc2028)

[Figure S6. Subgroup analysis of the association between TyG and atrial fibrillation. 7](#__RefHeading___Toc23713)

[Figure S7. Subgroup analysis of the association between TyG-BMI and atrial fibrillation. 8](#__RefHeading___Toc21959)

[Figure S8. Subgroup analysis of the association between TyG-WC and atrial fibrillation. 9](#__RefHeading___Toc1913)

[Figure S9. Subgroup analysis of the association between TyG-WHtR and atrial fibrillation. 10](#__RefHeading___Toc21031)

[Figure S10. Mediation proportions of LV structure and function in the association between TyG obesity-related derivatives and atrial fibrillation. 11](#__RefHeading___Toc27970)

[Figure S11. Mediation proportions of RA structure and function in the association between TyG obesity-related derivatives and atrial fibrillation. 12](#__RefHeading___Toc6963)

[Figure S12. Mediation proportions of RV structure and function in the association between TyG obesity-related derivatives and atrial fibrillation. 13](#__RefHeading___Toc391)

[Table S1. Diagnostic criteria for of atrial fibrillation. 14](#__RefHeading___Toc29765)

[Table S2. TyG obesity-related derivatives and CMR characteristics of participants with atrial fibrillation and the control group. 14](#__RefHeading___Toc2128)

[Table S3. Baseline characteristics of participants according to TyG quartiles. 15](#__RefHeading___Toc12043)

[Table S4. Baseline characteristics of participants according to TyG-BMI quartiles. 17](#__RefHeading___Toc23348)

[Table S5. Baseline characteristics of participants according to TyG-WC quartiles. 18](#__RefHeading___Toc23266)

[Table S6. Baseline characteristics of participants according to TyG-WHtR quartiles. 20](#__RefHeading___Toc24522)

[Table S7. Associations between TyG and CMR indices. 22](#__RefHeading___Toc21479)

[Table S8. Associations between TyG-BMI and CMR indices. 22](#__RefHeading___Toc772)

[Table S9. Associations between TyG-WC and CMR indices. 23](#__RefHeading___Toc22403)

[Table S10. Associations between TyG-WHtR and CMR indices. 24](#__RefHeading___Toc26611)

[Table S11. Associations between obesity indicators and atrial fibrillation 25](#__RefHeading___Toc12939)

[Table S12. Mediating roles of LA structure and function in the association between TyG obesity-related derivatives and atrial fibrillation. 26](#__RefHeading___Toc11772)

[Table S13. Mediating roles of LV structure and function in the association between TyG obesity-related derivatives and atrial fibrillation. 27](#__RefHeading___Toc32040)

[Table S14. Mediating roles of RA structure and function in the association between TyG obesity-related derivatives and atrial fibrillation. 28](#__RefHeading___Toc4506)

[Table S15. Mediating roles of RV structure and function in the association between TyG obesity-related derivatives and atrial fibrillation. 29](#__RefHeading___Toc15130)

[Table S16. Sensitivity analysis of associations between TyG obesity-related derivatives and atrial fibrillation after excluding participants with missing data. 30](#__RefHeading___Toc1032)

[Table S17. Sensitivity analysis of associations between TyG obesity-related derivatives and atrial fibrillation after excluding participants who developed atrial fibrillation within the first two-year follow-up period. 30](#__RefHeading___Toc11040)

[Table S18. Sensitivity analysis of associations between TyG obesity-related derivatives and atrial fibrillation after excluding participants with a history of CVD. 31](#__RefHeading___Toc31133)

[Table S19. Sensitivity analysis of mediating roles of cardiac structure and function in the association between TyG obesity-related derivatives and atrial fibrillation after excluding participants with missing data. 33](#__RefHeading___Toc9862)

[Table S20. Sensitivity analysis of mediating roles of cardiac structure and function in the association between TyG obesity-related derivatives and atrial fibrillation after excluding participants who developed atrial fibrillation within the first two-year follow-up period. 36](#__RefHeading___Toc27547)

[Table S21. Sensitivity analysis of mediating roles of cardiac structure and function in the association between TyG obesity-related derivatives and atrial fibrillation after excluding participants with a history of CVD. 39](#__RefHeading___Toc14881)

**
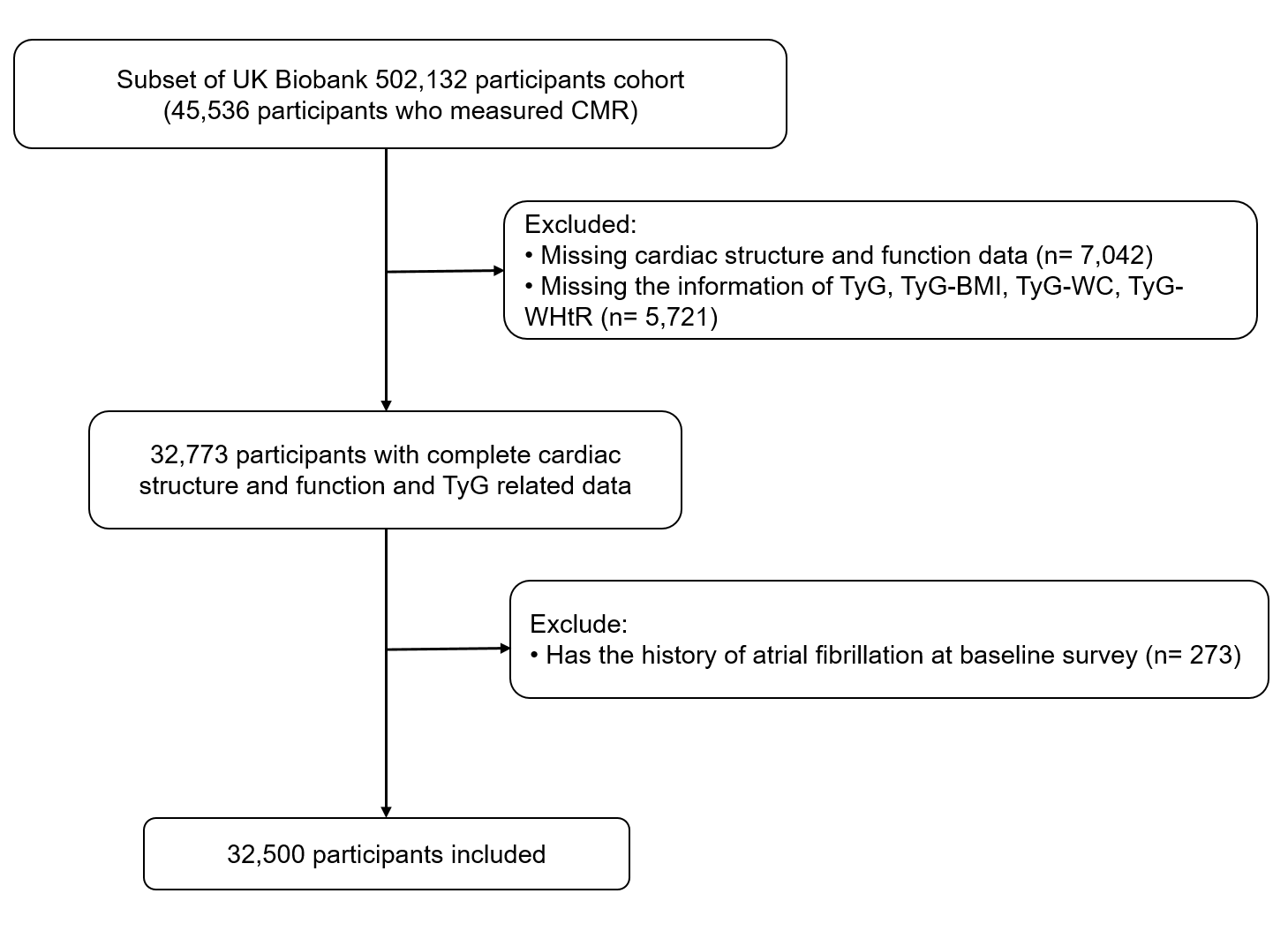
**

**Figure S1. Flowchart of participant selection.**

**
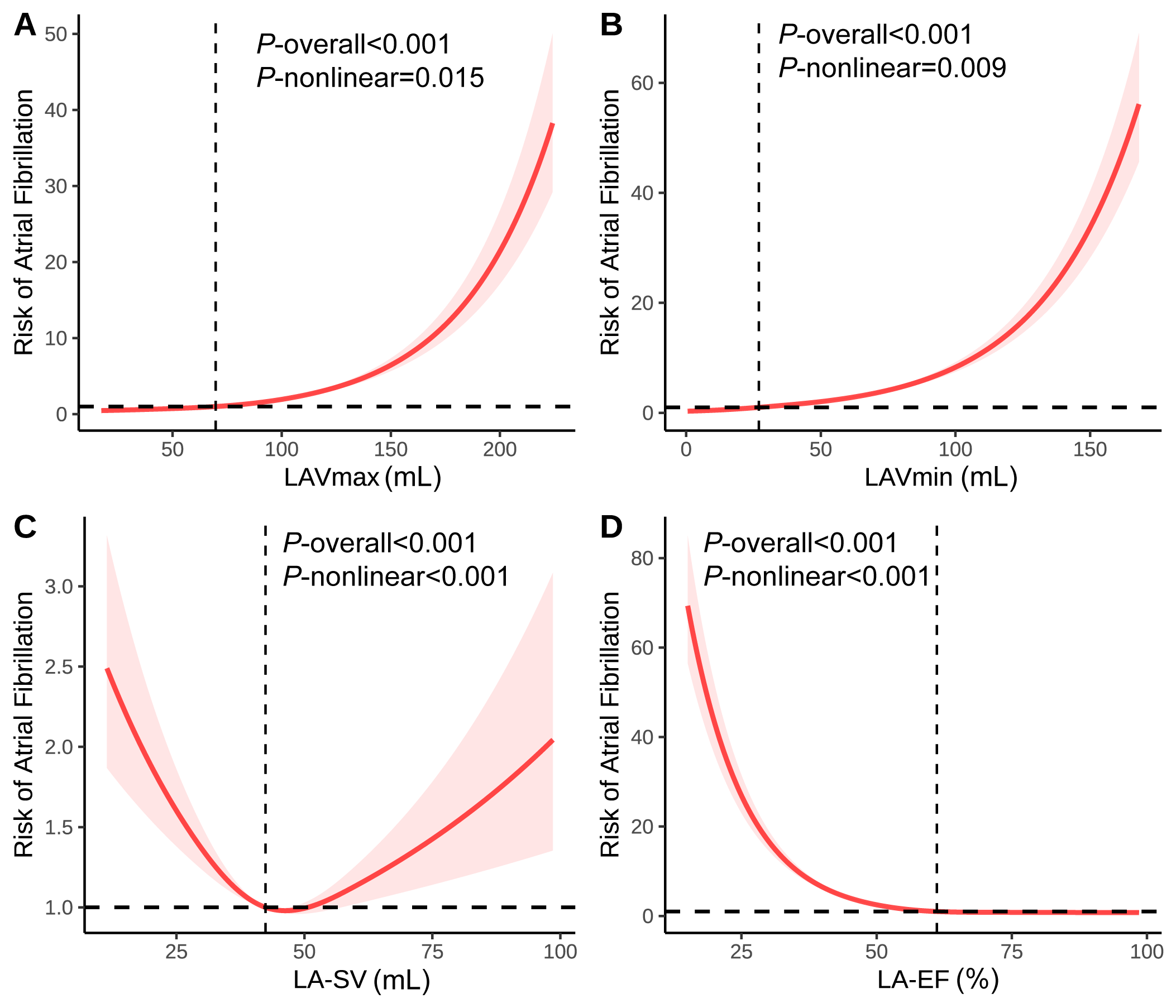
**

**Figure S2. RCS analysis between LA structure, function and atrial fibrillation.**

Models were adjusted by age, sex, race, education, income, physical activity, smoke, alcohol, DASH, PRS, and history of diabetes mellitus, hypertension, other CVD, and cancer. DASH: Dietary approaches to stop hypertension; PRS: Polygenic risk score; CVD: Cardiovascular diseases; LAVmax: Left atrial maximum volume; LAVmin: Left atrial minimum volume; LA-SV: Left atrial stroke volume; LA-EF: Left atrial ejection fraction; RCS: Restricted cubic spline.

**
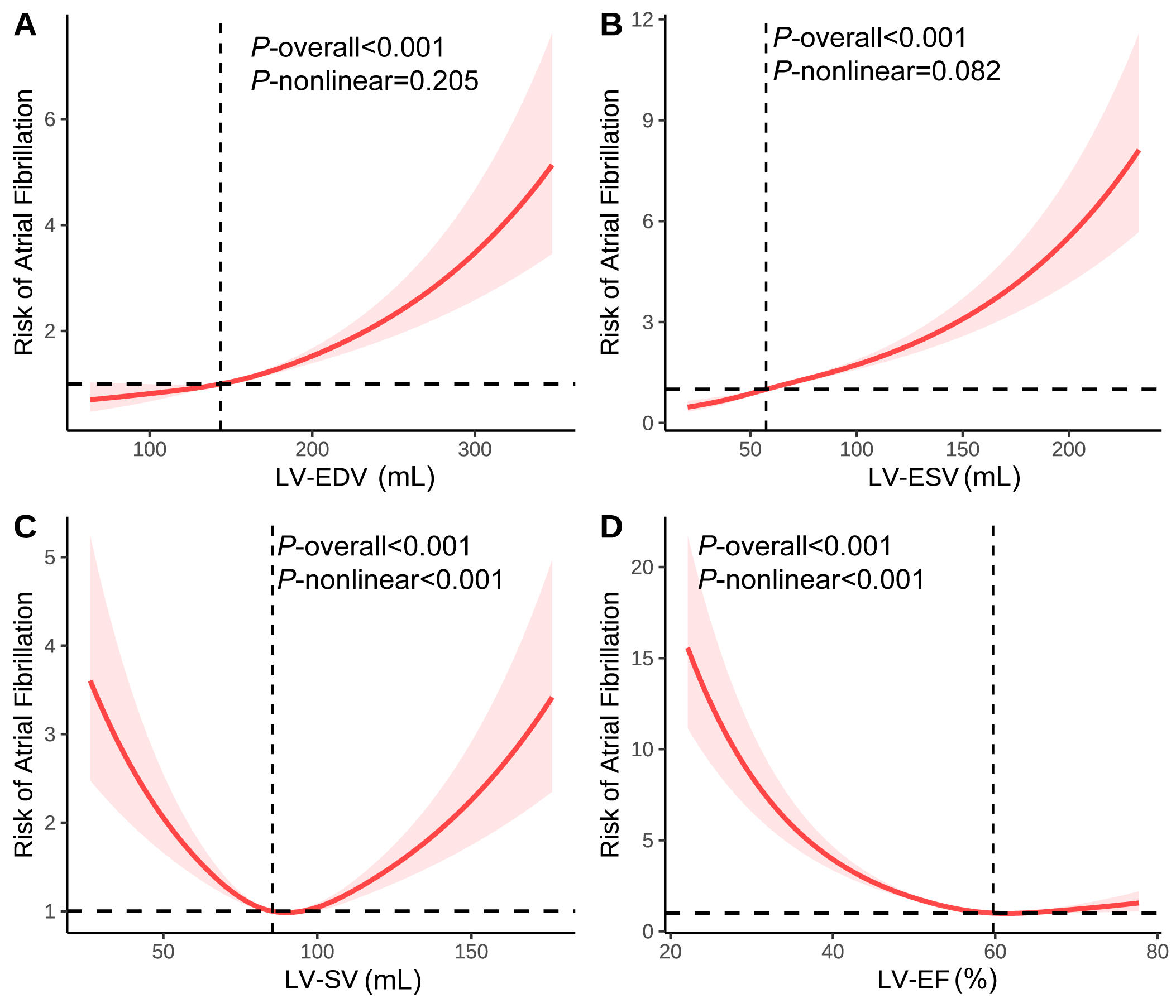
**

**Figure S3. RCS analysis of the associations between LV structure, function and atrial fibrillation.**

Models were adjusted by age, sex, race, education, income, physical activity, smoke, alcohol, DASH, PRS, and history of diabetes mellitus, hypertension, other CVD, and cancer. DASH: Dietary approaches to stop hypertension; PRS: Polygenic risk score; CVD: Cardiovascular diseases; LV-EDV: Left ventricular end diastolic volume; LV-ESV: Left ventricular end systolic volume; LV-SV: Left ventricular stroke volume; LV-EF: Left ventricular ejection fraction; RCS: Restricted cubic spline.

**
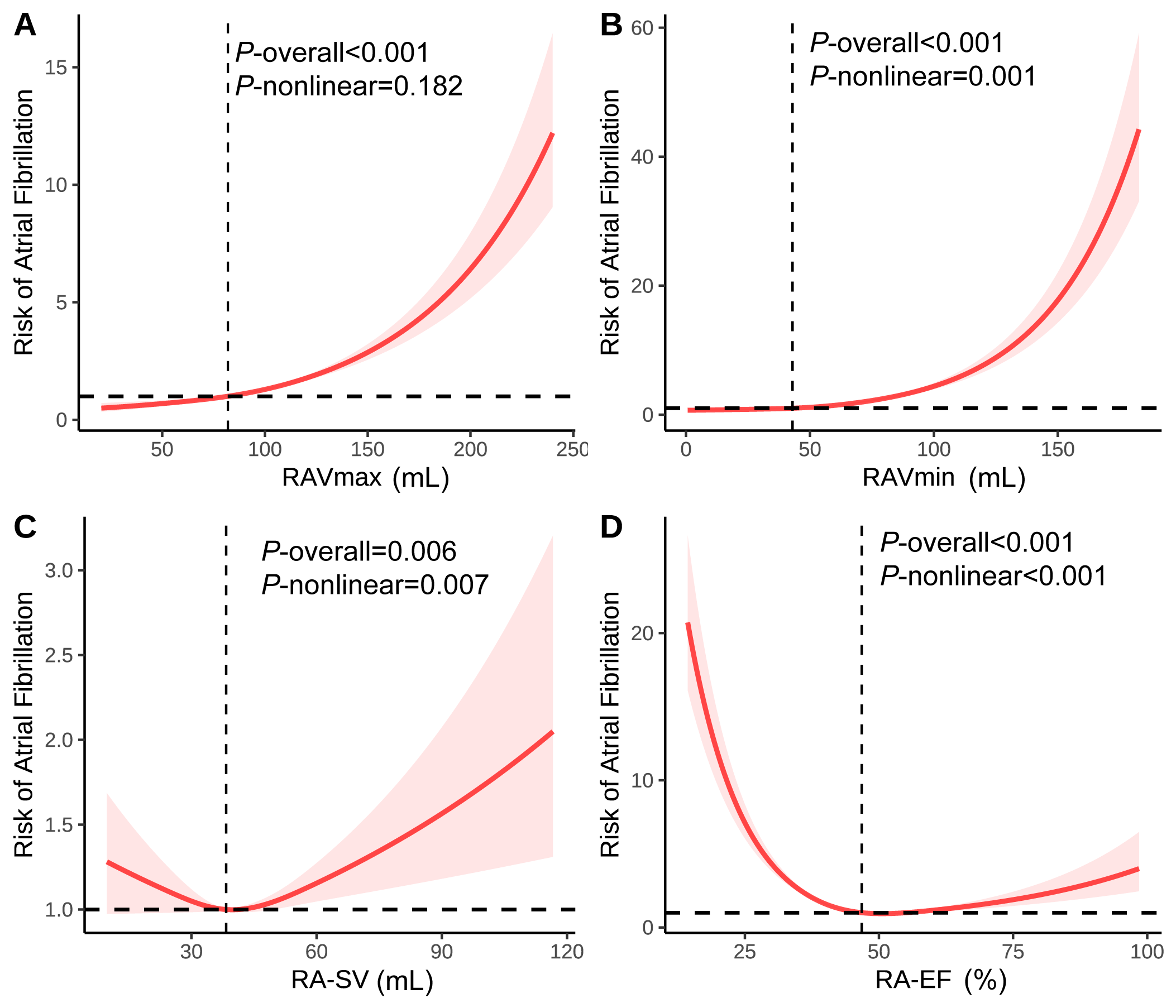
**

**Figure S4. RCS analysis of the associations between RA structure, function and atrial fibrillation**

Models were adjusted by age, sex, race, education, income, physical activity, smoke, alcohol, DASH, PRS, and history of diabetes mellitus, hypertension, other CVD, and cancer. DASH: Dietary approaches to stop hypertension; PRS: Polygenic risk score; CVD: Cardiovascular diseases; RAVmax: Right atrial maximum volume; RAVmin: Right atrial minimum volume; RA-SV: Right atrial stroke volume; RA-EF: Right atrial ejection fraction; RCS: Restricted cubic spline.

**
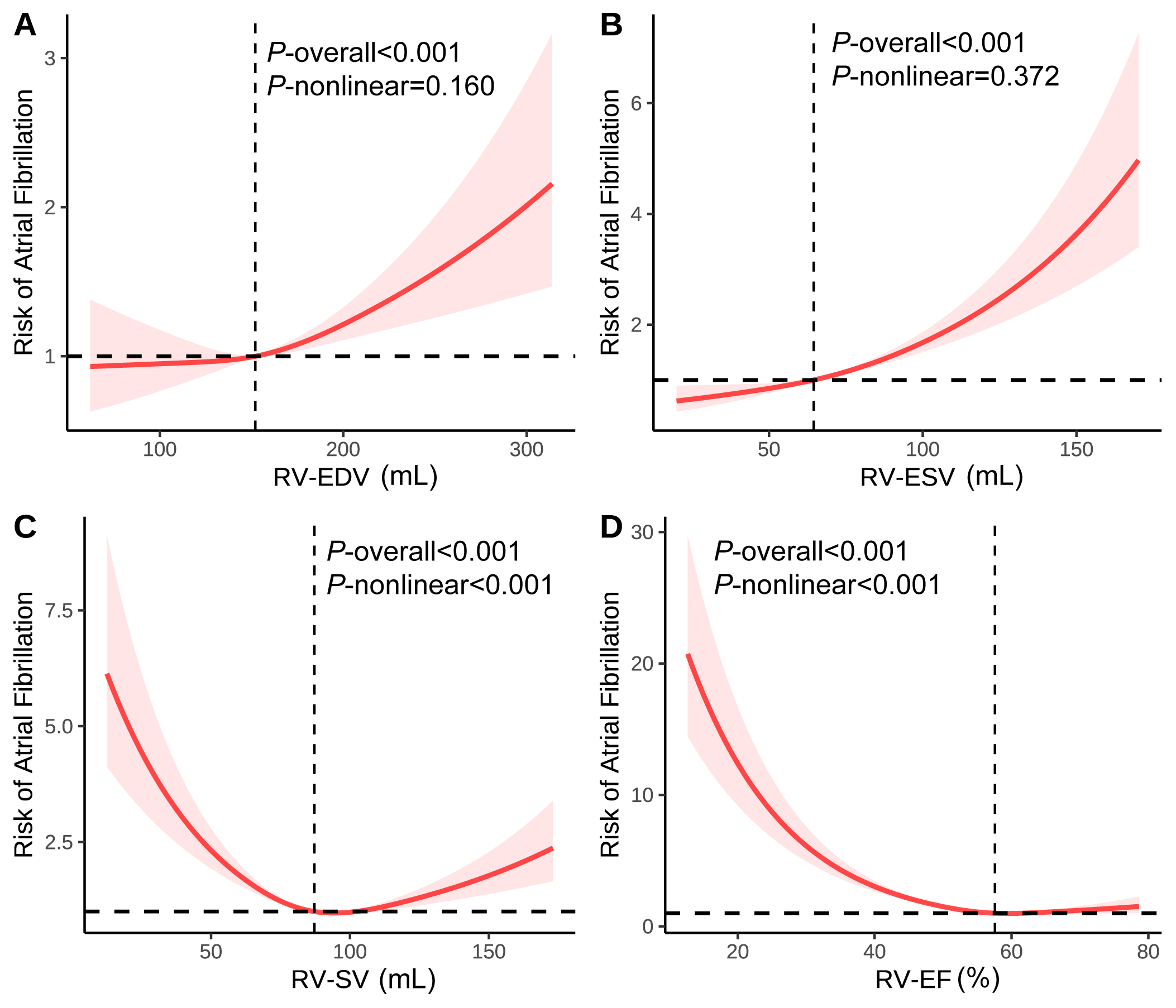
**

**Figure S5. RCS analysis of the associations between RV structure, function and atrial fibrillation.**

Models were adjusted by age, sex, race, education, income, physical activity, smoke, alcohol, DASH, PRS, and history of diabetes mellitus, hypertension, other CVD, and cancer. DASH: Dietary approaches to stop hypertension; PRS: Polygenic risk score; CVD: Cardiovascular diseases; RV-EDV: Right ventricular end diastolic volume; RV-ESV: Right ventricular end systolic volume; RV-SV: Right ventricular stroke volume; RV-EF: Right ventricular ejection fraction; RCS: Restricted cubic spline.

**
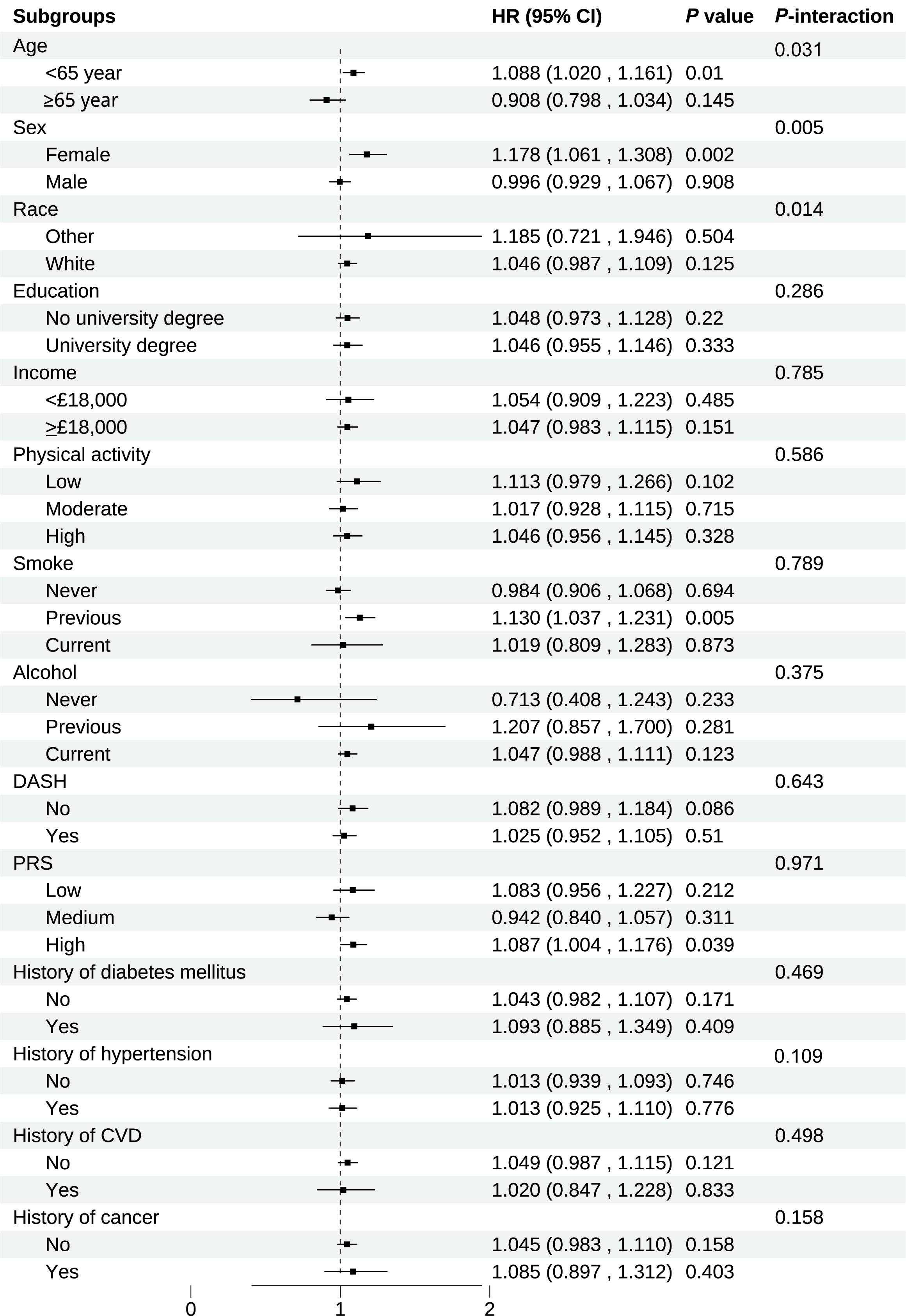
**

**Figure S6. Subgroup analysis of the association between TyG and atrial fibrillation.**

Models were adjusted by age, sex, race, education, income, physical activity, smoke, alcohol, DASH, PRS, and history of diabetes mellitus, hypertension, other CVD, and cancer. DASH: Dietary approaches to stop hypertension; PRS: Polygenic risk score; CVD: Cardiovascular diseases; TyG: Triglyceride-glucose index.

**
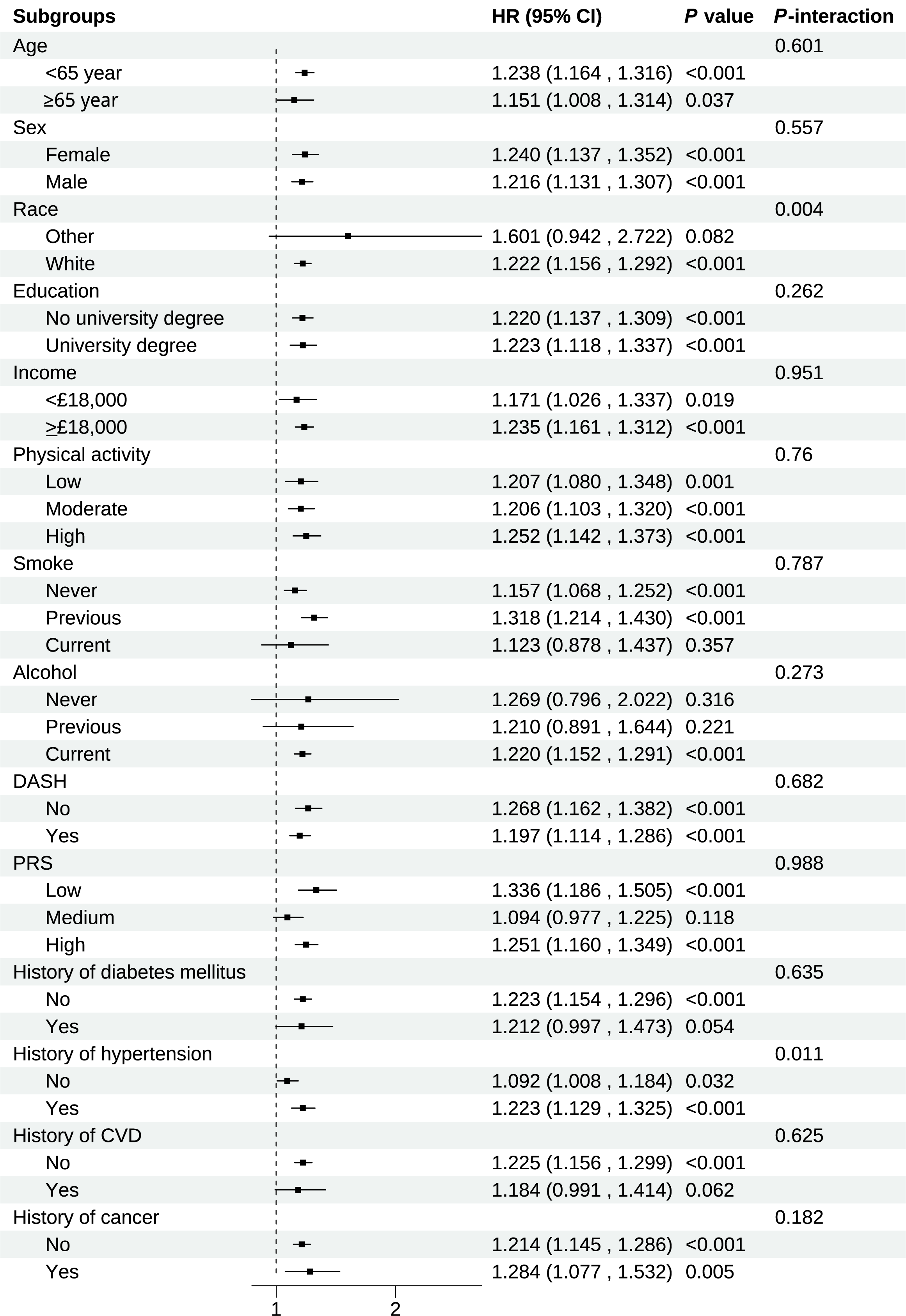
**

**Figure S7. Subgroup analysis of the association between TyG-BMI and atrial fibrillation.**

Models were adjusted by age, sex, race, education, income, physical activity, smoke, alcohol, DASH, PRS, and history of diabetes mellitus, hypertension, other CVD, and cancer. DASH: Dietary approaches to stop hypertension; PRS: Polygenic risk score; CVD: Cardiovascular diseases; TyG: Triglyceride-glucose index; TyG-BMI: TyG-body mass index.

**
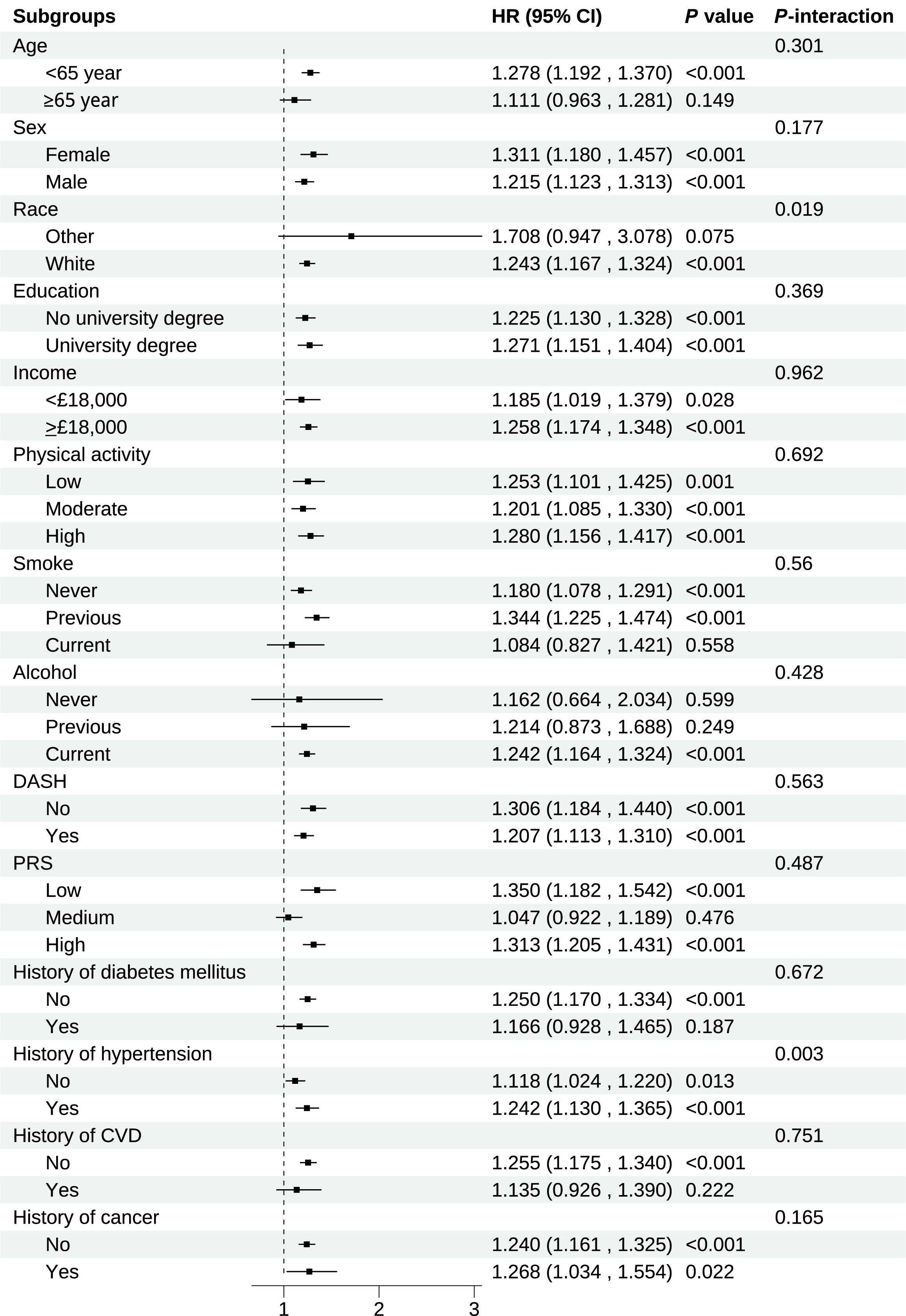
**

**Figure S8. Subgroup analysis of the association between TyG-WC and atrial fibrillation.**

Models were adjusted by age, sex, race, education, income, physical activity, smoke, alcohol, DASH, PRS, and history of diabetes mellitus, hypertension, other CVD, and cancer. DASH: Dietary approaches to stop hypertension; PRS: Polygenic risk score; CVD: Cardiovascular diseases; TyG: Triglyceride-glucose index; TyG-WC: TyG-waist circumference.

**
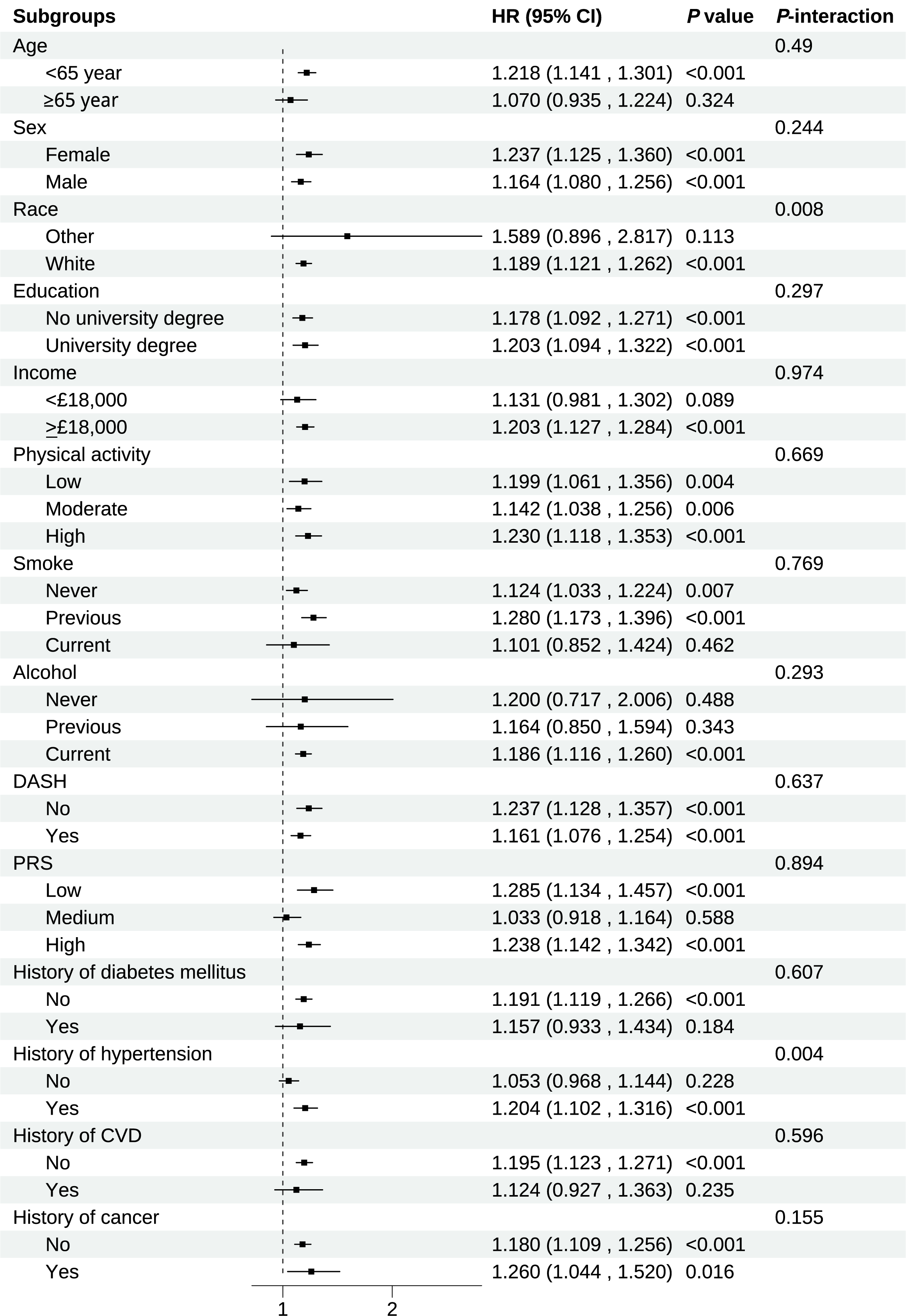
**

**Figure S9. Subgroup analysis of the association between TyG-WHtR and atrial fibrillation.**

Models were adjusted by age, sex, race, education, income, physical activity, smoke, alcohol, DASH, PRS, and history of diabetes mellitus, hypertension, other CVD, and cancer. DASH: Dietary approaches to stop hypertension; PRS: Polygenic risk score; CVD: Cardiovascular diseases; TyG: Triglyceride-glucose index; TyG-WHtR: TyG-waist-to-height ratio.

**
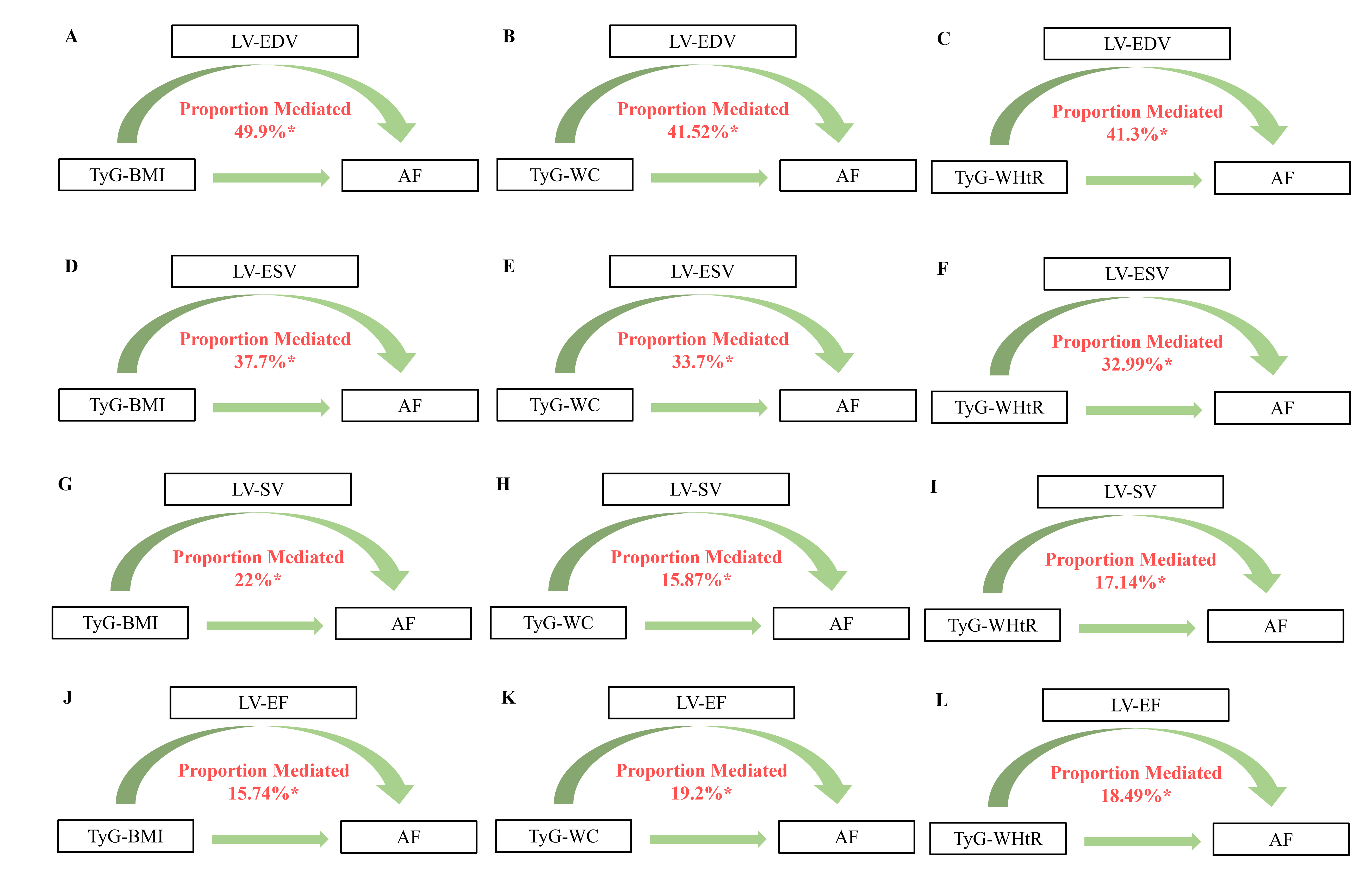
**

**Figure S10. Mediation proportions of LV structure and function in the association between TyG obesity-related derivatives and atrial fibrillation.**

Models were adjusted by age, sex, race, education, income, physical activity, smoke, alcohol, DASH, PRS, and history of diabetes mellitus, hypertension, other CVD, and cancer. DASH: Dietary approaches to stop hypertension; PRS: Polygenic risk score; CVD: Cardiovascular diseases; LV-EDV: Left ventricular end diastolic volume; LV-ESV: Left ventricular end systolic volume; LV-SV: Left ventricular stroke volume; LV-EF: Left ventricular ejection fraction; TyG: Triglyceride-glucose index; TyG-BMI: TyG-body mass index; TyG-WC: TyG-waist circumference; TyG-WHtR: TyG-waist-to-height ratio; AF: atrial fibrillation.

**
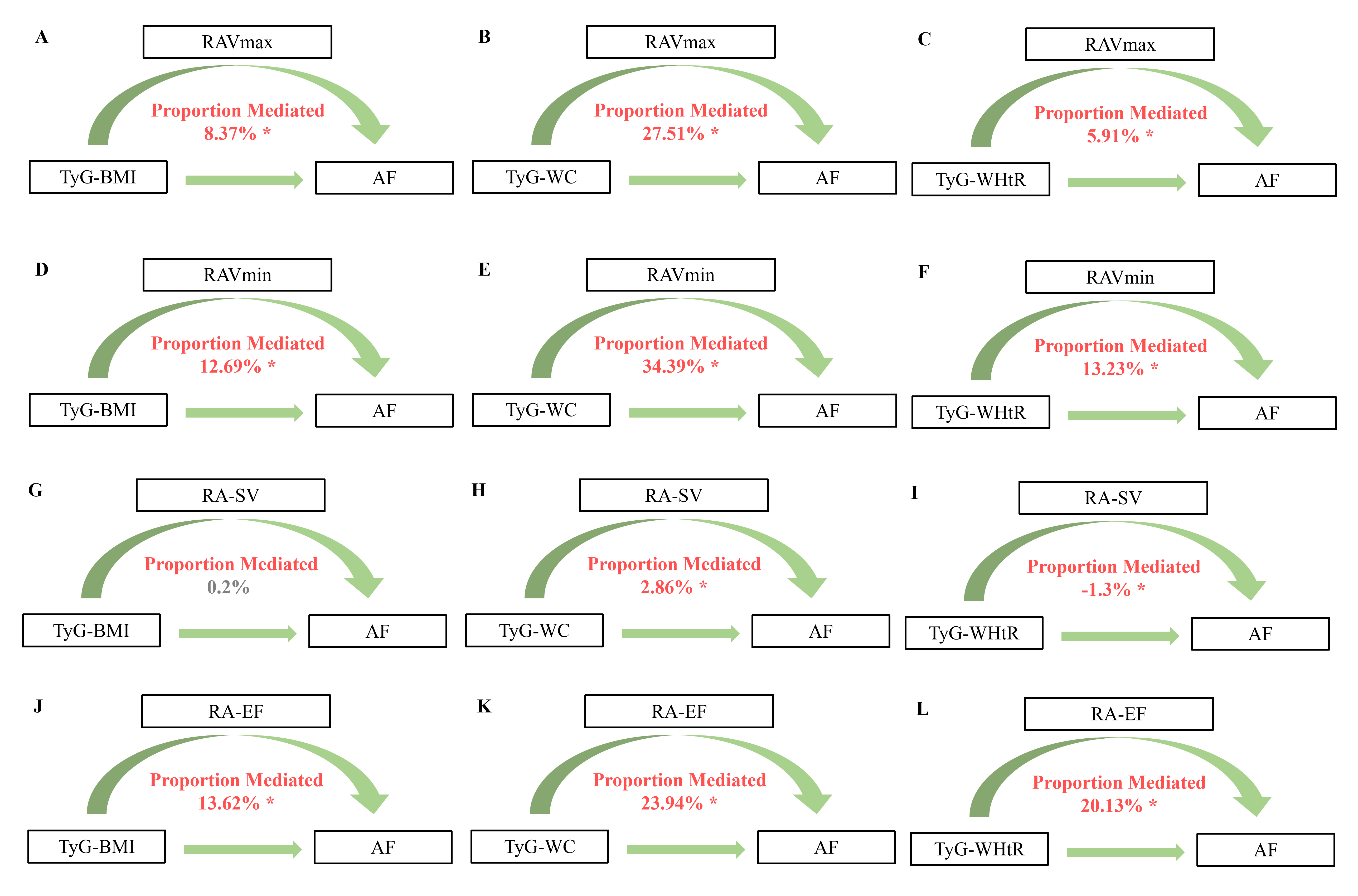
**

**Figure S11. Mediation proportions of RA structure and function in the association between TyG obesity-related derivatives and atrial fibrillation.**

Models were adjusted by age, sex, race, education, income, physical activity, smoke, alcohol, DASH, PRS, and history of diabetes mellitus, hypertension, other CVD, and cancer. DASH: Dietary approaches to stop hypertension; PRS: Polygenic risk score; CVD: Cardiovascular diseases; RAVmax: Right atrial maximum volume; RAVmin: Right atrial minimum volume; RA-SV: Right atrial stroke volume; RA-EF: Right atrial ejection fraction; TyG: Triglyceride-glucose index; TyG-BMI: TyG-body mass index; TyG-WC: TyG-waist circumference; TyG-WHtR: TyG-waist-to-height ratio; AF: atrial fibrillation.

**
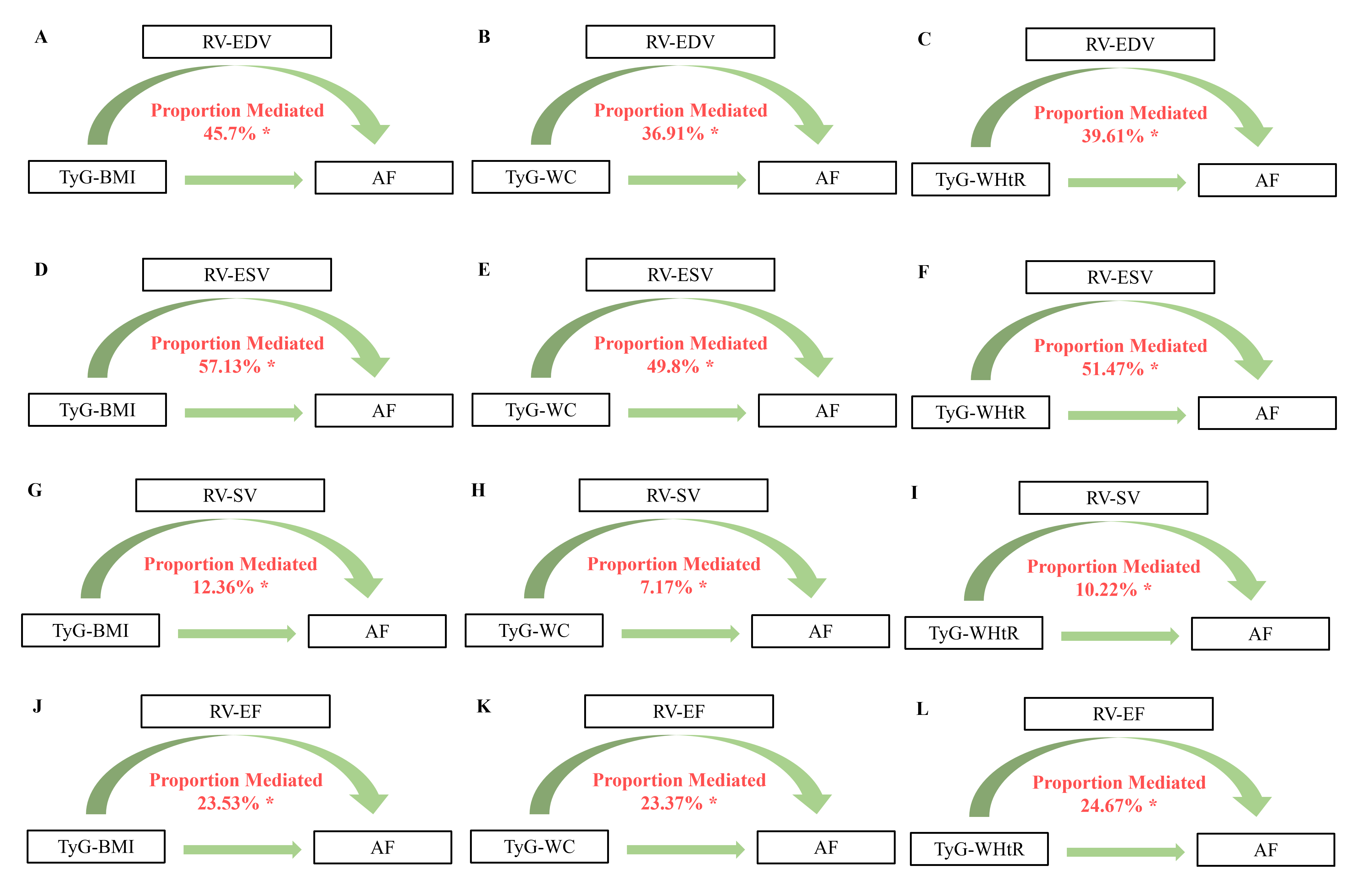
**

**Figure S12. Mediation proportions of RV structure and function in the association between TyG obesity-related derivatives and atrial fibrillation.**

Models were adjusted by age, sex, race, education, income, physical activity, smoke, alcohol, DASH, PRS, and history of diabetes mellitus, hypertension, other CVD, and cancer. DASH: Dietary approaches to stop hypertension; PRS: Polygenic risk score; CVD: Cardiovascular diseases; RV-EDV: Right ventricular end diastolic volume; RV-ESV: Right ventricular end systolic volume; RV-SV: Right ventricular stroke volume; RV-EF: Right ventricular ejection fraction; TyG: Triglyceride-glucose index; TyG-BMI: TyG-body mass index; TyG-WC: TyG-waist circumference; TyG-WHtR: TyG-waist-to-height ratio; AF: atrial fibrillation.

**Table S1. Diagnostic criteria for of atrial fibrillation.**

| Field Name | Field ID | Data coding | Meaning |
| --- | --- | --- | --- |
| Non-cancer illness code, self-reported | 20002 | 1471;  1483. | Atrial fibrillation;  Atrial flutter. |
| Diagnoses - ICD 9 | 41271 | 4273 | Atrial fibrillation and flutter |
| Diagnoses - ICD10 | 41270 | I48;  I48.0;  I48.1;  I48.2;  I48.3;  I48.4;  I48.9. | Atrial fibrillation and flutter;  Paroxysmal atrial fibrillation;  Persistent atrial fibrillation;  Chronic atrial fibrillation;  Typical atrial flutter;  Atypical atrial flutter;  Atrial fibrillation and atrial flutter, unspecified. |
| Underlying (primary) cause of death: ICD10 | 40001 |
| Contributory (secondary) causes of death: ICD10 | 40002 |
| Operative procedures - OPCS4 | 41272 | K62.1;  K62.2;  K62.3;  K62.4. | Percutaneous transluminal ablation of pulmonary vein to left atrium conducting system;  Percutaneous transluminal ablation of atrial wall for atrial flutter;  Percutaneous transluminal ablation of conducting system of heart for atrial flutter NEC;  Percutaneous transluminal internal cardioversion NEC. |

**Table S2. TyG obesity-related derivatives and CMR characteristics of participants with atrial fibrillation and the control group.**

| Characteristic | level | Overall  (n=32500) | Control  (n=31212) | AF  (n=1288) | *P* |
| --- | --- | --- | --- | --- | --- |
| TyG (mean (SD)) |  | 7.04(0.55) | 7.04(0.55) | 7.14(0.57) | <0.001 |
|  | Q1 | 8125(25.00) | 7869(25.21) | 256(19.88) | <0.001 |
|  | Q2 | 8125(25.00) | 7827(25.08) | 298(23.14) |  |
|  | Q3 | 8125(25.00) | 7783(24.94) | 342(26.55) |  |
|  | Q4 | 8125(25.00) | 7733(24.78) | 392(30.43) |  |
| TyG-BMI (mean (SD)) |  | 188.21(38.25) | 187.72(37.99) | 200.04(42.50) | <0.001 |
|  | Q1 | 8125(25.00) | 7912(25.35) | 213(16.54) | <0.001 |
|  | Q2 | 8125(25.00) | 7848(25.14) | 277(21.51) |  |
|  | Q3 | 8125(25.00) | 7789(24.96) | 336(26.09) |  |
|  | Q4 | 8125(25.00) | 7663(24.55) | 462(35.87) |  |
| TyG-WC (mean (SD)) |  | 623.15(119.50) | 621.13(118.78) | 672.13(126.31) | <0.001 |
|  | Q1 | 8125(25.00) | 7959(25.50) | 166(12.89) | <0.001 |
|  | Q2 | 8125(25.00) | 7844(25.13) | 281(21.82) |  |
|  | Q3 | 8125(25.00) | 7786(24.95) | 339(26.32) |  |
|  | Q4 | 8125(25.00) | 7623(24.42) | 502(38.98) |  |
| TyG-WHtR (mean (SD)) |  | 3.67(0.66) | 3.66(0.65) | 3.89(0.71) | <0.001 |
|  | Q1 | 8125(25.00) | 7922(25.38) | 203(15.76) | <0.001 |
|  | Q2 | 8125(25.00) | 7852(25.16) | 273(21.20) |  |
|  | Q3 | 8125(25.00) | 7771(24.90) | 354(27.48) |  |
|  | Q4 | 8125(25.00) | 7667(24.56) | 458(35.56) |  |
| LV-EDV (mL) |  | 147.92(33.80) | 147.32(33.30) | 162.40(41.69) | <0.001 |
| LV-ESV (mL) |  | 60.45(19.41) | 59.99(18.78) | 71.37(28.82) | <0.001 |
| LV-SV (mL) |  | 87.47(19.21) | 87.32(19.02) | 91.03(23.21) | <0.001 |
| LV-EF (%) |  | 59.55(6.13) | 59.67(5.97) | 56.69(8.77) | <0.001 |
| RV-EDV (mL) |  | 156.63(37.13) | 156.18(36.93) | 167.57(40.24) | <0.001 |
| RV-ESV (mL) |  | 67.50(21.12) | 67.13(20.90) | 76.46(24.24) | <0.001 |
| RV-SV (mL) |  | 89.13(20.29) | 89.04(20.10) | 91.12(24.36) | <0.001 |
| RV-EF (%) |  | 57.34(6.15) | 57.45(6.01) | 54.60(8.39) | <0.001 |
| LAVmax (mL) |  | 72.50(23.09) | 71.62(21.92) | 93.88(36.55) | <0.001 |
| LAVmin (mL) |  | 29.13(14.98) | 28.27(13.01) | 50.12(33.24) | <0.001 |
| LA-SV (mL) |  | 43.37(11.76) | 43.35(11.68) | 43.75(13.64) | 0.229 |
| LA-EF (%) |  | 61.32(9.29) | 61.77(8.66) | 50.58(15.43) | <0.001 |
| RAVmax (mL) |  | 86.21(27.40) | 85.52(26.67) | 102.87(37.59) | <0.001 |
| RAVmin (mL) |  | 46.07(18.72) | 45.44(17.66) | 61.30(32.37) | <0.001 |
| RA-SV (mL) |  | 40.14(13.32) | 40.08(13.24) | 41.57(14.98) | <0.001 |
| RA-EF (%) |  | 47.23(9.33) | 47.42(9.13) | 42.44(12.49) | <0.001 |

CMR: Cardiac magnetic resonance imaging; LV-EDV: Left ventricularend diastolic volume; LV-ESV: Left ventricularend systolic volume; LV-SV: Left ventricularstroke volume; LV-EF: Left ventricularejection fraction; RV-EDV: Right ventricularend diastolic volume; RV-ESV: Right ventricularend systolic volume; RV-SV: Right ventricularstroke volume; RV-EF: Right ventricularejection fraction; LAVmax: Left atrial maximum volume; LAVmin: Left atrial minimum volume; LA-SV: Left atrial stroke volume; LA-EF: Left atrial ejection fraction; RAVmax: Right atrial maximum volume; RAVmin: Right atrial minimum volume; RA-SV: Right atrial stroke volume; RA-EF: Right atrial ejection fraction; TyG: Triglyceride-glucose index; TyG-BMI: TyG-body mass index; TyG-WC: TyG-waist circumference; TyG-WHtR: TyG-waist-to-height ratio.

**Table S3. Baseline characteristics of participants according to TyG quartiles.**

|  | level | Q1  (n=8125) | Q2  (n=8125) | Q3  (n=8125) | Q4  (n=8125) | *P* |
| --- | --- | --- | --- | --- | --- | --- |
| Age(year) |  | 52.78(7.46) | 54.85(7.39) | 55.86(7.31) | 55.77(7.25) | <0.001 |
|  | <65 | 7597(93.50) | 7342(90.36) | 7173(88.28) | 7198(88.59) | <0.001 |
|  | ≥65 | 528(6.50) | 783(9.64) | 952(11.72) | 927(11.41) |  |
| Sex(%) | Female | 5592(68.82) | 4670(57.48) | 3831(47.15) | 2752(33.87) | <0.001 |
|  | Male | 2533(31.18) | 3455(42.52) | 4294(52.85) | 5373(66.13) |  |
| Race(%) | Other | 266(3.27) | 213(2.62) | 230(2.83) | 216(2.66) | 0.048 |
|  | White | 7859(96.73) | 7912(97.38) | 7895(97.17) | 7909(97.34) |  |
| Education(%) | Nouniversity degree | 3972(48.89) | 4285(52.74) | 4489(55.25) | 4644(57.16) | <0.001 |
|  | University degree | 4153(51.11) | 3840(47.26) | 3636(44.75) | 3481(42.84) |  |
| Income(%) | <£18,000 | 795(9.78) | 957(11.78) | 1038(12.78) | 1055(12.98) | <0.001 |
|  | >£18,000 | 7330(90.22) | 7168(88.22) | 7087(87.22) | 7070(87.02) |  |
| BMI(kg/m^2^) |  | 24.53(3.39) | 25.94(3.95) | 27.26(4.16) | 28.64(4.21) | <0.001 |
|  | <25 | 5024(61.83) | 3751(46.17) | 2506(30.84) | 1446(17.80) | <0.001 |
|  | ≥30 | 556(6.84) | 1047(12.89) | 1669(20.54) | 2563(31.54) |  |
|  | 25~29 | 2545(31.32) | 3327(40.95) | 3950(48.62) | 4116(50.66) |  |
| Physical activity(%) | Low | 1254(15.43) | 1442(17.75) | 1572(19.35) | 1798(22.13) | <0.001 |
|  | Moderate | 3306(40.69) | 3375(41.54) | 3364(41.40) | 3393(41.76) |  |
|  | High | 3565(43.88) | 3308(40.71) | 3189(39.25) | 2934(36.11) |  |
| Smoke(%) | Never | 5247(64.58) | 5085(62.58) | 4852(59.72) | 4525(55.69) | <0.001 |
|  | Previous | 2468(30.38) | 2589(31.86) | 2748(33.82) | 2933(36.10) |  |
|  | Current | 410(5.05) | 451(5.55) | 525(6.46) | 667(8.21) |  |
| Alcohol(%) | Never | 191(2.35) | 203(2.50) | 204(2.51) | 221(2.72) | 0.214 |
|  | Previous | 157(1.93) | 188(2.31) | 171(2.10) | 199(2.45) |  |
|  | Current | 7777(95.72) | 7734(95.19) | 7750(95.38) | 7705(94.83) |  |
| DASH(%) | No | 2695(34.30) | 2906(37.18) | 3117(39.75) | 3371(43.13) | <0.001 |
|  | Yes | 5163(65.70) | 4911(62.82) | 4725(60.25) | 4444(56.87) |  |
| PRS |  | 0.09(0.92) | 0.08(0.91) | 0.09(0.90) | 0.08(0.91) | 0.925 |
| History of diabetesmellitus(%) | No | 8049(99.06) | 8000(98.46) | 7905(97.29) | 7648(94.13) | <0.001 |
|  | Yes | 76(0.94) | 125(1.54) | 220(2.71) | 477(5.87) |  |
| History of hypertension(%) | No | 7051(86.78) | 6562(80.76) | 6070(74.71) | 5707(70.24) | <0.001 |
|  | Yes | 1074(13.22) | 1563(19.24) | 2055(25.29) | 2418(29.76) |  |
| History of other CVD(%) | No | 7916(97.43) | 7874(96.91) | 7775(95.69) | 7768(95.61) | <0.001 |
|  | Yes | 209(2.57) | 251(3.09) | 350(4.31) | 357(4.39) |  |
| History of Cancer(%) | No | 7634(93.96) | 7575(93.23) | 7591(93.43) | 7555(92.98) | 0.08 |
|  | Yes | 491(6.04) | 550(6.77) | 534(6.57) | 570(7.02) |  |
| LV-EDV (mL) |  | 144.82(32.14) | 146.45(33.86) | 148.72(34.66) | 151.67(34.12) | <0.001 |
| LV-ESV (mL) |  | 58.59(18.17) | 59.61(19.41) | 61.14(20.29) | 62.43(19.48) | <0.001 |
| LV-SV (mL) |  | 86.22(18.32) | 86.84(19.14) | 87.58(19.48) | 89.24(19.75) | <0.001 |
| LV-EF (%) |  | 59.89(5.79) | 59.74(6.10) | 59.34(6.31) | 59.22(6.28) | <0.001 |
| RV-EDV (mL) |  | 152.55(35.50) | 154.79(37.10) | 157.61(37.71) | 161.56(37.57) | <0.001 |
| RV-ESV (mL) |  | 65.05(20.12) | 66.48(21.32) | 68.14(21.54) | 70.33(21.12) | <0.001 |
| RV-SV (mL) |  | 87.50(19.29) | 88.32(20.00) | 89.47(20.63) | 91.23(21.00) | <0.001 |
| RV-EF (%) |  | 57.77(5.85) | 57.53(6.15) | 57.20(6.30) | 56.83(6.24) | <0.001 |
| LAVmax (mL) |  | 71.58(21.86) | 71.97(23.13) | 72.57(23.55) | 73.89(23.71) | <0.001 |
| LAVmin (mL) |  | 28.47(13.71) | 28.91(15.10) | 29.18(15.39) | 29.97(15.63) | <0.001 |
| LA-SV (mL) |  | 43.11(11.32) | 43.06(11.67) | 43.39(11.95) | 43.91(12.08) | <0.001 |
| LA-EF (%) |  | 61.49(8.61) | 61.37(9.23) | 61.39(9.47) | 61.05(9.80) | 0.017 |
| RAVmax (mL) |  | 87.86(27.08) | 86.62(27.79) | 85.79(27.67) | 84.56(26.95) | <0.001 |
| RAVmin (mL) |  | 46.58(18.40) | 46.18(19.31) | 45.99(18.87) | 45.54(18.29) | 0.004 |
| RA-SV (mL) |  | 41.28(13.23) | 40.44(13.15) | 39.80(13.42) | 39.02(13.37) | <0.001 |
| RA-EF (%) |  | 47.60(8.97) | 47.46(9.36) | 47.08(9.39) | 46.76(9.57) | <0.001 |

TyG: Triglyceride-glucose index; BMI: Body mass index; DASH: Dietary approaches to stop hypertension; PRS: Polygenic risk score; CVD: Cardiovascular diseases; LV-EDV: Left ventricularend diastolic volume; LV-ESV: Left ventricularend systolic volume; LV-SV: Left ventricularstroke volume; LV-EF: Left ventricularejection fraction; RV-EDV: Right ventricularend diastolic volume; RV-ESV: Right ventricularend systolic volume; RV-SV: Right ventricularstroke volume; RV-EF: Right ventricularejection fraction; LAVmax: Left atrial maximum volume; LAVmin: Left atrial minimum volume; LA-SV: Left atrial stroke volume; LA-EF: Left atrial ejection fraction; RAVmax: Right atrial maximum volume; RAVmin: Right atrial minimum volume; RA-SV: Right atrial stroke volume; RA-EF: Right atrial ejection fraction..

**Table S4. Baseline characteristics of participants according to TyG-BMI quartiles.**

|  | level | Q1  (n=8125) | Q2  (n=8125) | Q3  (n=8125) | Q4  (n=8125) | *P* |
| --- | --- | --- | --- | --- | --- | --- |
| Age(year) |  | 53.18(7.46) | 55.11(7.51) | 55.71(7.32) | 55.27(7.27) | <0.001 |
|  | <65 | 7568(93.14) | 7249(89.22) | 7204(88.66) | 7289(89.71) | <0.001 |
|  | ≥65 | 557(6.86) | 876(10.78) | 921(11.34) | 836(10.29) |  |
| Sex(%) | Female | 5808(71.48) | 4350(53.54) | 3323(40.90) | 3364(41.40) | <0.001 |
|  | Male | 2317(28.52) | 3775(46.46) | 4802(59.10) | 4761(58.60) |  |
| Race(%) | Other | 223(2.74) | 253(3.11) | 235(2.89) | 214(2.63) | 0.284 |
|  | White | 7902(97.26) | 7872(96.89) | 7890(97.11) | 7911(97.37) |  |
| Education(%) | Nouniversity degree | 3750(46.15) | 4212(51.84) | 4528(55.73) | 4900(60.31) | <0.001 |
|  | University degree | 4375(53.85) | 3913(48.16) | 3597(44.27) | 3225(39.69) |  |
| Income(%) | <£18,000 | 850(10.46) | 888(10.93) | 979(12.05) | 1128(13.88) | <0.001 |
|  | >£18,000 | 7275(89.54) | 7237(89.07) | 7146(87.95) | 6997(86.12) |  |
| BMI(kg/m^2^) |  | 22.24(1.65) | 24.96(1.44) | 27.30(1.69) | 31.87(3.72) | <0.001 |
|  | <25 | 7828(96.34) | 4273(52.59) | 619(7.62) | 7(0.09) | <0.001 |
|  | ≥30 | 0(0.00) | 6(0.07) | 506(6.23) | 5323(65.51) |  |
|  | 25~29 | 297(3.66) | 3846(47.34) | 7000(86.15) | 2795(34.40) |  |
| Physical activity(%) | Low | 1128(13.88) | 1313(16.16) | 1567(19.29) | 2058(25.33) | <0.001 |
|  | Moderate | 3397(41.81) | 3304(40.66) | 3358(41.33) | 3379(41.59) |  |
|  | High | 3600(44.31) | 3508(43.18) | 3200(39.38) | 2688(33.08) |  |
| Smoke(%) | Never | 5441(66.97) | 5132(63.16) | 4709(57.96) | 4427(54.49) | <0.001 |
|  | Previous | 2241(27.58) | 2516(30.97) | 2846(35.03) | 3135(38.58) |  |
|  | Current | 443(5.45) | 477(5.87) | 570(7.02) | 563(6.93) |  |
| Alcohol(%) | Never | 216(2.66) | 196(2.41) | 192(2.36) | 215(2.65) | 0.148 |
|  | Previous | 160(1.97) | 164(2.02) | 188(2.31) | 203(2.50) |  |
|  | Current | 7749(95.37) | 7765(95.57) | 7745(95.32) | 7707(94.86) |  |
| DASH(%) | No | 2514(31.97) | 2873(36.62) | 3196(40.84) | 3506(44.97) | <0.001 |
|  | Yes | 5350(68.03) | 4973(63.38) | 4629(59.16) | 4291(55.03) |  |
| PRS |  | 0.08(0.91) | 0.08(0.92) | 0.09(0.90) | 0.10(0.91) | 0.193 |
| History of diabetesmellitus(%) | No | 8069(99.31) | 8012(98.61) | 7932(97.62) | 7589(93.40) | <0.001 |
|  | Yes | 56(0.69) | 113(1.39) | 193(2.38) | 536(6.60) |  |
| History of hypertension(%) | No | 7321(90.10) | 6696(82.41) | 6159(75.80) | 5214(64.17) | <0.001 |
|  | Yes | 804(9.90) | 1429(17.59) | 1966(24.20) | 2911(35.83) |  |
| History of other CVD(%) | No | 7990(98.34) | 7864(96.79) | 7795(95.94) | 7684(94.57) | <0.001 |
|  | Yes | 135(1.66) | 261(3.21) | 330(4.06) | 441(5.43) |  |
| History of Cancer(%) | No | 7592(93.44) | 7590(93.42) | 7591(93.43) | 7582(93.32) | 0.989 |
|  | Yes | 533(6.56) | 535(6.58) | 534(6.57) | 543(6.68) |  |
| LV-EDV (mL) |  | 138.00(30.21) | 145.69(33.51) | 151.16(33.88) | 156.81(34.57) | <0.001 |
| LV-ESV (mL) |  | 55.54(16.81) | 59.52(19.20) | 62.28(19.64) | 64.45(20.62) | <0.001 |
| LV-SV (mL) |  | 82.46(17.44) | 86.17(18.90) | 88.89(19.12) | 92.36(19.93) | <0.001 |
| LV-EF (%) |  | 60.10(5.78) | 59.58(6.02) | 59.22(6.13) | 59.30(6.53) | <0.001 |
| RV-EDV (mL) |  | 145.10(33.93) | 154.55(36.72) | 160.81(37.19) | 166.06(37.28) | <0.001 |
| RV-ESV (mL) |  | 61.48(19.44) | 66.44(20.92) | 69.92(21.26) | 72.16(21.26) | <0.001 |
| RV-SV (mL) |  | 83.61(18.28) | 88.11(19.87) | 90.89(20.39) | 93.90(21.08) | <0.001 |
| RV-EF (%) |  | 58.09(5.95) | 57.45(6.01) | 56.92(6.19) | 56.88(6.35) | <0.001 |
| LAVmax (mL) |  | 66.64(20.16) | 69.95(22.02) | 73.79(22.98) | 79.63(24.90) | <0.001 |
| LAVmin (mL) |  | 26.32(12.39) | 27.62(14.22) | 29.58(14.94) | 33.02(17.14) | <0.001 |
| LA-SV (mL) |  | 40.32(10.58) | 42.34(11.28) | 44.21(11.76) | 46.61(12.42) | <0.001 |
| LA-EF (%) |  | 61.74(8.52) | 61.96(9.07) | 61.44(9.47) | 60.16(9.94) | <0.001 |
| RAVmax (mL) |  | 86.05(26.34) | 87.19(28.19) | 86.86(27.43) | 84.73(27.53) | <0.001 |
| RAVmin (mL) |  | 45.62(18.06) | 46.80(19.45) | 46.64(18.61) | 45.23(18.70) | <0.001 |
| RA-SV (mL) |  | 40.43(12.74) | 40.40(13.29) | 40.22(13.38) | 39.49(13.82) | <0.001 |
| RA-EF (%) |  | 47.62(8.94) | 47.12(9.34) | 46.96(9.29) | 47.21(9.74) | <0.001 |

TyG: Triglyceride-glucose index; BMI: Body mass index; DASH: Dietary approaches to stop hypertension; PRS: Polygenic risk score; CVD: Cardiovascular diseases; LV-EDV: Left ventricularend diastolic volume; LV-ESV: Left ventricularend systolic volume; LV-SV: Left ventricularstroke volume; LV-EF: Left ventricularejection fraction; RV-EDV: Right ventricularend diastolic volume; RV-ESV: Right ventricularend systolic volume; RV-SV: Right ventricularstroke volume; RV-EF: Right ventricularejection fraction; LAVmax: Left atrial maximum volume; LAVmin: Left atrial minimum volume; LA-SV: Left atrial stroke volume; LA-EF: Left atrial ejection fraction; RAVmax: Right atrial maximum volume; RAVmin: Right atrial minimum volume; RA-SV: Right atrial stroke volume; RA-EF: Right atrial ejection fraction.

**Table S5. Baseline characteristics of participants according to TyG-WC quartiles.**

|  | level | Q1  (n=8125) | Q2  (n=8125) | Q3  (n=8125) | Q4  (n=8125) | *P* |
| --- | --- | --- | --- | --- | --- | --- |
| Age(year) |  | 52.84 (7.34) | 54.84 (7.45) | 55.75 (7.41) | 55.83 (7.24) | <0.001 |
|  | <65 | 7662 (94.30) | 7302 (89.87) | 7157 (88.09) | 7189 (88.48) | <0.001 |
|  | ≥65 | 463 ( 5.70) | 823 (10.13) | 968 (11.91) | 936 (11.52) |  |
| Sex(%) | Female | 7209 (88.73) | 4842 (59.59) | 2953 (36.34) | 1841 (22.66) | <0.001 |
|  | Male | 916 (11.27) | 3283 (40.41) | 5172 (63.66) | 6284 (77.34) |  |
| Race(%) | Other | 225 ( 2.77) | 267 ( 3.29) | 233 ( 2.87) | 200 ( 2.46) | 0.017 |
|  | White | 7900 (97.23) | 7858 (96.71) | 7892 (97.13) | 7925 (97.54) |  |
| Education(%) | Nouniversity degree | 4002 (49.26) | 4322 (53.19) | 4336 (53.37) | 4730 (58.22) | <0.001 |
|  | University degree | 4123 (50.74) | 3803 (46.81) | 3789 (46.63) | 3395 (41.78) |  |
| Income(%) | <£18,000 | 859 (10.57) | 1011 (12.44) | 924 (11.37) | 1051 (12.94) | <0.001 |
|  | >£18,000 | 7266 (89.43) | 7114 (87.56) | 7201 (88.63) | 7074 (87.06) |  |
| BMI(kg/m^2^) |  | 22.95 (2.27) | 25.30 (2.57) | 27.31 (2.93) | 30.81 (4.23) | <0.001 |
|  | <25 | 6753 (83.11) | 4032 (49.62) | 1673 (20.59) | 269 ( 3.31) | <0.001 |
|  | ≥30 | 37 ( 0.46) | 411 ( 5.06) | 1275 (15.69) | 4112 (50.61) |  |
|  | 25~29 | 1335 (16.43) | 3682 (45.32) | 5177 (63.72) | 3744 (46.08) |  |
| Physical activity(%) | Low | 1123 (13.82) | 1336 (16.44) | 1573 (19.36) | 2034 (25.03) | <0.001 |
|  | Moderate | 3375 (41.54) | 3313 (40.78) | 3337 (41.07) | 3413 (42.01) |  |
|  | High | 3627 (44.64) | 3476 (42.78) | 3215 (39.57) | 2678 (32.96) |  |
| Smoke(%) | Never | 5460 (67.20) | 5170 (63.63) | 4792 (58.98) | 4287 (52.76) | <0.001 |
|  | Previous | 2275 (28.00) | 2464 (30.33) | 2796 (34.41) | 3203 (39.42) |  |
|  | Current | 390 ( 4.80) | 491 ( 6.04) | 537 ( 6.61) | 635 ( 7.82) |  |
| Alcohol(%) | Never | 235 ( 2.89) | 213 ( 2.62) | 197 ( 2.42) | 174 ( 2.14) | 0.01 |
|  | Previous | 170 ( 2.09) | 164 ( 2.02) | 172 ( 2.12) | 209 ( 2.57) |  |
|  | Current | 7720 (95.02) | 7748 (95.36) | 7756 (95.46) | 7742 (95.29) |  |
| DASH(%) | No | 2447 (31.12) | 2849 (36.30) | 3158 (40.46) | 3635 (46.52) | <0.001 |
|  | Yes | 5417 (68.88) | 5000 (63.70) | 4647 (59.54) | 4179 (53.48) |  |
| PRS |  | 0.07 (0.91) | 0.08 (0.91) | 0.09 (0.91) | 0.10 (0.91) | 0.282 |
| History of diabetesmellitus(%) | No | 8073 (99.36) | 8022 (98.73) | 7947 (97.81) | 7560 (93.05) | <0.001 |
|  | Yes | 52 ( 0.64) | 103 ( 1.27) | 178 ( 2.19) | 565 ( 6.95) |  |
| History of hypertension(%) | No | 7324 (90.14) | 6659 (81.96) | 6189 (76.17) | 5218 (64.22) | <0.001 |
|  | Yes | 801 ( 9.86) | 1466 (18.04) | 1936 (23.83) | 2907 (35.78) |  |
| History of other CVD(%) | No | 8019 (98.70) | 7858 (96.71) | 7803 (96.04) | 7653 (94.19) | <0.001 |
|  | Yes | 106(1.30) | 267(3.29) | 322(3.96) | 472(5.81) |  |
| History of Cancer(%) | No | 7579(93.28) | 7567(93.13) | 7583(93.33) | 7626(93.86) | 0.265 |
|  | Yes | 546(6.72) | 558(6.87) | 542(6.67) | 499(6.14) |  |
| LV-EDV (mL) |  | 132.19(26.22) | 144.05(33.33) | 153.27(33.64) | 162.16(33.87) | <0.001 |
| LV-ESV (mL) |  | 52.24(14.58) | 58.59(18.76) | 63.42(19.84) | 67.53(20.46) | <0.001 |
| LV-SV (mL) |  | 79.95(15.66) | 85.46(18.92) | 89.84(19.08) | 94.63(19.80) | <0.001 |
| LV-EF (%) |  | 60.73(5.55) | 59.74(5.99) | 59.03(6.24) | 58.70(6.49) | <0.001 |
| RV-EDV (mL) |  | 138.10(29.05) | 152.37(36.68) | 163.37(36.87) | 172.68(36.09) | <0.001 |
| RV-ESV (mL) |  | 57.43(16.56) | 65.17(20.93) | 71.29(21.23) | 76.11(20.64) | <0.001 |
| RV-SV (mL) |  | 80.66(16.19) | 87.21(19.77) | 92.08(20.32) | 96.56(21.00) | <0.001 |
| RV-EF (%) |  | 58.75(5.65) | 57.70(6.15) | 56.75(6.16) | 56.15(6.29) | <0.001 |
| LAVmax (mL) |  | 66.44(18.92) | 69.78(22.00) | 73.75(23.24) | 80.04(25.46) | <0.001 |
| LAVmin (mL) |  | 26.10(11.33) | 27.49(14.14) | 29.55(15.14) | 33.39(17.61) | <0.001 |
| LA-SV (mL) |  | 40.34(10.08) | 42.29(11.31) | 44.20(11.91) | 46.65(12.65) | <0.001 |
| LA-EF (%) |  | 61.82(8.24) | 62.05(9.13) | 61.44(9.43) | 59.99(10.12) | <0.001 |
| RAVmax (mL) |  | 81.43(22.89) | 86.00(28.69) | 88.48(28.56) | 88.92(28.37) | <0.001 |
| RAVmin (mL) |  | 42.20(15.35) | 45.96(19.60) | 47.80(19.45) | 48.32(19.53) | <0.001 |
| RA-SV (mL) |  | 39.23(11.76) | 40.04(13.52) | 40.68(13.76) | 40.59(14.06) | <0.001 |
| RA-EF (%) |  | 48.63(8.78) | 47.35(9.23) | 46.70(9.43) | 46.23(9.69) | <0.001 |

TyG: Triglyceride-glucose index; TyG-WC: TyG-waist circumference; BMI: Body mass index; DASH: Dietary approaches to stop hypertension; PRS: Polygenic risk score; CVD: Cardiovascular diseases; LV-EDV: Left ventricularend diastolic volume; LV-ESV: Left ventricularend systolic volume; LV-SV: Left ventricularstroke volume; LV-EF: Left ventricularejection fraction; RV-EDV: Right ventricularend diastolic volume; RV-ESV: Right ventricularend systolic volume; RV-SV: Right ventricularstroke volume; RV-EF: Right ventricularejection fraction; LAVmax: Left trium maximum volume; LAVmin: Left atrial minimum volume; LA-SV: Left atrial stroke volume; LA-EF: Left atrial ejection fraction; RAVmax: Right atrial maximum volume; RAVmin: Right atrial minimum volume; RA-SV: Right atrial stroke volume; RA-EF: Right atrial ejection fraction.

**Table S6. Baseline characteristics of participants according to TyG-WHtR quartiles.**

|  | level | Q1  (n=8125) | Q2  (n=8125) | Q3  (n=8125) | Q4  (n=8125) | *P* |
| --- | --- | --- | --- | --- | --- | --- |
| Age(year) |  | 52.54(7.31) | 54.83(7.44) | 55.80(7.31) | 56.09(7.23) | <0.001 |
|  | <65 | 7685(94.58) | 7323(90.13) | 7188(88.47) | 7114(87.56) | <0.001 |
|  | ≥65 | 440(5.42) | 802(9.87) | 937(11.53) | 1011(12.44) |  |
| Sex(%) | Female | 6342(78.06) | 4392(54.06) | 3246(39.95) | 2865(35.26) | <0.001 |
|  | Male | 1783(21.94) | 3733(45.94) | 4879(60.05) | 5260(64.74) |  |
| Race(%) | Other | 197(2.42) | 251(3.09) | 240(2.95) | 237(2.92) | 0.059 |
|  | White | 7928(97.58) | 7874(96.91) | 7885(97.05) | 7888(97.08) |  |
| Education(%) | Nouniversity degree | 3845(47.32) | 4216(51.89) | 4478(55.11) | 4851(59.70) | <0.001 |
|  | University degree | 4280(52.68) | 3909(48.11) | 3647(44.89) | 3274(40.30) |  |
| Income(%) | <£18,000 | 783(9.64) | 954(11.74) | 929(11.43) | 1179(14.51) | <0.001 |
|  | >£18,000 | 7342(90.36) | 7171(88.26) | 7196(88.57) | 6946(85.49) |  |
| BMI(kg/m^2^) |  | 22.79(2.13) | 25.18(2.30) | 27.24(2.61) | 31.16(4.14) | <0.001 |
|  | <25 | 6953(85.58) | 4089(50.33) | 1508(18.56) | 177(2.18) | <0.001 |
|  | ≥30 | 16(0.20) | 230(2.83) | 1106(13.61) | 4483(55.18) |  |
|  | 25~29 | 1156(14.23) | 3806(46.84) | 5511(67.83) | 3465(42.65) |  |
| Physical activity(%) | Low | 1108(13.64) | 1317(16.21) | 1592(19.59) | 2049(25.22) | <0.001 |
|  | Moderate | 3340(41.11) | 3349(41.22) | 3366(41.43) | 3383(41.64) |  |
|  | High | 3677(45.26) | 3459(42.57) | 3167(38.98) | 2693(33.14) |  |
| Smoke(%) | Never | 5450(67.08) | 5125(63.08) | 4795(59.02) | 4339(53.40) | <0.001 |
|  | Previous | 2254(27.74) | 2514(30.94) | 2798(34.44) | 3172(39.04) |  |
|  | Current | 421(5.18) | 486(5.98) | 532(6.55) | 614(7.56) |  |
| Alcohol(%) | Never | 203(2.50) | 207(2.55) | 203(2.50) | 206(2.54) | 0.053 |
|  | Previous | 160(1.97) | 163(2.01) | 174(2.14) | 218(2.68) |  |
|  | Current | 7762(95.53) | 7755(95.45) | 7748(95.36) | 7701(94.78) |  |
| DASH(%) | No | 2466(31.37) | 2871(36.49) | 3176(40.63) | 3576(45.92) | <0.001 |
|  | Yes | 5394(68.63) | 4997(63.51) | 4641(59.37) | 4211(54.08) |  |
| PRS |  | 0.08(0.91) | 0.09(0.92) | 0.08(0.90) | 0.10(0.91) | 0.65 |
| History of diabetesmellitus(%) | No | 8074(99.37) | 8026(98.78) | 7953(97.88) | 7549(92.91) | <0.001 |
|  | Yes | 51(0.63) | 99(1.22) | 172(2.12) | 576(7.09) |  |
| History of hypertension(%) | No | 7372(90.73) | 6705(82.52) | 6173(75.98) | 5140(63.26) | <0.001 |
|  | Yes | 753(9.27) | 1420(17.48) | 1952(24.02) | 2985(36.74) |  |
| History of other CVD(%) | No | 8022(98.73) | 7860(96.74) | 7798(95.98) | 7653(94.19) | <0.001 |
|  | Yes | 103(1.27) | 265(3.26) | 327(4.02) | 472(5.81) |  |
| History of Cancer(%) | No | 7606(93.61) | 7590(93.42) | 7575(93.23) | 7584(93.34) | 0.796 |
|  | Yes | 519(6.39) | 535(6.58) | 550(6.77) | 541(6.66) |  |
| LV-EDV (mL) |  | 139.29(30.45) | 146.49(34.31) | 151.08(33.85) | 154.80(34.45) | <0.001 |
| LV-ESV (mL) |  | 55.83(16.73) | 59.97(19.78) | 62.33(19.44) | 63.65(20.55) | <0.001 |
| LV-SV (mL) |  | 83.46(17.66) | 86.52(19.06) | 88.75(19.47) | 91.15(19.76) | <0.001 |
| LV-EF (%) |  | 60.23(5.64) | 59.53(6.05) | 59.14(6.26) | 59.29(6.48) | <0.001 |
| RV-EDV (mL) |  | 146.24(34.12) | 155.48(37.76) | 160.87(37.45) | 163.91(36.64) | <0.001 |
| RV-ESV (mL) |  | 61.81(19.52) | 66.89(21.52) | 70.01(21.42) | 71.30(20.69) | <0.001 |
| RV-SV (mL) |  | 84.43(18.29) | 88.59(20.29) | 90.87(20.56) | 92.62(20.97) | <0.001 |
| RV-EF (%) |  | 58.19(5.81) | 57.45(6.11) | 56.90(6.26) | 56.81(6.31) | <0.001 |
| LAVmax (mL) |  | 68.30(20.21) | 70.34(22.31) | 73.34(23.49) | 78.03(24.93) | <0.001 |
| LAVmin (mL) |  | 27.01(12.09) | 27.71(14.30) | 29.44(15.56) | 32.37(16.98) | <0.001 |
| LA-SV (mL) |  | 41.29(10.80) | 42.63(11.45) | 43.91(11.86) | 45.65(12.43) | <0.001 |
| LA-EF (%) |  | 61.58(8.31) | 62.04(9.03) | 61.49(9.59) | 60.19(10.04) | <0.001 |
| RAVmax (mL) |  | 86.29(26.36) | 87.42(28.14) | 86.93(27.71) | 84.20(27.25) | <0.001 |
| RAVmin (mL) |  | 45.51(17.93) | 46.91(19.46) | 46.78(18.77) | 45.09(18.64) | <0.001 |
| RA-SV (mL) |  | 40.78(12.76) | 40.50(13.40) | 40.15(13.44) | 39.11(13.60) | <0.001 |
| RA-EF (%) |  | 47.87(8.84) | 47.10(9.37) | 46.85(9.26) | 47.08(9.80) | <0.001 |

TyG: Triglyceride-glucose index; TyG-WHtR: TyG-waist-to-height ratio; BMI: Body mass index; DASH: Dietary approaches to stop hypertension; PRS: Polygenic risk score; CVD: Cardiovascular diseases; LV-EDV: Left ventricularend diastolic volume; LV-ESV: Left ventricularend systolic volume; LV-SV: Left ventricularstroke volume; LV-EF: Left ventricularejection fraction; RV-EDV: Right ventricularend diastolic volume; RV-ESV: Right ventricularend systolic volume; RV-SV: Right ventricularstroke volume; RV-EF: Right ventricularejection fraction; LAVmax: Left atrial maximum volume; LAVmin: Left atrial minimum volume; LA-SV: Left atrial stroke volume; LA-EF: Left atrial ejection fraction; RAVmax: Right atrial maximum volume; RAVmin: Right atrial minimum volume; RA-SV: Right atrial stroke volume; RA-EF: Right atrial ejection fraction..

**Table S7. Associations between TyG and CMR indices.**

| CMR  (per 1SD) | Model 1 | | Model 2 | | Model 3 | |
| --- | --- | --- | --- | --- | --- | --- |
|  | β(95%CI) | *P* | β(95%CI) | *P* | β(95%CI) | *P* |
| LV-EDV | 0.081( 0.070, 0.092) | <0.001 | 0.082( 0.071, 0.093) | <0.001 | 0.073( 0.062, 0.084) | <0.001 |
| LV-ESV | 0.078( 0.068, 0.089) | <0.001 | 0.079( 0.068, 0.090) | <0.001 | 0.072( 0.061, 0.083) | <0.001 |
| LV-SV | 0.063( 0.052, 0.074) | <0.001 | 0.064( 0.053, 0.075) | <0.001 | 0.056( 0.045, 0.067) | <0.001 |
| LV-EF | -0.044(-0.055,-0.033) | <0.001 | -0.043(-0.054,-0.032) | <0.001 | -0.043(-0.054,-0.032) | <0.001 |
| RV-EDV | 0.098( 0.087, 0.109) | <0.001 | 0.1( 0.089, 0.111) | <0.001 | 0.095( 0.084, 0.106) | <0.001 |
| RV-ESV | 0.101( 0.090, 0.111) | <0.001 | 0.102( 0.091, 0.113) | <0.001 | 0.1( 0.088, 0.111) | <0.001 |
| RV-SV | 0.074( 0.063, 0.085) | <0.001 | 0.077( 0.066, 0.088) | <0.001 | 0.07( 0.059, 0.082) | <0.001 |
| RV-EF | -0.062(-0.073,-0.051) | <0.001 | -0.059(-0.070,-0.048) | <0.001 | -0.062(-0.074,-0.051) | <0.001 |
| LAVmax | 0.036( 0.025,0.047) | <0.001 | 0.04( 0.029, 0.051) | <0.001 | 0.024( 0.013, 0.035) | <0.001 |
| LAVmin | 0.033( 0.022,0.044) | <0.001 | 0.036( 0.024,0.047) | <0.001 | 0.019( 0.008, 0.030) | <0.001 |
| LA-SV | 0.029( 0.018, 0.040) | <0.001 | 0.033( 0.022, 0.044) | <0.001 | 0.023( 0.012, 0.034) | <0.001 |
| LA-EF | -0.015(-0.026,-0.004) | 0.008 | -0.016(-0.027,-0.005) | 0.005 | -0.006(-0.017, 0.005) | 0.293 |
| RAVmax | -0.046(-0.057,-0.035) | <0.001 | -0.039(-0.050,-0.028) | <0.001 | -0.038(-0.049,-0.027) | <0.001 |
| RAVmin | -0.022(-0.033,-0.011) | <0.001 | -0.014(-0.025,-0.003) | 0.015 | -0.013(-0.024,-0.001) | 0.028 |
| RA-SV | -0.065(-0.076,-0.054) | <0.001 | -0.06(-0.071,-0.049) | <0.001 | -0.061(-0.072,-0.049) | <0.001 |
| RA-EF | -0.037(-0.048,-0.027) | <0.001 | -0.042(-0.053,-0.031) | <0.001 | -0.045(-0.056,-0.034) | <0.001 |

Models were adjusted by age, sex, race, education, income, physical activity, smoke, alcohol, DASH, PRS, and history of diabetes mellitus, hypertension, other CVD, and cancer. DASH: Dietary approaches to stop hypertension; PRS: Polygenic risk score; CVD: Cardiovascular diseases; CMR: Cardiac magnetic resonance imaging; LV-EDV: Left ventricularend diastolic volume; LV-ESV: Left ventricularend systolic volume; LV-SV: Left ventricularstroke volume; LV-EF: Left ventricularejection fraction; RV-EDV: Right ventricularend diastolic volume; RV-ESV: Right ventricularend systolic volume; RV-SV: Right ventricularstroke volume; RV-EF: Right ventricularejection fraction; LAVmax: Left atrial maximum volume; LAVmin: Left atrial minimum volume; LA-SV: Left atrial stroke volume; LA-EF: Left atrial ejection fraction; RAVmax: Right atrial maximum volume; RAVmin: Right atrial minimum volume; RA-SV: Right atrial stroke volume; RA-EF: Right atrial ejection fraction; TyG: Triglyceride-glucose index.

**Table S8. Associations between TyG-BMI and CMR indices.**

| CMR  (per 1SD) | Model 1 | | Model 2 | | Model 3 | |
| --- | --- | --- | --- | --- | --- | --- |
|  | β(95%CI) | *P* | β(95%CI) | *P* | β(95%CI) | *P* |
| LV-EDV | 0.218( 0.207, 0.229) | <0.001 | 0.226( 0.215, 0.237) | <0.001 | 0.221( 0.209, 0.232) | <0.001 |
| LV-ESV | 0.176( 0.165, 0.187) | <0.001 | 0.182( 0.171, 0.193) | <0.001 | 0.177( 0.165, 0.188) | <0.001 |
| LV-SV | 0.206( 0.195, 0.216) | <0.001 | 0.214( 0.203, 0.225) | <0.001 | 0.21( 0.199, 0.221) | <0.001 |
| LV-EF | -0.043(-0.054,-0.032) | <0.001 | -0.043(-0.054,-0.031) | <0.001 | -0.043(-0.054,-0.031) | <0.001 |
| RV-EDV | 0.221( 0.211, 0.232) | <0.001 | 0.23( 0.219, 0.241) | <0.001 | 0.231( 0.220, 0.242) | <0.001 |
| RV-ESV | 0.197( 0.187, 0.208) | <0.001 | 0.204( 0.193, 0.215) | <0.001 | 0.208( 0.197, 0.219) | <0.001 |
| RV-SV | 0.199( 0.189, 0.210) | <0.001 | 0.209( 0.198, 0.220) | <0.001 | 0.206( 0.195, 0.217) | <0.001 |
| RV-EF | -0.076(-0.086,-0.065) | <0.001 | -0.075(-0.086,-0.063) | <0.001 | -0.082(-0.093,-0.070) | <0.001 |
| LAVmax | 0.225( 0.215, 0.236) | <0.001 | 0.236( 0.225, 0.247) | <0.001 | 0.221( 0.210, 0.233) | <0.001 |
| LAVmin | 0.18( 0.169,0.191) | <0.001 | 0.187( 0.176,0.198) | <0.001 | 0.168( 0.157, 0.179) | <0.001 |
| LA-SV | 0.213( 0.203, 0.224) | <0.001 | 0.226( 0.215, 0.237) | <0.001 | 0.221( 0.209, 0.232) | <0.001 |
| LA-EF | -0.07(-0.081,-0.060) | <0.001 | -0.073(-0.084,-0.062) | <0.001 | -0.059(-0.071,-0.048) | <0.001 |
| RAVmax | -0.011(-0.022,0.000) | 0.046 | -0.002(-0.013, 0.010) | 0.767 | 0(-0.011, 0.012) | 0.974 |
| RAVmin | -0.003(-0.013,0.008) | 0.643 | 0.007(-0.004,0.019) | 0.201 | 0.01(-0.002, 0.021) | 0.098 |
| RA-SV | -0.019(-0.030,-0.008) | <0.001 | -0.014(-0.025,-0.003) | 0.016 | -0.013(-0.025,-0.002) | 0.024 |
| RA-EF | -0.015(-0.026,-0.004) | 0.007 | -0.021(-0.033,-0.010) | <0.001 | -0.025(-0.037,-0.014) | <0.001 |

Models were adjusted by age, sex, race, education, income, physical activity, smoke, alcohol, DASH, PRS, and history of diabetes mellitus, hypertension, other CVD, and cancer. DASH: Dietary approaches to stop hypertension; PRS: Polygenic risk score; CVD: Cardiovascular diseases; CMR: Cardiac magnetic resonance imaging; LV-EDV: Left ventricularend diastolic volume; LV-ESV: Left ventricularend systolic volume; LV-SV: Left ventricularstroke volume; LV-EF: Left ventricularejection fraction; RV-EDV: Right ventricularend diastolic volume; RV-ESV: Right ventricularend systolic volume; RV-SV: Right ventricularstroke volume; RV-EF: Right ventricularejection fraction; LAVmax: Left atrial maximum volume; LAVmin: Left atrial minimum volume; LA-SV: Left atrial stroke volume; LA-EF: Left atrial ejection fraction; RAVmax: Right atrial maximum volume; RAVmin: Right atrial minimum volume; RA-SV: Right atrial stroke volume; RA-EF: Right atrial ejection fraction; TyG: Triglyceride-glucose index; TyG-BMI: TyG-body mass index.

**Table S9. Associations between TyG-WC and CMR indices.**

| CMR  (per 1SD) | Model 1 | | Model 2 | | Model 3 | |
| --- | --- | --- | --- | --- | --- | --- |
|  | β(95%CI) | *P* | β(95%CI) | *P* | β(95%CI) | *P* |
| LV-EDV | 0.344( 0.334, 0.355) | <0.001 | 0.356( 0.346, 0.367) | <0.001 | 0.357( 0.346, 0.367) | <0.001 |
| LV-ESV | 0.305( 0.294, 0.315) | <0.001 | 0.315( 0.304, 0.325) | <0.001 | 0.315( 0.304, 0.326) | <0.001 |
| LV-SV | 0.298( 0.288, 0.309) | <0.001 | 0.309( 0.299, 0.320) | <0.001 | 0.309( 0.298, 0.320) | <0.001 |
| LV-EF | -0.124(-0.135,-0.113) | <0.001 | -0.127(-0.138,-0.115) | <0.001 | -0.13(-0.141,-0.118) | <0.001 |
| RV-EDV | 0.362( 0.352, 0.372) | <0.001 | 0.375( 0.365, 0.386) | <0.001 | 0.383( 0.372, 0.394) | <0.001 |
| RV-ESV | 0.344( 0.334, 0.354) | <0.001 | 0.355( 0.345, 0.366) | <0.001 | 0.367( 0.356, 0.377) | <0.001 |
| RV-SV | 0.304( 0.294, 0.315) | <0.001 | 0.317( 0.306, 0.328) | <0.001 | 0.32( 0.309, 0.331) | <0.001 |
| RV-EF | -0.163(-0.174,-0.152) | <0.001 | -0.165(-0.177,-0.154) | <0.001 | -0.177(-0.188,-0.165) | <0.001 |
| LAVmax | 0.234( 0.223, 0.244) | <0.001 | 0.245( 0.234, 0.256) | <0.001 | 0.23( 0.218, 0.241) | <0.001 |
| LAVmin | 0.19( 0.180,0.201) | <0.001 | 0.198( 0.187,0.209) | <0.001 | 0.179( 0.167,0.190) | <0.001 |
| LA-SV | 0.216( 0.206, 0.227) | <0.001 | 0.229( 0.218, 0.240) | <0.001 | 0.223( 0.212, 0.234) | <0.001 |
| LA-EF | -0.076(-0.087,-0.065) | <0.001 | -0.079(-0.090,-0.068) | <0.001 | -0.065(-0.076,-0.053) | <0.001 |
| RAVmax | 0.105( 0.094,0.116) | <0.001 | 0.119( 0.107, 0.130) | <0.001 | 0.127( 0.115, 0.138) | <0.001 |
| RAVmin | 0.123(0.113,0.134) | <0.001 | 0.138( 0.126,0.149) | <0.001 | 0.146( 0.135, 0.158) | <0.001 |
| RA-SV | 0.043( 0.032,0.054) | <0.001 | 0.051( 0.039, 0.062) | <0.001 | 0.055( 0.043, 0.066) | <0.001 |
| RA-EF | -0.094(-0.105,-0.083) | <0.001 | -0.103(-0.114,-0.091) | <0.001 | -0.11(-0.121,-0.098) | <0.001 |

Models were adjusted by age, sex, race, education, income, physical activity, smoke, alcohol, DASH, PRS, and history of diabetes mellitus, hypertension, other CVD, and cancer. DASH: Dietary approaches to stop hypertension; PRS: Polygenic risk score; CVD: Cardiovascular diseases; CMR: Cardiac magnetic resonance imaging; LV-EDV: Left ventricularend diastolic volume; LV-ESV: Left ventricularend systolic volume; LV-SV: Left ventricularstroke volume; LV-EF: Left ventricularejection fraction; RV-EDV: Right ventricularend diastolic volume; RV-ESV: Right ventricularend systolic volume; RV-SV: Right ventricularstroke volume; RV-EF: Right ventricularejection fraction; LAVmax: Left atrial maximum volume; LAVmin: Left atrial minimum volume; LA-SV: Left atrial stroke volume; LA-EF: Left atrial ejection fraction; RAVmax: Right atrial maximum volume; RAVmin: Right atrial minimum volume; RA-SV: Right atrial stroke volume; RA-EF: Right atrial ejection fraction; TyG: Triglyceride-glucose index; TyG-WC.

**Table S10. Associations between TyG-WHtR and CMR indices.**

| CMR  (per 1SD) | Model 1 | | Model 2 | | Model 3 | |
| --- | --- | --- | --- | --- | --- | --- |
|  | β(95%CI) | *P* | β(95%CI) | *P* | β(95%CI) | *P* |
| LV-EDV | 0.182( 0.171, 0.192) | <0.001 | 0.188( 0.177, 0.199) | <0.001 | 0.18( 0.168, 0.191) | <0.001 |
| LV-ESV | 0.157( 0.146, 0.168) | <0.001 | 0.163( 0.152, 0.174) | <0.001 | 0.155( 0.144, 0.167) | <0.001 |
| LV-SV | 0.161( 0.150, 0.171) | <0.001 | 0.167( 0.156, 0.178) | <0.001 | 0.159( 0.148, 0.171) | <0.001 |
| LV-EF | -0.058(-0.069,-0.047) | <0.001 | -0.059(-0.070,-0.047) | <0.001 | -0.059(-0.070,-0.047) | <0.001 |
| RV-EDV | 0.192( 0.181, 0.202) | <0.001 | 0.2( 0.188, 0.211) | <0.001 | 0.198( 0.187, 0.210) | <0.001 |
| RV-ESV | 0.182( 0.171, 0.192) | <0.001 | 0.188( 0.177, 0.199) | <0.001 | 0.191( 0.179, 0.202) | <0.001 |
| RV-SV | 0.162( 0.151, 0.173) | <0.001 | 0.17( 0.158, 0.181) | <0.001 | 0.164( 0.153, 0.176) | <0.001 |
| RV-EF | -0.091(-0.102,-0.080) | <0.001 | -0.091(-0.102,-0.080) | <0.001 | -0.099(-0.110,-0.087) | <0.001 |
| LAVmax | 0.168( 0.157, 0.179) | <0.001 | 0.177( 0.166, 0.188) | <0.001 | 0.158( 0.146, 0.169) | <0.001 |
| LAVmin | 0.14( 0.129,0.151) | <0.001 | 0.146( 0.135,0.157) | <0.001 | 0.123( 0.112, 0.135) | <0.001 |
| LA-SV | 0.151( 0.140, 0.162) | <0.001 | 0.162( 0.150, 0.173) | <0.001 | 0.152( 0.141, 0.164) | <0.001 |
| LA-EF | -0.058(-0.069,-0.047) | <0.001 | -0.06(-0.072,-0.049) | <0.001 | -0.045(-0.056,-0.033) | <0.001 |
| RAVmax | -0.024(-0.035,-0.013) | <0.001 | -0.015(-0.026,-0.003) | 0.010 | -0.014(-0.025,-0.002) | 0.019 |
| RAVmin | -0.006(-0.017,0.005) | 0.281 | 0.004(-0.007,0.016) | 0.460 | 0.006(-0.006, 0.018) | 0.304 |
| RA-SV | -0.042(-0.053,-0.031) | <0.001 | -0.036(-0.048,-0.025) | <0.001 | -0.037(-0.049,-0.025) | <0.001 |
| RA-EF | -0.031(-0.042,-0.020) | <0.001 | -0.037(-0.048,-0.026) | <0.001 | -0.041(-0.053,-0.030) | <0.001 |

Models were adjusted by age, sex, race, education, income, physical activity, smoke, alcohol, DASH, PRS, and history of diabetes mellitus, hypertension, other CVD, and cancer. DASH: Dietary approaches to stop hypertension; PRS: Polygenic risk score; CVD: Cardiovascular diseases; CMR: Cardiac magnetic resonance imaging; LV-EDV: Left ventricularend diastolic volume; LV-ESV: Left ventricularend systolic volume; LV-SV: Left ventricularstroke volume; LV-EF: Left ventricularejection fraction; RV-EDV: Right ventricularend diastolic volume; RV-ESV: Right ventricularend systolic volume; RV-SV: Right ventricularstroke volume; RV-EF: Right ventricularejection fraction; LAVmax: Left atrial maximum volume; LAVmin: Left atrial minimum volume; LA-SV: Left atrial stroke volume; LA-EF: Left atrial ejection fraction; RAVmax: Right atrial maximum volume; RAVmin: Right atrial minimum volume; RA-SV: Right atrial stroke volume; RA-EF: Right atrial ejection fraction; TyG: Triglyceride-glucose index; TyG-WHtR: TyG-waist-to-height ratio.

**Table S11. Associations between obesity indicators and atrial fibrillation**

| TyG obesity-related derivatives | Model 1 | | Model 2 | | Model 3 | |
| --- | --- | --- | --- | --- | --- | --- |
|  | HR(95%CI) | *P* | HR(95%CI) | *P* | HR(95%CI) | *P* |
| BMI  (per 1SD) | 1.283(1.219,1.349) | <0.001 | 1.270(1.205,1.339) | <0.001 | 1.191(1.126,1.259) | <0.001 |
| Q1 | ref |  | ref |  | ref |  |
| Q2 | 0.968(0.808,1.160) | 0.727 | 0.989(0.822,1.189) | 0.904 | 0.943(0.784,1.134) | 0.534 |
| Q3 | 1.145(0.963,1.362) | 0.124 | 1.132(0.947,1.352) | 0.174 | 1.038(0.868,1.241) | 0.683 |
| Q4 | 1.763(1.499,2.073) | <0.001 | 1.748(1.477,2.068) | <0.001 | 1.478(1.244,1.756) | <0.001 |
| *P* for trend |  | <0.001 |  | <0.001 |  | <0.001 |
| WC  (per 1SD) | 1.354(1.277,1.436) | <0.001 | 1.335(1.255,1.420) | <0.001 | 1.242(1.164,1.325) | <0.001 |
| Q1 | ref |  | ref |  | ref |  |
| Q2 | 1.404(1.151,1.714) | <0.001 | 1.376(1.124,1.686) | 0.002 | 1.303(1.064,1.597) | 0.011 |
| Q3 | 1.560(1.269,1.916) | <0.001 | 1.510(1.223,1.864) | <0.001 | 1.376(1.114,1.700) | 0.003 |
| Q4 | 2.208(1.808,2.696) | <0.001 | 2.134(1.736,2.621) | <0.001 | 1.777(1.441,2.192) | <0.001 |
| *P* for trend |  | <0.001 |  | <0.001 |  | <0.001 |
| WHtR  (per 1SD) | 1.264(1.197,1.335) | <0.001 | 1.251(1.182,1.325) | <0.001 | 1.160(1.093,1.232) | <0.001 |
| Q1 | ref |  | ref |  | ref |  |
| Q2 | 1.056(0.876,1.274) | 0.565 | 1.020(0.843,1.234) | 0.840 | 0.977(0.807,1.182) | 0.810 |
| Q3 | 1.206(1.006,1.447) | 0.043 | 1.163(0.965,1.400) | 0.113 | 1.061(0.880,1.280) | 0.534 |
| Q4 | 1.641(1.378,1.953) | <0.001 | 1.588(1.327,1.902) | <0.001 | 1.318(1.096,1.585) | 0.003 |
| *P* for trend |  | <0.001 |  | <0.001 |  | <0.001 |

Model 1 was adjusted by age, sex, race, education, and income,

Model 2 was adjusted by Model 1 + physical activity, smoke, alcohol, DASH, and PRS

Model 3 was adjusted by Model 2 + history of diabetes mellitus, hypertension, other CVD, and cancer.

DASH: Dietary approaches to stop hypertension; PRS: Polygenic risk score; CVD: Cardiovascular diseases; BMI: TyG-body mass index; WC: TyG-waist circumference; WHtR: TyG-waist-to-height ratio.

**Table S12. Mediating roles of LA structure and function in the association between TyG obesity-related derivatives and atrial fibrillation.**

| LA | Effect | TyG-BMI | | TyG-WC | | TyG-WHtR | |
| --- | --- | --- | --- | --- | --- | --- | --- |
|  |  | HR(95%CI) | *P* | HR(95%CI) | *P* | HR(95%CI) | *P* |
| LAVmax | Total effect | 1.292(1.192,1.386) | <0.001 | 1.667(1.51,1.88) | <0.001 | 1.31(1.29,1.536) | <0.001 |
|  | Direct effect | 1.086(0.986,1.156) | 0.2 | 1.395(1.259,1.579) | <0.001 | 1.162(1.142,1.363) | <0.001 |
|  | Indirect effect | 1.19(1.186,1.21) | <0.001 | 1.195(1.188,1.206) | <0.001 | 1.128(1.121,1.144) | <0.001 |
|  | Mediation (%) | 70.63(59.45,108.71) | <0.001 | 40.79(33.92,49.38) | <0.001 | 47.83(32.22,51.59) | <0.001 |
| LAVmin | Total effect | 1.25(1.138,1.367) | <0.001 | 1.616(1.466,1.823) | <0.001 | 1.249(1.228,1.481) | <0.001 |
|  | Direct effect | 1.139(1.032,1.233) | <0.001 | 1.462(1.325,1.651) | <0.001 | 1.173(1.153,1.388) | <0.001 |
|  | Indirect effect | 1.098(1.096,1.112) | <0.001 | 1.105(1.102,1.114) | <0.001 | 1.065(1.062,1.073) | <0.001 |
|  | Mediation (%) | 44.5(36.52,79.3) | <0.001 | 25.02(20.84,30.39) | <0.001 | 30.57(19.46,32.7) | <0.001 |
| LA-SV | Total effect | 1.33(1.257,1.422) | <0.001 | 1.776(1.611,1.997) | <0.001 | 1.363(1.322,1.603) | <0.001 |
|  | Direct effect | 1.333(1.262,1.425) | <0.001 | 1.788(1.613,2.027) | <0.001 | 1.364(1.313,1.614) | <0.001 |
|  | Indirect effect | 0.998(0.986,1.014) | 0.8 | 0.993(0.981,1.009) | 0.6 | 0.999(0.99,1.01) | 0.8 |
|  | Mediation (%) | -0.88(-5.08,5.39) | 0.8 | -1.51(-3.82,1.92) | 0.6 | -0.22(-2.61,3.95) | 0.8 |
| LA-EF | Total effect | 1.253(1.169,1.315) | <0.001 | 1.633(1.505,1.824) | <0.001 | 1.272(1.239,1.48) | <0.001 |
|  | Direct effect | 1.234(1.143,1.301) | <0.001 | 1.598(1.478,1.768) | <0.001 | 1.283(1.258,1.487) | <0.001 |
|  | Indirect effect | 1.015(1.008,1.025) | <0.001 | 1.022(1.006,1.033) | <0.001 | 0.991(0.979,1.007) | 0.4 |
|  | Mediation (%) | 7.38(3.56,16.2) | <0.001 | 5.57(1.58,7.68) | <0.001 | -4.03(-8.97,2.61) | 0.4 |

Models were adjusted by age, sex, race, education, income, physical activity, smoke, alcohol, DASH, PRS, and history of diabetes mellitus, hypertension, other CVD, and cancer. DASH: Dietary approaches to stop hypertension; PRS: Polygenic risk score; CVD: Cardiovascular diseases; LAVmax: Left atrial maximum volume; LAVmin: Left atrial minimum volume; LA-SV: Left atrial stroke volume; LA-EF: Left atrial ejection fraction; TyG: Triglyceride-glucose index; TyG-BMI: TyG-body mass index; TyG-WC: TyG-waist circumference; TyG-WHtR: TyG-waist-to-height ratio; AF: atrial fibrillation.

**Table S13. Mediating roles of LV structure and function in the association between TyG obesity-related derivatives and atrial fibrillation.**

| LV | Effect | TyG-BMI | | TyG-WC | | TyG-WHtR | |
| --- | --- | --- | --- | --- | --- | --- | --- |
|  |  | HR(95%CI) | *P* | HR(95%CI) | *P* | HR(95%CI) | *P* |
| LV-EDV | Total effect | 1.317(1.238,1.41) | <0.001 | 1.741(1.579,1.971) | <0.001 | 1.35(1.306,1.594) | <0.001 |
|  | Direct effect | 1.159(1.08,1.241) | <0.001 | 1.433(1.29,1.661) | <0.001 | 1.205(1.152,1.437) | <0.001 |
|  | Indirect effect | 1.137(1.121,1.155) | <0.001 | 1.215(1.18,1.249) | <0.001 | 1.12(1.105,1.136) | <0.001 |
|  | Mediation (%) | 49.9(39.26,66.47) | <0.001 | 41.52(31.61,51.8) | <0.001 | 41.3(26.15,50.18) | <0.001 |
| LV-ESV | Total effect | 1.309(1.225,1.408) | <0.001 | 1.736(1.571,1.963) | <0.001 | 1.346(1.298,1.586) | <0.001 |
|  | Direct effect | 1.192(1.116,1.279) | <0.001 | 1.488(1.35,1.701) | <0.001 | 1.232(1.182,1.451) | <0.001 |
|  | Indirect effect | 1.098(1.091,1.122) | <0.001 | 1.167(1.149,1.209) | <0.001 | 1.093(1.086,1.116) | <0.001 |
|  | Mediation (%) | 37.7(30.42,48.59) | <0.001 | 33.7(27.04,39.56) | <0.001 | 32.99(22,38.99) | <0.001 |
| LV-SV | Total effect | 1.329(1.256,1.421) | <0.001 | 1.772(1.61,1.996) | <0.001 | 1.36(1.317,1.603) | <0.001 |
|  | Direct effect | 1.257(1.185,1.341) | <0.001 | 1.65(1.498,1.916) | <0.001 | 1.298(1.246,1.555) | <0.001 |
|  | Indirect effect | 1.058(1.034,1.071) | <0.001 | 1.074(1.035,1.097) | <0.001 | 1.048(1.028,1.058) | <0.001 |
|  | Mediation (%) | 22(11.96,29.5) | <0.001 | 15.87(6.87,21.72) | <0.001 | 17.14(7.41,22.64) | <0.001 |
| LV-EF | Total effect | 1.318(1.242,1.414) | <0.001 | 1.748(1.579,1.966) | <0.001 | 1.352(1.315,1.591) | <0.001 |
|  | Direct effect | 1.268(1.199,1.359) | <0.001 | 1.604(1.45,1.793) | <0.001 | 1.287(1.253,1.505) | <0.001 |
|  | Indirect effect | 1.04(1.035,1.053) | <0.001 | 1.09(1.077,1.1) | <0.001 | 1.051(1.045,1.06) | <0.001 |
|  | Mediation (%) | 15.74(12.07,19.86) | <0.001 | 19.2(15.78,22.3) | <0.001 | 18.49(13.18,19.73) | <0.001 |

Models were adjusted by age, sex, race, education, income, physical activity, smoke, alcohol, DASH, PRS, and history of diabetes mellitus, hypertension, other CVD, and cancer. DASH: Dietary approaches to stop hypertension; PRS: Polygenic risk score; CVD: Cardiovascular diseases; LV-EDV: Left ventricularend diastolic volume; LV-ESV: Left ventricularend systolic volume; LV-SV: Left ventricularstroke volume; LV-EF: Left ventricularejection fraction; TyG: Triglyceride-glucose index; TyG-BMI: TyG-body mass index; TyG-WC: TyG-waist circumference; TyG-WHtR: TyG-waist-to-height ratio; AF: atrial fibrillation.

**Table S14. Mediating roles of RA structure and function in the association between TyG obesity-related derivatives and atrial fibrillation.**

| RA | Effect | TyG-BMI | | TyG-WC | | TyG-WHtR | |
| --- | --- | --- | --- | --- | --- | --- | --- |
|  |  | HR(95%CI) | *P* | HR(95%CI) | *P* | HR(95%CI) | *P* |
| RAVmax | Total effect | 1.337(1.261,1.452) | <0.001 | 1.722(1.56,1.94) | <0.001 | 1.39(1.355,1.618) | <0.001 |
|  | Direct effect | 1.309(1.234,1.415) | <0.001 | 1.524(1.375,1.724) | <0.001 | 1.367(1.34,1.6) | <0.001 |
|  | Indirect effect | 1.022(1.014,1.033) | <0.001 | 1.13(1.119,1.146) | <0.001 | 1.017(1.009,1.027) | <0.001 |
|  | Mediation (%) | 8.37(5.07,11.71) | <0.001 | 27.51(22.7,33.96) | <0.001 | 5.91(2.45,8.6) | <0.001 |
| RAVmin | Total effect | 1.335(1.246,1.443) | <0.001 | 1.705(1.538,1.927) | <0.001 | 1.384(1.351,1.638) | <0.001 |
|  | Direct effect | 1.292(1.204,1.392) | <0.001 | 1.463(1.317,1.648) | <0.001 | 1.333(1.302,1.587) | <0.001 |
|  | Indirect effect | 1.033(1.024,1.05) | <0.001 | 1.166(1.153,1.18) | <0.001 | 1.038(1.031,1.049) | <0.001 |
|  | Mediation (%) | 12.69(8.69,17.87) | <0.001 | 34.39(29.74,41.98) | <0.001 | 13.23(8.05,15.29) | <0.001 |
| RA-SV | Total effect | 1.334(1.265,1.428) | <0.001 | 1.777(1.612,1.997) | <0.001 | 1.365(1.326,1.606) | <0.001 |
|  | Direct effect | 1.333(1.265,1.427) | <0.001 | 1.755(1.59,1.981) | <0.001 | 1.37(1.334,1.612) | <0.001 |
|  | Indirect effect | 1(0.999,1.002) | 0.8 | 1.013(1.007,1.015) | <0.001 | 0.997(0.992,0.999) | <0.001 |
|  | Mediation (%) | 0.2(-0.58,0.89) | 0.8 | 2.86(1.5,3.74) | <0.001 | -1.3(-3.11,-0.33) | <0.001 |
| RA-EF | Total effect | 1.328(1.241,1.412) | <0.001 | 1.768(1.588,2) | <0.001 | 1.357(1.319,1.6) | <0.001 |
|  | Direct effect | 1.283(1.196,1.358) | <0.001 | 1.584(1.432,1.768) | <0.001 | 1.285(1.256,1.507) | <0.001 |
|  | Indirect effect | 1.035(1.031,1.053) | <0.001 | 1.116(1.102,1.136) | <0.001 | 1.056(1.049,1.072) | <0.001 |
|  | Mediation (%) | 13.62(11.47,19.49) | <0.001 | 23.94(21.09,27.74) | <0.001 | 20.13(15.28,21.68) | <0.001 |

Models were adjusted by age, sex, race, education, income, physical activity, smoke, alcohol, DASH, PRS, and history of diabetes mellitus, hypertension, other CVD, and cancer. DASH: Dietary approaches to stop hypertension; PRS: Polygenic risk score; CVD: Cardiovascular diseases; RAVmax: Right atrial maximum volume; RAVmin: Right atrial minimum volume; RA-SV: Right atrial stroke volume; RA-EF: Right atrial ejection fraction; TyG: Triglyceride-glucose index; TyG-BMI: TyG-body mass index; TyG-WC: TyG-waist circumference; TyG-WHtR: TyG-waist-to-height ratio; AF: atrial fibrillation.

**Table S15. Mediating roles of RV structure and function in the association between TyG obesity-related derivatives and atrial fibrillation.**

| RV | Effect | TyG-BMI | | TyG-WC | | TyG-WHtR | |
| --- | --- | --- | --- | --- | --- | --- | --- |
|  |  | HR(95%CI) | *P* | HR(95%CI) | *P* | HR(95%CI) | *P* |
| RV-EDV | Total effect | 1.33(1.257,1.424) | <0.001 | 1.763(1.6,1.985) | <0.001 | 1.36(1.319,1.604) | <0.001 |
|  | Direct effect | 1.179(1.1,1.259) | <0.001 | 1.481(1.347,1.688) | <0.001 | 1.217(1.176,1.441) | <0.001 |
|  | Indirect effect | 1.128(1.12,1.144) | <0.001 | 1.19(1.176,1.213) | <0.001 | 1.117(1.109,1.13) | <0.001 |
|  | Mediation (%) | 45.7(38.87,61.14) | <0.001 | 36.91(30.13,43.32) | <0.001 | 39.61(27.03,44.79) | <0.001 |
| RV-ESV | Total effect | 1.327(1.257,1.422) | <0.001 | 1.741(1.581,1.956) | <0.001 | 1.358(1.318,1.6) | <0.001 |
|  | Direct effect | 1.14(1.065,1.223) | <0.001 | 1.372(1.245,1.545) | <0.001 | 1.174(1.145,1.381) | <0.001 |
|  | Indirect effect | 1.164(1.157,1.184) | <0.001 | 1.269(1.246,1.31) | <0.001 | 1.157(1.143,1.179) | <0.001 |
|  | Mediation (%) | 57.13(47.09,74.96) | <0.001 | 49.8(42.58,58.51) | <0.001 | 51.47(36.49,54.87) | <0.001 |
| RV-SV | Total effect | 1.331(1.257,1.423) | <0.001 | 1.777(1.611,1.998) | <0.001 | 1.363(1.321,1.605) | <0.001 |
|  | Direct effect | 1.29(1.216,1.377) | <0.001 | 1.721(1.567,1.965) | <0.001 | 1.325(1.27,1.572) | <0.001 |
|  | Indirect effect | 1.032(1.019,1.046) | <0.001 | 1.032(1.014,1.055) | <0.001 | 1.028(1.017,1.04) | <0.001 |
|  | Mediation (%) | 12.36(7.15,19.42) | <0.001 | 7.17(3.08,12.85) | <0.001 | 10.22(4.95,15.88) | <0.001 |
| RV-EF | Total effect | 1.323(1.262,1.413) | <0.001 | 1.752(1.587,1.961) | <0.001 | 1.352(1.31,1.584) | <0.001 |
|  | Direct effect | 1.247(1.19,1.335) | <0.001 | 1.576(1.429,1.756) | <0.001 | 1.265(1.241,1.481) | <0.001 |
|  | Indirect effect | 1.061(1.045,1.072) | <0.001 | 1.111(1.093,1.131) | <0.001 | 1.069(1.055,1.081) | <0.001 |
|  | Mediation (%) | 23.53(17.79,28.75) | <0.001 | 23.37(19.32,28.33) | <0.001 | 24.67(17.45,24.89) | <0.001 |

Models were adjusted by age, sex, race, education, income, physical activity, smoke, alcohol, DASH, PRS, and history of diabetes mellitus, hypertension, other CVD, and cancer. DASH: Dietary approaches to stop hypertension; PRS: Polygenic risk score; CVD: Cardiovascular diseases; RV-EDV: Right ventricularend diastolic volume; RV-ESV: Right ventricularend systolic volume; RV-SV: Right ventricularstroke volume; RV-EF: Right ventricularejection fraction; TyG: Triglyceride-glucose index; TyG-BMI: TyG-body mass index; TyG-WC: TyG-waist circumference; TyG-WHtR: TyG-waist-to-height ratio; AF: atrial fibrillation.

**Table S16. Sensitivity analysis of associations between TyG obesity-related derivatives and atrial fibrillation after excluding participants with missing data.**

| TyG obesity-related derivatives | Model 1 | | Model 2 | | Model 3 | |
| --- | --- | --- | --- | --- | --- | --- |
|  | HR(95%CI) | *P* | HR(95%CI) | *P* | HR(95%CI) | *P* |
| TyG  (per 1SD) | 1.051(0.984,1.122) | 0.139 | 1.046(0.979,1.117) | 0.180 | 1.037(0.970,1.108) | 0.290 |
| Q1 | ref |  | ref |  | ref |  |
| Q2 | 1.047(0.861,1.273) | 0.646 | 1.053(0.866,1.281) | 0.604 | 1.057(0.869,1.285) | 0.582 |
| Q3 | 1.052(0.868,1.275) | 0.607 | 1.044(0.861,1.266) | 0.661 | 1.038(0.856,1.259) | 0.704 |
| Q4 | 1.095(0.906,1.324) | 0.346 | 1.086(0.898,1.314) | 0.396 | 1.077(0.889,1.304) | 0.450 |
| *P* for trend |  | 0.362 |  | 0.441 |  | 0.518 |
| TyG-BMI  (per 1SD) | 1.243(1.170,1.321) | <0.001 | 1.237(1.162,1.316) | <0.001 | 1.223(1.147,1.304) | <0.001 |
| Q1 | ref |  | ref |  | ref |  |
| Q2 | 1.109(0.900,1.368) | 0.331 | 1.099(0.891,1.355) | 0.379 | 1.086(0.880,1.339) | 0.443 |
| Q3 | 1.174(0.956,1.443) | 0.126 | 1.143(0.929,1.406) | 0.205 | 1.126(0.916,1.385) | 0.260 |
| Q4 | 1.671(1.373,2.032) | <0.001 | 1.618(1.326,1.973) | <0.001 | 1.567(1.283,1.914) | <0.001 |
| *P* for trend |  | <0.001 |  | <0.001 |  | <0.001 |
| TyG-WC  (per 1SD) | 1.268(1.185,1.358) | <0.001 | 1.263(1.177,1.354) | <0.001 | 1.246(1.160,1.340) | <0.001 |
| Q1 | ref |  | ref |  | ref |  |
| Q2 | 1.338(1.054,1.699) | 0.017 | 1.324(1.042,1.682) | 0.021 | 1.303(1.026,1.656) | 0.030 |
| Q3 | 1.390(1.092,1.770) | 0.007 | 1.364(1.070,1.739) | 0.012 | 1.342(1.053,1.711) | 0.018 |
| Q4 | 1.913(1.508,2.426) | <0.001 | 1.855(1.458,2.359) | <0.001 | 1.793(1.407,2.284) | <0.001 |
| *P* for trend |  | <0.001 |  | <0.001 |  | <0.001 |
| TyG-WHtR  (per 1SD) | 1.221(1.145,1.303) | <0.001 | 1.219(1.141,1.303) | <0.001 | 1.202(1.123,1.287) | <0.001 |
| Q1 | ref |  | ref |  | ref |  |
| Q2 | 1.085(0.875,1.346) | 0.457 | 1.066(0.859,1.322) | 0.563 | 1.049(0.846,1.303) | 0.661 |
| Q3 | 1.145(0.925,1.417) | 0.213 | 1.129(0.911,1.398) | 0.268 | 1.105(0.891,1.369) | 0.364 |
| Q4 | 1.538(1.254,1.888) | <0.001 | 1.496(1.216,1.842) | <0.001 | 1.441(1.169,1.777) | <0.001 |
| *P* for trend |  | <0.001 |  | <0.001 |  | <0.001 |

n=24587

Model 1 was adjusted by age, sex, race, education, and income,

Model 2 was adjusted by Model 1 + physical activity, smoke, alcohol, DASH, and PRS

Model 3 was adjusted by Model 2 + history of diabetes mellitus, hypertension, other CVD, and cancer.

DASH: Dietary approaches to stop hypertension; PRS: Polygenic risk score; CVD: Cardiovascular diseases; TyG: Triglyceride-glucose index; TyG-BMI: TyG-body mass index; TyG-WC: TyG-waist circumference; TyG-WHtR: TyG-waist-to-height ratio.

**Table S17. Sensitivity analysis of associations between TyG obesity-related derivatives and atrial fibrillation after excluding participants who developed atrial fibrillation within the first two-year follow-up period.**

| TyG obesity-related derivatives | Model 1 | | Model 2 | | Model 3 | |
| --- | --- | --- | --- | --- | --- | --- |
|  | HR(95%CI) | *P* | HR(95%CI) | *P* | HR(95%CI) | *P* |
| TyG  (per 1SD) | 1.056(0.997,1.118) | 0.062 | 1.057(0.997,1.121) | 0.064 | 1.045(0.985,1.109) | 0.144 |
| Q1 | ref |  | ref |  | ref |  |
| Q2 | 1.004(0.847,1.192) | 0.959 | 0.989(0.830,1.178) | 0.904 | 0.992(0.833,1.182) | 0.928 |
| Q3 | 1.033(0.873,1.221) | 0.707 | 1.027(0.866,1.219) | 0.757 | 1.019(0.859,1.209) | 0.832 |
| Q4 | 1.085(0.920,1.281) | 0.331 | 1.086(0.917,1.285) | 0.341 | 1.069(0.902,1.267) | 0.440 |
| *P* for trend |  | 0.283 |  | 0.27 |  | 0.379 |
| TyG-BMI  (per 1SD) | 1.247(1.183,1.315) | <0.001 | 1.240(1.173,1.311) | <0.001 | 1.223(1.155,1.294) | <0.001 |
| Q1 | ref |  | ref |  | ref |  |
| Q2 | 1.054(0.877,1.266) | 0.578 | 1.036(0.859,1.250) | 0.712 | 1.025(0.850,1.237) | 0.795 |
| Q3 | 1.168(0.977,1.397) | 0.088 | 1.148(0.956,1.378) | 0.138 | 1.126(0.938,1.352) | 0.204 |
| Q4 | 1.620(1.367,1.921) | <0.001 | 1.595(1.339,1.901) | <0.001 | 1.539(1.290,1.837) | <0.001 |
| *P* for trend |  | <0.001 |  | <0.001 |  | <0.001 |
| TyG-WC  (per 1SD) | 1.276(1.202,1.355) | <0.001 | 1.265(1.188,1.346) | <0.001 | 1.245(1.167,1.327) | <0.001 |
| Q1 | ref |  | ref |  | ref |  |
| Q2 | 1.292(1.056,1.580) | 0.013 | 1.280(1.042,1.571) | 0.019 | 1.260(1.026,1.547) | 0.028 |
| Q3 | 1.337(1.090,1.640) | 0.005 | 1.325(1.075,1.633) | 0.008 | 1.301(1.056,1.604) | 0.014 |
| Q4 | 1.830(1.496,2.238) | <0.001 | 1.804(1.466,2.220) | <0.001 | 1.733(1.405,2.136) | <0.001 |
| *P* for trend |  | <0.001 |  | <0.001 |  | <0.001 |
| TyG-WHtR  (per 1SD) | 1.218(1.151,1.288) | <0.001 | 1.210(1.140,1.283) | <0.001 | 1.188(1.119,1.263) | <0.001 |
| Q1 | ref |  | ref |  | ref |  |
| Q2 | 1.026(0.849,1.239) | 0.789 | 1.013(0.836,1.228) | 0.894 | 0.998(0.823,1.209) | 0.980 |
| Q3 | 1.209(1.007,1.451) | 0.042 | 1.188(0.985,1.433) | 0.072 | 1.162(0.963,1.402) | 0.116 |
| Q4 | 1.467(1.228,1.754) | <0.001 | 1.444(1.201,1.736) | <0.001 | 1.381(1.147,1.663) | <0.001 |
| *P* for trend |  | <0.001 |  | <0.001 |  | <0.001 |

n=32437

Model 1 was adjusted by age, sex, race, education, and income,

Model 2 was adjusted by Model 1 + physical activity, smoke, alcohol, DASH, and PRS

Model 3 was adjusted by Model 2 + history of diabetes mellitus, hypertension, other CVD, and cancer.

DASH: Dietary approaches to stop hypertension; PRS: Polygenic risk score; CVD: Cardiovascular diseases; TyG: Triglyceride-glucose index; TyG-BMI: TyG-body mass index; TyG-WC: TyG-waist circumference; TyG-WHtR: TyG-waist-to-height ratio.

**Table S18. Sensitivity analysis of associations between TyG obesity-related derivatives and atrial fibrillation after excluding participants with a history of CVD.**

| TyG obesity-related derivatives | Model 1 | | Model 2 | | Model 3 | |
| --- | --- | --- | --- | --- | --- | --- |
|  | HR(95%CI) | *P* | HR(95%CI) | *P* | HR(95%CI) | *P* |
| TyG  (per 1SD) | 1.057(0.997,1.121) | 0.064 | 1.059(0.997,1.125) | 0.063 | 1.049(0.987,1.115) | 0.121 |
| Q1 | ref |  | ref |  | ref |  |
| Q2 | 1.024(0.859,1.221) | 0.790 | 1.000(0.835,1.198) | 0.998 | 0.997(0.833,1.194) | 0.977 |
| Q3 | 1.065(0.896,1.266) | 0.474 | 1.063(0.892,1.268) | 0.493 | 1.057(0.887,1.261) | 0.534 |
| Q4 | 1.119(0.943,1.327) | 0.197 | 1.111(0.933,1.323) | 0.237 | 1.091(0.916,1.300) | 0.328 |
| *P* for trend |  | 0.162 |  | 0.167 |  | 0.243 |
| TyG-BMI  (per 1SD) | 1.240(1.174,1.310) | <0.001 | 1.232(1.164,1.305) | <0.001 | 1.225(1.156,1.299) | <0.001 |
| Q1 | ref |  | ref |  | ref |  |
| Q2 | 1.094(0.907,1.319) | 0.347 | 1.079(0.891,1.305) | 0.437 | 1.076(0.889,1.302) | 0.452 |
| Q3 | 1.177(0.979,1.414) | 0.082 | 1.157(0.959,1.396) | 0.128 | 1.151(0.954,1.389) | 0.142 |
| Q4 | 1.643(1.380,1.956) | <0.001 | 1.620(1.353,1.939) | <0.001 | 1.598(1.334,1.915) | <0.001 |
| *P* for trend |  | <0.001 |  | <0.001 |  | <0.001 |
| TyG-WC  (per 1SD) | 1.274(1.198,1.355) | <0.001 | 1.263(1.184,1.348) | <0.001 | 1.255(1.175,1.340) | <0.001 |
| Q1 | ref |  | ref |  | ref |  |
| Q2 | 1.272(1.037,1.559) | 0.021 | 1.262(1.025,1.554) | 0.028 | 1.256(1.020,1.546) | 0.031 |
| Q3 | 1.338(1.088,1.645) | 0.006 | 1.325(1.072,1.637) | 0.009 | 1.315(1.064,1.625) | 0.011 |
| Q4 | 1.819(1.483,2.231) | <0.001 | 1.795(1.454,2.217) | <0.001 | 1.764(1.428,2.180) | <0.001 |
| *P* for trend |  | <0.001 |  | <0.001 |  | <0.001 |
| TyG-WHtR  (per 1SD) | 1.209(1.141,1.282) | <0.001 | 1.204(1.133,1.279) | <0.001 | 1.195(1.123,1.271) | <0.001 |
| Q1 | ref |  | ref |  | ref |  |
| Q2 | 1.042(0.861,1.261) | 0.674 | 1.031(0.849,1.252) | 0.760 | 1.027(0.846,1.248) | 0.785 |
| Q3 | 1.200(0.997,1.446) | 0.054 | 1.180(0.975,1.428) | 0.089 | 1.174(0.971,1.421) | 0.098 |
| Q4 | 1.469(1.225,1.761) | <0.001 | 1.448(1.200,1.747) | <0.001 | 1.422(1.178,1.718) | <0.001 |
| *P* for trend |  | <0.001 |  | <0.001 |  | <0.001 |

n=31333

Model 1 was adjusted by age, sex, race, education, and income,

Model 2 was adjusted by Model 1 + physical activity, smoke, alcohol, DASH, and PRS

Model 3 was adjusted by Model 2 + history of diabetes mellitus, hypertension, and cancer.

DASH: Dietary approaches to stop hypertension; PRS: Polygenic risk score; CVD: Cardiovascular diseases; TyG: Triglyceride-glucose index; TyG-BMI: TyG-body mass index; TyG-WC: TyG-waist circumference; TyG-WHtR: TyG-waist-to-height ratio.

**Table S19. Sensitivity analysis of mediating roles of cardiac structure and function in the association between TyG obesity-related derivatives and atrial fibrillation after excluding participants with missing data.**

| CMR | Effect | TyG-BMI | | TyG-WC | | TyG-WHtR | |
| --- | --- | --- | --- | --- | --- | --- | --- |
|  |  | HR(95%CI) | *P* | HR(95%CI) | *P* | HR(95%CI) | *P* |
| LV-EDV | Total effect | 1.354(1.258,1.563) | <0.001 | 1.86(1.678,2.197) | <0.001 | 1.391(1.288,1.661) | <0.001 |
|  | Direct effect | 1.185(1.095,1.361) | <0.001 | 1.513(1.356,1.793) | <0.001 | 1.236(1.137,1.465) | <0.001 |
|  | Indirect effect | 1.142(1.133,1.155) | <0.001 | 1.229(1.215,1.26) | <0.001 | 1.125(1.116,1.139) | <0.001 |
|  | Mediation (%) | 47.73(35.57,63.55) | <0.001 | 40.34(33.38,48.88) | <0.001 | 39.54(29.77,52.53) | <0.001 |
| LV-ESV | Total effect | 1.346(1.252,1.558) | <0.001 | 1.858(1.675,2.192) | <0.001 | 1.388(1.281,1.667) | <0.001 |
|  | Direct effect | 1.224(1.127,1.405) | <0.001 | 1.585(1.428,1.9) | <0.001 | 1.268(1.165,1.507) | <0.001 |
|  | Indirect effect | 1.099(1.087,1.118) | <0.001 | 1.172(1.148,1.205) | <0.001 | 1.095(1.082,1.113) | <0.001 |
|  | Mediation (%) | 35.18(26.86,49.67) | <0.001 | 31.78(24.38,38.41) | <0.001 | 30.89(22.92,41.35) | <0.001 |
| LV-SV | Total effect | 1.364(1.273,1.581) | <0.001 | 1.894(1.71,2.241) | <0.001 | 1.396(1.287,1.677) | <0.001 |
|  | Direct effect | 1.287(1.199,1.475) | <0.001 | 1.755(1.578,2.044) | <0.001 | 1.331(1.22,1.583) | <0.001 |
|  | Indirect effect | 1.06(1.045,1.075) | <0.001 | 1.079(1.059,1.103) | <0.001 | 1.049(1.038,1.064) | <0.001 |
|  | Mediation (%) | 21.11(13.22,28.63) | <0.001 | 15.51(10.99,20.77) | <0.001 | 16.45(10.81,23.29) | <0.001 |
| LV-EF | Total effect | 1.353(1.257,1.58) | <0.001 | 1.867(1.686,2.222) | <0.001 | 1.389(1.274,1.684) | <0.001 |
|  | Direct effect | 1.296(1.198,1.516) | <0.001 | 1.702(1.535,2.042) | <0.001 | 1.314(1.202,1.602) | <0.001 |
|  | Indirect effect | 1.045(1.034,1.054) | <0.001 | 1.097(1.086,1.116) | <0.001 | 1.057(1.047,1.068) | <0.001 |
|  | Mediation (%) | 16.34(10.54,22.87) | <0.001 | 19.09(14.45,23.07) | <0.001 | 19.26(12.04,26.54) | <0.001 |
| RV-EDV | Total effect | 1.367(1.275,1.587) | <0.001 | 1.882(1.696,2.232) | <0.001 | 1.397(1.286,1.678) | <0.001 |
|  | Direct effect | 1.203(1.121,1.384) | <0.001 | 1.559(1.395,1.842) | <0.001 | 1.242(1.136,1.478) | <0.001 |
|  | Indirect effect | 1.136(1.111,1.155) | <0.001 | 1.208(1.168,1.247) | <0.001 | 1.125(1.098,1.145) | <0.001 |
|  | Mediation (%) | 44.58(30.32,56.89) | <0.001 | 36.69(29.73,45.12) | <0.001 | 39.15(26.97,52.42) | <0.001 |
| RV-ESV | Total effect | 1.367(1.273,1.582) | <0.001 | 1.854(1.671,2.196) | <0.001 | 1.398(1.289,1.67) | <0.001 |
|  | Direct effect | 1.161(1.068,1.335) | <0.001 | 1.428(1.284,1.708) | <0.001 | 1.193(1.1,1.422) | <0.001 |
|  | Indirect effect | 1.177(1.151,1.196) | <0.001 | 1.298(1.254,1.335) | <0.001 | 1.172(1.145,1.189) | <0.001 |
|  | Mediation (%) | 55.99(41.84,75.14) | <0.001 | 49.91(39.77,57.9) | <0.001 | 51.42(35.54,65.3) | <0.001 |
| RV-SV | Total effect | 1.366(1.274,1.586) | <0.001 | 1.899(1.713,2.252) | <0.001 | 1.398(1.284,1.684) | <0.001 |
|  | Direct effect | 1.324(1.227,1.525) | <0.001 | 1.841(1.642,2.154) | <0.001 | 1.361(1.239,1.625) | <0.001 |
|  | Indirect effect | 1.031(1.008,1.048) | <0.001 | 1.032(0.997,1.061) | 0.4 | 1.028(1.008,1.042) | <0.001 |
|  | Mediation (%) | 11.3(2.7,19.67) | <0.001 | 6.5(-0.51,13.32) | 0.4 | 9.41(2.43,16.9) | <0.001 |
| RV-EF | Total effect | 1.356(1.263,1.584) | <0.001 | 1.862(1.682,2.215) | <0.001 | 1.384(1.272,1.679) | <0.001 |
|  | Direct effect | 1.272(1.165,1.488) | <0.001 | 1.66(1.494,1.983) | <0.001 | 1.285(1.186,1.571) | <0.001 |
|  | Indirect effect | 1.066(1.057,1.091) | <0.001 | 1.121(1.104,1.151) | <0.001 | 1.077(1.064,1.096) | <0.001 |
|  | Mediation (%) | 23.57(16.37,39.02) | <0.001 | 23.39(18.64,28.21) | <0.001 | 25.71(15.98,31.79) | <0.001 |
| LAVmax | Total effect | 1.331(1.209,1.541) | <0.001 | 1.774(1.568,2.115) | <0.001 | 1.346(1.215,1.586) | <0.001 |
|  | Direct effect | 1.126(1.02,1.307) | <0.001 | 1.49(1.308,1.772) | <0.001 | 1.201(1.084,1.408) | <0.001 |
|  | Indirect effect | 1.182(1.161,1.197) | <0.001 | 1.191(1.174,1.213) | <0.001 | 1.121(1.108,1.134) | <0.001 |
|  | Mediation (%) | 61.94(43.14,90.78) | <0.001 | 36.74(30.83,45.71) | <0.001 | 41.88(29.99,61.34) | <0.001 |
| LAVmin | Total effect | 1.271(1.164,1.495) | <0.001 | 1.713(1.512,2.076) | <0.001 | 1.278(1.162,1.515) | <0.001 |
|  | Direct effect | 1.162(1.065,1.363) | <0.001 | 1.551(1.369,1.867) | <0.001 | 1.204(1.098,1.428) | <0.001 |
|  | Indirect effect | 1.094(1.08,1.112) | <0.001 | 1.105(1.091,1.124) | <0.001 | 1.062(1.053,1.077) | <0.001 |
|  | Mediation (%) | 40.12(25.62,63.59) | <0.001 | 22.74(18.17,28.51) | <0.001 | 26.82(16.56,39.71) | <0.001 |
| LA-SV | Total effect | 1.365(1.272,1.586) | <0.001 | 1.899(1.714,2.251) | <0.001 | 1.398(1.284,1.687) | <0.001 |
|  | Direct effect | 1.371(1.275,1.58) | <0.001 | 1.916(1.725,2.255) | <0.001 | 1.401(1.28,1.681) | <0.001 |
|  | Indirect effect | 0.996(0.982,1.006) | 1 | 0.991(0.979,1.001) | 0.6 | 0.998(0.989,1.005) | 1 |
|  | Mediation (%) | -1.67(-6.75,2.46) | 1 | -1.91(-4.84,0.14) | 0.6 | -0.71(-3.32,1.86) | 1 |
| LA-EF | Total effect | 1.284(1.19,1.533) | <0.001 | 1.739(1.533,2.138) | <0.001 | 1.307(1.191,1.604) | <0.001 |
|  | Direct effect | 1.264(1.166,1.524) | <0.001 | 1.691(1.481,2.086) | <0.001 | 1.313(1.204,1.627) | <0.001 |
|  | Indirect effect | 1.016(0.999,1.025) | 0.2 | 1.028(1.013,1.048) | <0.001 | 0.996(0.983,1.009) | 0.8 |
|  | Mediation (%) | 7.07(-0.57,14.15) | 0.2 | 6.46(3.03,11.87) | <0.001 | -1.83(-9.67,2.84) | 0.8 |
| RAVmax | Total effect | 1.377(1.275,1.599) | <0.001 | 1.844(1.645,2.195) | <0.001 | 1.442(1.33,1.731) | <0.001 |
|  | Direct effect | 1.35(1.247,1.56) | <0.001 | 1.622(1.436,1.942) | <0.001 | 1.42(1.316,1.705) | <0.001 |
|  | Indirect effect | 1.02(1.015,1.031) | <0.001 | 1.137(1.121,1.146) | <0.001 | 1.016(1.01,1.02) | <0.001 |
|  | Mediation (%) | 7.25(4.95,10.58) | <0.001 | 26.28(21.15,32.41) | <0.001 | 5.04(2.72,5.96) | <0.001 |
| RAVmin | Total effect | 1.37(1.274,1.589) | <0.001 | 1.823(1.607,2.187) | <0.001 | 1.432(1.321,1.701) | <0.001 |
|  | Direct effect | 1.327(1.234,1.529) | <0.001 | 1.55(1.339,1.875) | <0.001 | 1.378(1.271,1.642) | <0.001 |
|  | Indirect effect | 1.032(1.021,1.051) | <0.001 | 1.176(1.157,1.202) | <0.001 | 1.039(1.03,1.047) | <0.001 |
|  | Mediation (%) | 11.63(7.05,15.2) | <0.001 | 33.2(26.3,44.19) | <0.001 | 12.43(7.81,15.76) | <0.001 |
| RA-SV | Total effect | 1.37(1.279,1.591) | <0.001 | 1.9(1.716,2.254) | <0.001 | 1.403(1.291,1.692) | <0.001 |
|  | Direct effect | 1.37(1.278,1.59) | <0.001 | 1.877(1.696,2.233) | <0.001 | 1.409(1.3,1.695) | <0.001 |
|  | Indirect effect | 1(0.999,1.002) | 0.8 | 1.012(1.005,1.017) | <0.001 | 0.996(0.992,0.998) | <0.001 |
|  | Mediation (%) | -0.01(-0.29,0.66) | 0.8 | 2.58(1.05,3.79) | <0.001 | -1.49(-3.22,-0.52) | <0.001 |
| RA-EF | Total effect | 1.357(1.261,1.578) | <0.001 | 1.886(1.682,2.24) | <0.001 | 1.392(1.278,1.677) | <0.001 |
|  | Direct effect | 1.309(1.22,1.524) | <0.001 | 1.673(1.459,1.989) | <0.001 | 1.311(1.209,1.581) | <0.001 |
|  | Indirect effect | 1.037(1.023,1.063) | <0.001 | 1.127(1.112,1.168) | <0.001 | 1.062(1.055,1.084) | <0.001 |
|  | Mediation (%) | 13.58(7.65,18.09) | <0.001 | 24.03(19.35,34.32) | <0.001 | 20.69(14.2,26.41) | <0.001 |

n=24587.

Models were adjusted by age, sex, race, education, income, physical activity, smoke, alcohol, DASH, PRS, and history of diabetes mellitus, hypertension, other CVD, and cancer. DASH: Dietary approaches to stop hypertension; PRS: Polygenic risk score; CVD: Cardiovascular diseases; CMR: Cardiac magnetic resonance imaging; LV-EDV: Left ventricularend diastolic volume; LV-ESV: Left ventricularend systolic volume; LV-SV: Left ventricularstroke volume; LV-EF: Left ventricularejection fraction; RV-EDV: Right ventricularend diastolic volume; RV-ESV: Right ventricularend systolic volume; RV-SV: Right ventricularstroke volume; RV-EF: Right ventricularejection fraction; LAVmax: Left atrial maximum volume; LAVmin: Left atrial minimum volume; LA-SV: Left atrial stroke volume; LA-EF: Left atrial ejection fraction; RAVmax: Right atrial maximum volume; RAVmin: Right atrial minimum volume; RA-SV: Right atrial stroke volume; RA-EF: Right atrial ejection fraction; TyG: Triglyceride-glucose index; TyG-BMI: TyG-body mass index; TyG-WC: TyG-waist circumference; TyG-WHtR: TyG-waist-to-height ratio.

**Table S20. Sensitivity analysis of mediating roles of cardiac structure and function in the association between TyG obesity-related derivatives and atrial fibrillation after excluding participants who developed atrial fibrillation within the first two-year follow-up period.**

| CMR | Effect | TyG-BMI | | TyG-WC | | TyG-WHtR | |
| --- | --- | --- | --- | --- | --- | --- | --- |
|  |  | HR(95%CI) | *P* | HR(95%CI) | *P* | HR(95%CI) | *P* |
| LV-EDV | Total effect | 1.294(1.161,1.474) | <0.001 | 1.699(1.603,1.799) | <0.001 | 1.326(1.197,1.506) | <0.001 |
|  | Direct effect | 1.136(1.007,1.288) | <0.001 | 1.393(1.304,1.49) | <0.001 | 1.182(1.054,1.341) | <0.001 |
|  | Indirect effect | 1.139(1.134,1.159) | <0.001 | 1.22(1.206,1.257) | <0.001 | 1.122(1.112,1.141) | <0.001 |
|  | Mediation (%) | 53.74(39.69,95.61) | <0.001 | 43.77(38.5,50.92) | <0.001 | 44.22(32.77,74.28) | <0.001 |
| LV-ESV | Total effect | 1.286(1.156,1.469) | <0.001 | 1.694(1.593,1.798) | <0.001 | 1.323(1.195,1.503) | <0.001 |
|  | Direct effect | 1.171(1.041,1.339) | <0.001 | 1.451(1.334,1.566) | <0.001 | 1.21(1.085,1.381) | <0.001 |
|  | Indirect effect | 1.098(1.089,1.12) | <0.001 | 1.168(1.148,1.21) | <0.001 | 1.093(1.081,1.118) | <0.001 |
|  | Mediation (%) | 40.26(28.33,74.51) | <0.001 | 35.08(29.02,45.15) | <0.001 | 34.99(24.35,57.38) | <0.001 |
| LV-SV | Total effect | 1.306(1.168,1.489) | <0.001 | 1.73(1.632,1.829) | <0.001 | 1.336(1.199,1.521) | <0.001 |
|  | Direct effect | 1.232(1.089,1.389) | <0.001 | 1.605(1.512,1.717) | <0.001 | 1.274(1.134,1.435) | <0.001 |
|  | Indirect effect | 1.06(1.049,1.074) | <0.001 | 1.078(1.059,1.098) | <0.001 | 1.049(1.039,1.061) | <0.001 |
|  | Mediation (%) | 24.06(20.29,46.91) | <0.001 | 17.12(12.96,20.26) | <0.001 | 18.63(15.76,33.76) | <0.001 |
| LV-EF | Total effect | 1.296(1.16,1.482) | <0.001 | 1.707(1.606,1.81) | <0.001 | 1.329(1.198,1.515) | <0.001 |
|  | Direct effect | 1.246(1.11,1.422) | <0.001 | 1.566(1.463,1.676) | <0.001 | 1.265(1.145,1.449) | <0.001 |
|  | Indirect effect | 1.04(1.033,1.054) | <0.001 | 1.09(1.077,1.101) | <0.001 | 1.051(1.042,1.065) | <0.001 |
|  | Mediation (%) | 16.73(12.27,33.25) | <0.001 | 19.91(16.51,23.65) | <0.001 | 19.56(12.85,28.23) | <0.001 |
| RV-EDV | Total effect | 1.307(1.171,1.49) | <0.001 | 1.722(1.623,1.822) | <0.001 | 1.337(1.202,1.521) | <0.001 |
|  | Direct effect | 1.159(1.03,1.32) | <0.001 | 1.445(1.351,1.569) | <0.001 | 1.196(1.065,1.365) | <0.001 |
|  | Indirect effect | 1.128(1.113,1.143) | <0.001 | 1.191(1.161,1.208) | <0.001 | 1.117(1.1,1.129) | <0.001 |
|  | Mediation (%) | 48.32(34.67,82.34) | <0.001 | 38.29(30.67,43.62) | <0.001 | 41.68(29.92,69.85) | <0.001 |
| RV-ESV | Total effect | 1.304(1.166,1.488) | <0.001 | 1.701(1.603,1.802) | <0.001 | 1.335(1.2,1.517) | <0.001 |
|  | Direct effect | 1.122(0.996,1.279) | 0.4 | 1.342(1.253,1.448) | <0.001 | 1.155(1.028,1.318) | <0.001 |
|  | Indirect effect | 1.163(1.151,1.185) | <0.001 | 1.268(1.243,1.299) | <0.001 | 1.156(1.141,1.175) | <0.001 |
|  | Mediation (%) | 59.95(42.98,102.4) | <0.001 | 51.24(44.13,58.13) | <0.001 | 53.8(38.53,88.17) | <0.001 |
| RV-SV | Total effect | 1.308(1.17,1.491) | <0.001 | 1.735(1.636,1.835) | <0.001 | 1.339(1.202,1.525) | <0.001 |
|  | Direct effect | 1.266(1.126,1.441) | <0.001 | 1.677(1.576,1.805) | <0.001 | 1.301(1.163,1.479) | <0.001 |
|  | Indirect effect | 1.033(1.017,1.046) | <0.001 | 1.035(1.009,1.052) | <0.001 | 1.029(1.015,1.038) | <0.001 |
|  | Mediation (%) | 13.66(7.83,27.71) | <0.001 | 7.99(2.05,11.72) | <0.001 | 11.21(6.58,20.27) | <0.001 |
| RV-EF | Total effect | 1.3(1.154,1.49) | <0.001 | 1.711(1.612,1.812) | <0.001 | 1.329(1.193,1.516) | <0.001 |
|  | Direct effect | 1.226(1.086,1.393) | <0.001 | 1.541(1.458,1.629) | <0.001 | 1.244(1.117,1.415) | <0.001 |
|  | Indirect effect | 1.06(1.055,1.071) | <0.001 | 1.11(1.097,1.12) | <0.001 | 1.068(1.059,1.078) | <0.001 |
|  | Mediation (%) | 24.62(19.64,44.35) | <0.001 | 23.93(21.92,25.98) | <0.001 | 25.76(18.19,40.27) | <0.001 |
| LAVmax | Total effect | 1.27(1.135,1.43) | <0.001 | 1.629(1.533,1.749) | <0.001 | 1.289(1.171,1.461) | <0.001 |
|  | Direct effect | 1.068(0.949,1.209) | 0.6 | 1.364(1.289,1.481) | <0.001 | 1.143(1.032,1.296) | <0.001 |
|  | Indirect effect | 1.19(1.179,1.209) | <0.001 | 1.194(1.177,1.215) | <0.001 | 1.128(1.117,1.142) | <0.001 |
|  | Mediation (%) | 74.98(52.42,137.48) | <0.001 | 42.13(35.77,48.11) | <0.001 | 50.47(35.93,83.29) | <0.001 |
| LAVmin | Total effect | 1.229(1.072,1.402) | <0.001 | 1.581(1.476,1.727) | <0.001 | 1.23(1.109,1.408) | <0.001 |
|  | Direct effect | 1.12(0.977,1.277) | 0.2 | 1.431(1.341,1.561) | <0.001 | 1.155(1.035,1.319) | <0.001 |
|  | Indirect effect | 1.097(1.085,1.103) | <0.001 | 1.104(1.092,1.116) | <0.001 | 1.065(1.058,1.074) | <0.001 |
|  | Mediation (%) | 47.6(31.47,140.44) | <0.001 | 25.75(21.63,30.22) | <0.001 | 32.62(22.2,70.07) | <0.001 |
| LA-SV | Total effect | 1.307(1.169,1.491) | <0.001 | 1.735(1.635,1.835) | <0.001 | 1.339(1.202,1.526) | <0.001 |
|  | Direct effect | 1.304(1.15,1.486) | <0.001 | 1.738(1.639,1.841) | <0.001 | 1.336(1.196,1.525) | <0.001 |
|  | Indirect effect | 1.003(0.998,1.02) | 0.6 | 0.998(0.994,1.015) | 0.6 | 1.003(0.999,1.014) | 0.2 |
|  | Mediation (%) | 1.08(-0.87,12.61) | 0.6 | -0.44(-1.47,3.36) | 0.6 | 1.04(-0.28,6.03) | 0.2 |
| LA-EF | Total effect | 1.232(1.09,1.407) | <0.001 | 1.597(1.493,1.743) | <0.001 | 1.252(1.111,1.423) | <0.001 |
|  | Direct effect | 1.215(1.071,1.377) | <0.001 | 1.564(1.466,1.705) | <0.001 | 1.263(1.123,1.427) | <0.001 |
|  | Indirect effect | 1.014(1.003,1.022) | <0.001 | 1.021(1.01,1.031) | <0.001 | 0.991(0.987,1.004) | 0.2 |
|  | Mediation (%) | 7.53(1.76,20.82) | <0.001 | 5.46(2.72,7.9) | <0.001 | -4.38(-13.74,1.72) | 0.2 |
| RAVmax | Total effect | 1.314(1.173,1.501) | <0.001 | 1.684(1.582,1.793) | <0.001 | 1.365(1.228,1.549) | <0.001 |
|  | Direct effect | 1.287(1.153,1.458) | <0.001 | 1.492(1.394,1.599) | <0.001 | 1.343(1.206,1.517) | <0.001 |
|  | Indirect effect | 1.021(1.015,1.031) | <0.001 | 1.128(1.118,1.141) | <0.001 | 1.017(1.013,1.025) | <0.001 |
|  | Mediation (%) | 8.76(8.55,17.33) | <0.001 | 28.02(24.4,32.32) | <0.001 | 6.17(5,9.93) | <0.001 |
| RAVmin | Total effect | 1.311(1.171,1.492) | <0.001 | 1.668(1.572,1.789) | <0.001 | 1.359(1.211,1.541) | <0.001 |
|  | Direct effect | 1.269(1.134,1.433) | <0.001 | 1.433(1.35,1.543) | <0.001 | 1.309(1.163,1.481) | <0.001 |
|  | Indirect effect | 1.033(1.027,1.044) | <0.001 | 1.164(1.155,1.174) | <0.001 | 1.038(1.031,1.046) | <0.001 |
|  | Mediation (%) | 13.45(11.65,25.38) | <0.001 | 35.21(30.92,39.18) | <0.001 | 13.95(10.73,23.27) | <0.001 |
| RA-SV | Total effect | 1.311(1.172,1.498) | <0.001 | 1.736(1.636,1.837) | <0.001 | 1.342(1.203,1.53) | <0.001 |
|  | Direct effect | 1.31(1.173,1.494) | <0.001 | 1.713(1.61,1.812) | <0.001 | 1.347(1.206,1.535) | <0.001 |
|  | Indirect effect | 1(0.999,1.004) | 0.4 | 1.013(1.007,1.021) | <0.001 | 0.996(0.995,0.999) | <0.001 |
|  | Mediation (%) | 0.19(-1,1.96) | 0.4 | 3.04(1.78,4.82) | <0.001 | -1.42(-2.26,-0.45) | <0.001 |
| RA-EF | Total effect | 1.306(1.167,1.478) | <0.001 | 1.728(1.617,1.83) | <0.001 | 1.335(1.193,1.509) | <0.001 |
|  | Direct effect | 1.263(1.122,1.431) | <0.001 | 1.551(1.448,1.657) | <0.001 | 1.265(1.126,1.444) | <0.001 |
|  | Indirect effect | 1.034(1.023,1.046) | <0.001 | 1.114(1.093,1.129) | <0.001 | 1.055(1.041,1.067) | <0.001 |
|  | Mediation (%) | 14.17(9.1,26.58) | <0.001 | 24.29(19.23,27.96) | <0.001 | 20.9(12.87,36.02) | <0.001 |

n=32437

Models were adjusted by age, sex, race, education, income, physical activity, smoke, alcohol, DASH, PRS, and history of diabetes mellitus, hypertension, other CVD, and cancer. DASH: Dietary approaches to stop hypertension; PRS: Polygenic risk score; CVD: Cardiovascular diseases; CMR: Cardiac magnetic resonance imaging; LV-EDV: Left ventricularend diastolic volume; LV-ESV: Left ventricularend systolic volume; LV-SV: Left ventricularstroke volume; LV-EF: Left ventricularejection fraction; RV-EDV: Right ventricularend diastolic volume; RV-ESV: Right ventricularend systolic volume; RV-SV: Right ventricularstroke volume; RV-EF: Right ventricularejection fraction; LAVmax: Left atrial maximum volume; LAVmin: Left atrial minimum volume; LA-SV: Left atrial stroke volume; LA-EF: Left atrial ejection fraction; RAVmax: Right atrial maximum volume; RAVmin: Right atrial minimum volume; RA-SV: Right atrial stroke volume; RA-EF: Right atrial ejection fraction; TyG: Triglyceride-glucose index; TyG-BMI: TyG-body mass index; TyG-WC: TyG-waist circumference; TyG-WHtR: TyG-waist-to-height ratio.

**Table S21. Sensitivity analysis of mediating roles of cardiac structure and function in the association between TyG obesity-related derivatives and atrial fibrillation after excluding participants with a history of CVD.**

| CMR | Effect | TyG-BMI | | TyG-WC | | TyG-WHtR | |
| --- | --- | --- | --- | --- | --- | --- | --- |
|  |  | HR(95%CI) | *P* | HR(95%CI) | *P* | HR(95%CI) | *P* |
| LV-EDV | Total effect | 1.348(1.238,1.579) | <0.001 | 1.72(1.581,1.91) | <0.001 | 1.36(1.264,1.528) | <0.001 |
|  | Direct effect | 1.177(1.074,1.39) | <0.001 | 1.399(1.265,1.579) | <0.001 | 1.204(1.145,1.365) | <0.001 |
|  | Indirect effect | 1.146(1.115,1.16) | <0.001 | 1.229(1.181,1.25) | <0.001 | 1.129(1.101,1.137) | <0.001 |
|  | Mediation (%) | 49.31(32.81,69.42) | <0.001 | 44.59(36.34,54.43) | <0.001 | 43.18(31.07,47.79) | <0.001 |
| LV-ESV | Total effect | 1.344(1.238,1.572) | <0.001 | 1.712(1.576,1.905) | <0.001 | 1.358(1.263,1.524) | <0.001 |
|  | Direct effect | 1.205(1.112,1.42) | <0.001 | 1.429(1.315,1.605) | <0.001 | 1.223(1.152,1.375) | <0.001 |
|  | Indirect effect | 1.115(1.099,1.126) | <0.001 | 1.198(1.173,1.217) | <0.001 | 1.11(1.096,1.124) | <0.001 |
|  | Mediation (%) | 40.3(26.97,53.19) | <0.001 | 39.7(33.03,45.29) | <0.001 | 37.7(28.49,42.18) | <0.001 |
| LV-SV | Total effect | 1.357(1.242,1.584) | <0.001 | 1.751(1.61,1.936) | <0.001 | 1.369(1.27,1.537) | <0.001 |
|  | Direct effect | 1.276(1.159,1.493) | <0.001 | 1.617(1.454,1.798) | <0.001 | 1.301(1.223,1.465) | <0.001 |
|  | Indirect effect | 1.063(1.038,1.081) | <0.001 | 1.083(1.044,1.108) | <0.001 | 1.052(1.031,1.063) | <0.001 |
|  | Mediation (%) | 22.65(13.28,36.45) | <0.001 | 17.79(9.88,25.64) | <0.001 | 18.45(10.32,23.14) | <0.001 |
| LV-EF | Total effect | 1.356(1.24,1.586) | <0.001 | 1.732(1.595,1.926) | <0.001 | 1.365(1.262,1.532) | <0.001 |
|  | Direct effect | 1.303(1.19,1.537) | <0.001 | 1.582(1.457,1.762) | <0.001 | 1.295(1.195,1.451) | <0.001 |
|  | Indirect effect | 1.041(1.032,1.042) | <0.001 | 1.095(1.086,1.1) | <0.001 | 1.054(1.049,1.059) | <0.001 |
|  | Mediation (%) | 14.85(8.45,20.78) | <0.001 | 20.46(17.54,23.31) | <0.001 | 19.17(15.05,25.49) | <0.001 |
| RV-EDV | Total effect | 1.36(1.245,1.59) | <0.001 | 1.742(1.6,1.934) | <0.001 | 1.371(1.269,1.542) | <0.001 |
|  | Direct effect | 1.2(1.083,1.435) | <0.001 | 1.45(1.288,1.679) | <0.001 | 1.22(1.147,1.401) | <0.001 |
|  | Indirect effect | 1.134(1.107,1.159) | <0.001 | 1.201(1.152,1.242) | <0.001 | 1.123(1.1,1.142) | <0.001 |
|  | Mediation (%) | 44.56(26.47,67.27) | <0.001 | 39.27(27.43,52.01) | <0.001 | 40.55(25.97,48.97) | <0.001 |
| RV-ESV | Total effect | 1.36(1.249,1.598) | <0.001 | 1.718(1.579,1.914) | <0.001 | 1.368(1.269,1.543) | <0.001 |
|  | Direct effect | 1.159(1.056,1.38) | <0.001 | 1.335(1.182,1.527) | <0.001 | 1.172(1.104,1.336) | <0.001 |
|  | Indirect effect | 1.174(1.152,1.197) | <0.001 | 1.287(1.248,1.336) | <0.001 | 1.167(1.145,1.191) | <0.001 |
|  | Mediation (%) | 55.83(36.57,78.61) | <0.001 | 53.33(42.47,68.61) | <0.001 | 53.26(38.16,62.67) | <0.001 |
| RV-SV | Total effect | 1.361(1.244,1.586) | <0.001 | 1.757(1.618,1.943) | <0.001 | 1.374(1.273,1.542) | <0.001 |
|  | Direct effect | 1.317(1.198,1.572) | <0.001 | 1.698(1.534,1.949) | <0.001 | 1.335(1.244,1.528) | <0.001 |
|  | Indirect effect | 1.033(1.008,1.046) | <0.001 | 1.035(0.997,1.054) | 0.4 | 1.03(1.009,1.038) | <0.001 |
|  | Mediation (%) | 12.19(2.4,21.63) | <0.001 | 7.83(-0.56,13.52) | 0.4 | 10.58(2.56,14.26) | <0.001 |
| RV-EF | Total effect | 1.359(1.25,1.594) | <0.001 | 1.732(1.587,1.917) | <0.001 | 1.362(1.266,1.528) | <0.001 |
|  | Direct effect | 1.274(1.168,1.494) | <0.001 | 1.544(1.399,1.696) | <0.001 | 1.266(1.175,1.41) | <0.001 |
|  | Indirect effect | 1.067(1.06,1.07) | <0.001 | 1.122(1.11,1.137) | <0.001 | 1.076(1.067,1.086) | <0.001 |
|  | Mediation (%) | 23.71(16.93,32.54) | <0.001 | 25.69(24.14,32.27) | <0.001 | 26.53(22.48,34.18) | <0.001 |
| LAVmax | Total effect | 1.312(1.204,1.541) | <0.001 | 1.643(1.522,1.798) | <0.001 | 1.302(1.213,1.457) | <0.001 |
|  | Direct effect | 1.097(1.015,1.275) | <0.001 | 1.369(1.281,1.491) | <0.001 | 1.148(1.082,1.287) | <0.001 |
|  | Indirect effect | 1.196(1.182,1.211) | <0.001 | 1.201(1.18,1.208) | <0.001 | 1.133(1.121,1.133) | <0.001 |
|  | Mediation (%) | 68.82(49.69,92.88) | <0.001 | 42.68(38.47,46.75) | <0.001 | 50.77(37.27,62.05) | <0.001 |
| LAVmin | Total effect | 1.282(1.145,1.533) | <0.001 | 1.589(1.454,1.727) | <0.001 | 1.247(1.131,1.401) | <0.001 |
|  | Direct effect | 1.166(1.042,1.376) | <0.001 | 1.435(1.323,1.545) | <0.001 | 1.167(1.063,1.309) | <0.001 |
|  | Indirect effect | 1.099(1.096,1.115) | <0.001 | 1.107(1.098,1.119) | <0.001 | 1.068(1.064,1.07) | <0.001 |
|  | Mediation (%) | 41(29.85,71.43) | <0.001 | 26.1(24.92,28.86) | <0.001 | 32.13(22.97,53.08) | <0.001 |
| LA-SV | Total effect | 1.36(1.244,1.585) | <0.001 | 1.757(1.618,1.944) | <0.001 | 1.375(1.273,1.542) | <0.001 |
|  | Direct effect | 1.36(1.246,1.591) | <0.001 | 1.765(1.613,1.957) | <0.001 | 1.374(1.274,1.542) | <0.001 |
|  | Indirect effect | 1(0.979,1.007) | 0.4 | 0.996(0.976,1.004) | 0.4 | 1.001(0.986,1.006) | 0.8 |
|  | Mediation (%) | -0.09(-7.79,3.68) | 0.4 | -1.02(-5.78,1.02) | 0.4 | 0.36(-4.71,2.13) | 0.8 |
| LA-EF | Total effect | 1.281(1.112,1.496) | <0.001 | 1.602(1.49,1.736) | <0.001 | 1.271(1.13,1.428) | <0.001 |
|  | Direct effect | 1.267(1.09,1.49) | <0.001 | 1.571(1.478,1.718) | <0.001 | 1.282(1.139,1.464) | <0.001 |
|  | Indirect effect | 1.011(1.004,1.021) | <0.001 | 1.02(1.003,1.018) | <0.001 | 0.992(0.975,0.992) | <0.001 |
|  | Mediation (%) | 5.16(1.32,21.53) | <0.001 | 5.23(0.77,5.13) | <0.001 | -3.99(-8.76,-3.94) | <0.001 |
| RAVmax | Total effect | 1.367(1.254,1.601) | <0.001 | 1.697(1.545,1.891) | <0.001 | 1.398(1.284,1.577) | <0.001 |
|  | Direct effect | 1.336(1.217,1.565) | <0.001 | 1.497(1.345,1.682) | <0.001 | 1.372(1.271,1.551) | <0.001 |
|  | Indirect effect | 1.023(1.016,1.033) | <0.001 | 1.133(1.119,1.15) | <0.001 | 1.019(1.01,1.024) | <0.001 |
|  | Mediation (%) | 8.53(5.96,15.27) | <0.001 | 28.62(23.4,36.82) | <0.001 | 6.54(4.07,8.9) | <0.001 |
| RAVmin | Total effect | 1.366(1.237,1.594) | <0.001 | 1.677(1.533,1.867) | <0.001 | 1.392(1.269,1.573) | <0.001 |
|  | Direct effect | 1.318(1.184,1.542) | <0.001 | 1.431(1.295,1.601) | <0.001 | 1.336(1.227,1.52) | <0.001 |
|  | Indirect effect | 1.036(1.027,1.046) | <0.001 | 1.172(1.159,1.186) | <0.001 | 1.042(1.033,1.05) | <0.001 |
|  | Mediation (%) | 12.99(8.78,22.52) | <0.001 | 36.42(30.68,44.89) | <0.001 | 14.26(8.94,18.68) | <0.001 |
| RA-SV | Total effect | 1.363(1.247,1.588) | <0.001 | 1.756(1.61,1.944) | <0.001 | 1.376(1.274,1.542) | <0.001 |
|  | Direct effect | 1.362(1.247,1.586) | <0.001 | 1.732(1.575,1.931) | <0.001 | 1.38(1.28,1.544) | <0.001 |
|  | Indirect effect | 1.001(1,1.003) | 0.4 | 1.014(1.007,1.022) | <0.001 | 0.997(0.994,0.999) | <0.001 |
|  | Mediation (%) | 0.26(-0.19,1.22) | 0.4 | 3.14(1.36,5.68) | <0.001 | -1.26(-2.3,-0.25) | <0.001 |
| RA-EF | Total effect | 1.357(1.238,1.59) | <0.001 | 1.748(1.615,1.943) | <0.001 | 1.369(1.262,1.54) | <0.001 |
|  | Direct effect | 1.309(1.2,1.552) | <0.001 | 1.562(1.451,1.751) | <0.001 | 1.293(1.192,1.477) | <0.001 |
|  | Indirect effect | 1.037(1.024,1.041) | <0.001 | 1.119(1.104,1.133) | <0.001 | 1.059(1.041,1.07) | <0.001 |
|  | Mediation (%) | 13.42(6.54,17.8) | <0.001 | 24.85(20.04,27.72) | <0.001 | 20.65(11.45,27.09) | <0.001 |

n=31333

Models were adjusted by age, sex, race, education, income, physical activity, smoke, alcohol, DASH, PRS, and history of diabetes mellitus, hypertension, and cancer. DASH: Dietary approaches to stop hypertension; PRS: Polygenic risk score; CVD: Cardiovascular diseases; CMR: Cardiac magnetic resonance imaging; LV-EDV: Left ventricularend diastolic volume; LV-ESV: Left ventricularend systolic volume; LV-SV: Left ventricularstroke volume; LV-EF: Left ventricularejection fraction; RV-EDV: Right ventricularend diastolic volume; RV-ESV: Right ventricularend systolic volume; RV-SV: Right ventricularstroke volume; RV-EF: Right ventricularejection fraction; LAVmax: Left atrial maximum volume; LAVmin: Left atrial minimum volume; LA-SV: Left atrial stroke volume; LA-EF: Left atrial ejection fraction; RAVmax: Right atrial maximum volume; RAVmin: Right atrial minimum volume; RA-SV: Right atrial stroke volume; RA-EF: Right atrial ejection fraction; TyG: Triglyceride-glucose index; TyG-BMI: TyG-body mass index; TyG-WC: TyG-waist circumference; TyG-WHtR: TyG-waist-to-height ratio.
